# Machine learning model of the catalytic efficiency and substrate specificity of acyl-ACP thioesterase variants generated from natural and *in vitro* directed evolution

**DOI:** 10.3389/fbioe.2024.1379121

**Published:** 2024-04-11

**Authors:** Fuyuan Jing, Keting Chen, Marna D. Yandeau-Nelson, Basil J. Nikolau

**Affiliations:** ^1^ Roy J. Carver Department of Biochemistry, Biophysics, and Molecular Biology, Iowa State University, Ames, IA, United States; ^2^ Center for Metabolic Biology, Iowa State University, Ames, IA, United States; ^3^ Engineering Research Center for Biorenewable Chemicals, Iowa State University, Ames, IA, United States; ^4^ Department of Genetics, Development and Cell Biology, Iowa State University, Ames, IA, United States

**Keywords:** Thioesterase, acyl-ACP, fatty acids, directed evolution, random forest, machine learning

## Abstract

Modulating the catalytic activity of acyl-ACP thioesterase (TE) is an important biotechnological target for effectively increasing flux and diversifying products of the fatty acid biosynthesis pathway. In this study, a directed evolution approach was developed to improve the fatty acid titer and fatty acid diversity produced by *E. coli* strains expressing variant acyl-ACP TEs. A single round of *in vitro* directed evolution, coupled with a high-throughput colorimetric screen, identified 26 novel acyl-ACP TE variants that convey up to a 10-fold increase in fatty acid titer, and generate altered fatty acid profiles when expressed in a bacterial host strain. These *in vitro-*generated variant acyl-ACP TEs, in combination with 31 previously characterized natural variants isolated from diverse phylogenetic origins, were analyzed with a random forest classifier machine learning tool. The resulting quantitative model identified 22 amino acid residues, which define important structural features that determine the catalytic efficiency and substrate specificity of acyl-ACP TE.

## 1 Introduction

Human civilization has been enabled by our ability to harness and uniquely utilize outputs from biological systems (Holdren and Ehrlich, 1974). Via the domestication of animal, plant and microbial life forms (Stetter et al., 2017), we have developed technologies that support the ability of the human species to colonize nearly all niches that are available on the earth, and we are now contemplating technologies to colonize niches beyond the confines of our planet. Paramount to the growth of human civilization has been the ability to have ample food supply and the ability to harness energy from the environment that supports these activities. Since the start of the industrial revolution in the 18th century, with the invention of the steam engine, we have become increasingly dependent on the oxidation of fossil carbon, first in the form of coal, and subsequently liquid (i.e., oil) and gaseous (i.e., natural gas) forms of fossil carbon. In parallel to these energy-generating carbon-oxidation processes, we have developed technologies that convert fossil carbon to materials that support our modern forms of life (i.e., the petrochemical industry).

Over the past 250 years, these activities have increasingly disrupted the earth’s ecological carbon-balance that has taken millions of years to reach equilibrium. Thus, we now face the increasing challenge of carbon in the atmosphere (CO_2_, CO, CH_4_), which contributes to global warming and climate change (Andrew, 2020), and the earth’s land and ocean environments are increasingly polluted by non-degradable carbon polymers (e.g., single use plastics) (Barnes, 2019). In response, there have been increasing research efforts to adapt or engineer biological systems as platforms for generating biorenewable chemicals or biofuels generated from photosynthetically fixed CO_2_ (Nikolau et al., 2008; Chandel et al., 2020). Although global biological photosynthesis can fix sufficient quantities of atmospheric CO_2_ to meet current needs for fuels and chemicals, most of that biological carbon occurs in the form of lignocellulosic material (Limayem and Ricke, 2012). But unlike fossil carbon, which is chemically highly reduced carbon that lacks oxygen, and is thus energy dense, lignocellulosic carbon is partially oxidized, and thus has lower energy density.

Fatty acids, in contrast, contain less oxygen and are energy dense, and are therefore more similar to fossil carbon feedstocks, particularly petroleum. Therefore, there’s been considerable interest in converting lignocellulosic carbon (e.g., sugars) to fatty acids, chemically removing oxygen and increasing the energy density of the product. In biological systems, fatty acids are stored as triacylglycerol in plant seeds, single cell microbes, animal adipocytes or milk products. Societal consumption of these natural products occurs not only via the food supply, but also as industrial feedstocks of ingredients such as soaps, detergents, surfactants, lubricants, cosmetics, and pharmaceuticals (Ohlrogge, 1994; Thelen and Ohlrogge, 2002; Dyer et al., 2008; Parsons and Rock, 2013). With the rising cost of petroleum and growing environmental concerns about oxidizing large amounts of fossil carbon, the beginning of the 21st century has seen increasing interest in using biological fatty acids for the production of biofuels or chemical feedstocks (Durrett et al., 2008; Nikolau et al., 2008; Santner et al., 2023).

In plants and bacteria, fatty acid biosynthesis is catalyzed by a Type II fatty acid synthase (FAS), using acetyl-CoA and malonyl-ACP as substrates. This process proceeds via the iterative cycle of four reactions (condensation-reduction-dehydration and reduction), which together elongate the acyl-chain by 2-carbon atoms per cycle. The substrate intermediates throughout this process are esterified to the thiol group of a phosphopantetheinyl cofactor, carried by acyl carrier-protein (ACP). This elongation process can be terminated by either the transacylation of the acyl-chain to a glycerol backbone to begin the process of membrane glycerolipid assembly, or by the hydrolysis of the thioester bond of acyl-ACP, catalyzed by acyl-ACP thioesterase (TE), to release a free fatty acid. Many acyl-ACP TEs have been isolated and characterized, and they exhibit different fatty acyl chain length specificities, and thus play a crucial role in determining the chain lengths of the fatty acid products generated by plant and bacterial FAS systems (Pollard et al., 1991; Voelker et al., 1992; Leonard et al., 1998; Serrano-Vega et al., 2005; Lennen and Pfleger, 2012; Pfleger et al., 2015).

Four oil seed crops (i.e., palm, soybean, canola, and sunflower) generate 80% of the world’s 220 million metric tons of vegetable oils (https://ourworldindata.org/grapher/vegetable-oil-production). These oils serve as feedstocks for both dietary needs and as precursors for industrial applications (Kumar et al., 2016; Savva and Kafatos, 2016; Huang et al., 2021). The fatty acids obtained from these oils have relatively narrow chemical diversity, primarily providing fatty acids of 16- and 18-carbon chain lengths with different degrees of unsaturation (i.e., 0 to 3 carbon-carbon double bonds). In contrast, seeds of a few discrete phylogenetic plant clades (e.g., palm, coconut and cuphea) are the source of the world’s 12 million metric tons of lauric acid-containing oils, which are the primary feedstocks for the soap and detergent industry (Smith, 2019).

In more recent years, with better understanding of the regulation of the fatty acid biosynthesis pathway, and the rapid advances in synthetic biology, there has been intense interest in the metabolic engineering of this pathway for the production of fatty acids of different chain lengths or different fatty acid derivatives (Handke et al., 2011; Liu et al., 2011; Zhang et al., 2011; Lennen and Pfleger, 2012; Ranganathan et al., 2012; Zhang et al., 2012; Heil et al., 2019). These efforts have focused on increasing the titers and chemical diversity of fatty acids, and have concentrated on using three biological chassis: plant seeds (Dehesh et al., 1996; Inckemann, 2022), bacteria (Pfleger et al., 2015; Adams, 2016) and yeast (Gajewski et al., 2017; Schindler, 2020). Because of genetic tractability, the bacterial chassis has primarily focused on *Escherichia coli*, and two approaches for increasing fatty acid titers have been demonstrated, either independently or in combination. One is the overexpression of acyl-ACP TEs, which release free fatty acids from the FAS system, and another is the elimination of the fatty acid β-oxidation pathway via mutations of either *fadD* (acyl-CoA synthetase) or *fadE* (acyl-CoA dehydrogenase) (Lennen and Pfleger, 2012; Heil et al., 2019).

The expression of acyl-ACP TEs in bacterial systems confers two novel attributes. Based on the substrate specificity of the acyl-ACP TE that is used, one can control the acyl chain lengths of the fatty acids that the FAS system will produce (Voelker and Davies, 1994; Jing et al., 2011). In addition, expression of acyl-ACP TEs enhances fatty acid titer of the resulting strain by creating a new metabolic product-sink for the FAS pathway, and by depleting the *in vivo* long chain acyl-ACP pool size, which relieves feedback inhibition of upstream enzymes in the FAS pathway (Jiang and Cronan, 1994; Heath and Rock, 1996; Lennen and Pfleger, 2012). These attributes can be optimized by controlling the expression of acyl-ACP TEs by using expression plasmid vectors with different promoter strengths, and/or different plasmid copy numbers (Steen et al., 2010; Youngquist et al., 2012; Zhang et al., 2012). Thus, prior studies have used acyl-ACP TEs sourced from a variety of different natural sources, each of which has evolved for that organism’s environmental niche. However, that natural evolutionary adaptation may not be optimal for the envisioned industrial application in a heterologous host, such as an *E. coli* strain bioengineered for increased fatty acid titers or production of a fatty acid of a specific acyl chain length.

Limited structural information and a not well-understood catalytic mechanism for acyl-ACP TEs (Mayer and Shanklin, 2005; Serrano-Vega et al., 2005; Feng et al., 2017; Jing et al., 2018a) make it challenging to increase the activity of this enzyme by rational design. Directed evolution is an alternative approach that mimics the natural evolutionary process at the lab scale, and provides a strategy to identify and exploit genetic space that natural evolution may not have explored (Dougherty and Arnold, 2009; Turner, 2009; Cobb et al., 2013). Directed evolution involves iterative rounds of random mutagenesis and screening for the desired biological properties. This strategy has been successfully applied on a number of biocatalysts to tailor their functions, including substrate specificity, catalytic turnover, and thermostability (Nair and Zhao, 2008; Zha et al., 2008; Turner, 2009), including acyl-ACP TEs (Feng et al., 2017; Hernández Lozada et al., 2018).

In this study, directed evolution was undertaken to optimize the catalytic efficiency of acyl-ACP TE with the goal of improving fatty acid titers in microbes. Specifically, we selected six previously well-characterized plant acyl-ACP TEs as parental enzymes that display diverse catalytic efficiencies and substrate specificities (Jing et al., 2011), and used a PCR-based approach to generate a library of acyl-ACP TE variants. *In vivo* screening of this library for individual variants that express higher fatty acid titers enabled the isolation and characterization of novel acyl-ACP TEs that exhibit improved catalytic efficiency, as compared to the initial parental acyl-ACP TEs. These novel enzymes were found to also express diverse substrate specificities relative to the acyl-chain length of the preferred acyl-ACP substrate. Taking advantage of these acyl-ACP TE variants and other functionally characterized acyl-ACP TEs reported in our prior studies (Jing et al., 2011; Jing et al., 2018b), we implemented and optimized a random forest-directed approach that ranked the importance of each residue in determining acyl-ACP TE catalytic efficiency and substrate specificity, providing a quantitative basis for additional directed evolution strategies.

## 2 Materials and methods

### 2.1 Design of mutagenesis oligonucleotides

Amino acid sequences of the six acyl-ACP TEs that were used in this study are: CvFatB1 (AEM72522.1) and CvFatB2 (AEM72523.1) from *Cuphea viscosissima*; CnFatB2 (AEM72520.1) and CnFatB3 (AEM72521.1) from *Cocos nucifera*; UaFatB1 (AAB71731.1) from *Ulmus americana*; and CpFatB1 (AAC49179.1) from *Cuphea palustris.* Supplementary Figure S1 shows comparisons of the sequences of these 6 TE proteins (without the N-terminal chloroplast targeting sequences). Random mutagenesis was used to generate 2–8 possible substitutions at 98 selected positions. These 98 positions were primarily selected for convenience in the design of the primers used to reassemble the acyl-ACP TE variant library. The variant library was generated by PCR reassembly of mutant acyl-ACP TEs by using 30 DNA oligonucleotide primers (labeled as M1-1 to M1-10, M2-1 to M2-10, and M3-1 to M3-10) that incorporated mixed nucleotides at each of the 98 selected positions (Supplementary Table S1). The ends of each of these 30 oligonucleotide primers overlapped with the adjoining oligonucleotide sequences by 22–25 nucleotides; the Tm values for these overlapping regions were in the range of 54°C–56°C. In addition, the 5′- and 3′-ends of oligonucleotides M1-1 and M3-10 encoded *Bam*HI and *Eco*RI restriction sites, respectively. These characteristics enabled PCR-based reassembly of the entire acyl-ACP TE sequence into a single DNA fragment, which contained terminal *Bam*HI and *Eco*RI restriction sites for subsequent cloning purposes.

### 2.2 PCR-assembly of the variant acyl-ACP TE library

The acyl-ACP TE-encoding variant library was generated by assembling the 30 oligonucleotide primers by two rounds of PCR. The first round of PCR was conducted in a 50 μL reaction mix containing 0.15 μM of each primer (primers M1-1 to M1-10, M2-1 to M2-10, and M3-1 to M3-10), commercial Taq PCR buffer (New England Biolabs, M0273), 0.4 mM dNTP, 3 mM MgCl_2_, and 1 Unit of Taq DNA polymerase (New England Biolabs, United States). The thermal cycling program for the first round of PCR was initiated by incubating the mix at 95°C for 3 min, and then 25 cycles of incubations at 95°C for 15 s, 50°C for 20 s and 68°C for 40 s; the final extension step was at 68°C for 5 min. Two-μL aliquots of product from the first round of PCR were used as the template for the second round of PCR. This second round of PCR consisted of a 50 μL reaction mixture containing 0.2 μM of primer M1-1 and 0.2 μM of primer M3-10, commercial Taq PCR buffer (New England Biolabs, M0273), 0.2 mM dNTP, 1.5 mM MgCl_2_ and 1 Unit Taq DNA polymerase. The thermal cycling program began at 95°C for 3 min, and then 28 cycles of 95°C for 15 s, 60°C for 20 s and 68°C for 40 s, and a final 5-min extension step at 68°C.

Products from the second round of PCR were fractionated by electrophoresis in a 1% agarose gel, and the 950 bp DNA fragment was purified with the QiaQuick gel extraction kit (Qiagen, Valencia, CA, United States). The recovered DNA was digested with *Bam*HI and *Eco*RI, and cloned into the corresponding restriction sites of the vector, pUCHisGm (Supplementary Figure S2); this plasmid was specifically modified from pUC57 in this study. In this vector, the expression of the acyl-ACP TE sequence is under the transcriptional control of the *lacZ* promoter, and the acyl-ACP TE coding sequence is fused at the N-terminus to a 6x His-tag, and at the C-terminus it was fused to a gentamicin resistant gene (Gm^R^) that is separated from the acyl-ACP TE coding sequence via a dodecapeptide flexible linker-sequence, [(Gly)_3_-Ser]_3_ (Chen et al., 2012). The resulting mixture of plasmid vectors containing the variant acyl-ACP TE ORFs were transformed into *E. coli* K27 by electroporation. Hence, each recovered colony from this transformation event carried a plasmid that has the potential of expressing an individual variant acyl-ACP TE. As controls, the DNA fragments of the six mature wild-type acyl-ACP TE-coding sequences (UaFatB1, CpFatB1, CvFatB1, CvFatB2, CnFatB2, and CnFatB3) were also cloned into pUCHisGm and transformed into *E coli* strain K27.

### 2.3 Colony screening of acyl-ACP TE variants by Neutral Red staining

The initial screening of the acyl-ACP TE variants was conducted on solid media containing the pH indicator stain, Neutral Red. These 10-cm diameter Petri plates contained M9 minimal medium (50 mM Na_2_HPO_4_, 20 mM KH_2_PO_4_, 10 mM NaCl, 20 mM NH_4_Cl, 2 mM MgSO_4_, and 0.1 mM CaCl_2_) solidified with 15 g/L agar and supplemented with 0.4% glucose, 100 mg/L carbenicillin, 2.5 mg/L gentamicin, 1 mM isopropyl-β-D-thiogalactopyranoside (IPTG), and 100 ppm Neutral Red dye. Each Neutral Red plate was inoculated with an appropriate amount of the electroporation-transformation mixture so that each plate supported the growth of 300–500 colonies. Upon inoculation these plates were incubated at 30°C for 3 days, and colonies that showed the most intense red color were selected for further characterizations.

### 2.4 Analysis of fatty acids by gas chromatography-mass spectrometry

Intensely red-staining colonies were selected from the Neutral Red plates, inoculated into 0.7 mL of LB medium supplemented with 100 mg/L carbenicillin, and cultured overnight at 30°C at a 250 rpm agitation rate. A 0.1 mL aliquot of the overnight culture was used to inoculate 2 mL M9 medium supplemented with 2% glucose, 100 mg/L carbenicillin and 0.1 mM IPTG in 16-mL culture-tubes. After incubating at 30°C with agitation at 250 rpm for 48 h, a 1.5 mL aliquot of the culture was used for fatty acid extraction. Following the addition of 50 μg heptanoic acid (7:0), 50 μg undecanoic acid (11:0), and 100 μg heptadecanoic acid (17:0) (Sigma-Aldrich, St. Louis, MO, United States) as internal standards, the mixture was acidified with 0.5 mL of 1 M HCl, and 4 mL chloroform-methanol (1:1 vol/vol) was used to extract and recover the fatty acids from the culture. After vortexing for 10 min, and centrifugation at 3000 *g* for 4 min, the lower chloroform phase was passed through an anhydrous MgSO_4_ column to remove trace amounts of water, and the volume of the recovered solution was reduced to approximately 0.2 mL by evaporation under a stream of N_2_ gas. The samples were subjected to fatty acid analysis by GC-MS (Jing et al., 2011). Control fatty acid profiles produced by *E. coli* cultures that harbored the non-modified pUCHisGm vector were subtracted from the fatty acid profiles produced by each acyl-ACP TE variant.

### 2.5 Statistical analysis, random forest classification and model performance prediction

Fatty acid titer and composition data obtained with each acyl-ACP TE variant were assessed by analysis of variance (ANOVA) and *post hoc* Tukey’s Honestly Significant Difference (HSD) tests using JMP, Version 15 (SAS Institute Inc., Cary, NC). Principal Component Analysis (PCA) was performed using the prcomp () function in the R/stats package and 95% confidence ellipses were constructed using the dataEllipse function in the R/car package (Fox and Weisberg, 2011).

For the machine learning approach, the random forest classifier was applied to calculate the relative importance of individual amino acid residues in determining the substrate specificity of acyl-ACP TE (Wang et al., 2012; Basu et al., 2017; Luttrell et al., 2019). The strategy used sequence variants of acyl-ACP TE enzymes, whose *in vivo* catalytic capabilities were quantitatively evaluated in *E. coli*. The instances used to construct the random forest classifier included the comparison of substrate specificity (response) and sequence variation (feature) between any two acyl-ACP TEs (Supplementary Figure S4). The features of the classifier were defined based on the multiple sequence alignment among all acyl-ACP TE variants and the sequence variation between any two acyl-ACP TEs was represented as a vector using a binary scoring method, where the value “0” is assigned at an amino acid position if two acyl-ACP TEs have the same residue, and the value “1” is assigned if they have different residues at that position (Supplementary Figure S4).

The responses (i.e., the pairwise comparison of substrate specificities) were binary scores defined based on the clustering analysis of fatty acid profiles. The fatty acid profiles were first normalized so that the average concentrations of all individual fatty acids were mathmetically converted to a value of 0, and the associated standard deviation was converted to a value of 1. Next, Ward’s hierarchical clustering analysis (Ward, 1963) was performed based on the Euclidean distances of the scaled fatty acid profiles, using the hclust function in the R/stat package (R core team, 2020). The resultant dendrogram was pruned to determine the enzyme cluster membership by the cutreedynamic function using the method “hybrid” in the R package “dynamicTreeCut” (Langfelder and Horvath, 2008). Any 2 TEs belonging to the same cluster were deemed to have similar substrate specificities and assigned the value “0”. Acyl-ACP TE pairs belonging to different clusters were categorized as having different substrate specificities and assigned the value “1” (Supplementary Figure S4).

Random forest classification models were constructed using the R package, “ranger” (Wright and Ziegler, 2017). The training phase included the construction of 500 decision trees using gini impurity (i.e., the probability of misclassifying the substrate specificity relationship between two acyl-ACP TEs) as the node-split criteria for each tree (Guyon and Elisseeff, 2003). The prediction of a random forest model is made by pooling the predictions from all trees. Feature importance scores for each residue position of the enzyme, including a randomly-generated position (i.e., a control feature), were calculated based on the total decrease in node gini-impurity averaged over the 500 trees. These calculations provide a quantitative measure of the importance of each residue in classifying the enzyme pairs into two classes, i.e., the pair of enzymes that each express the same or different catalytic capabilities. The importance scores and the associated *p*-values were calculated using the importance_pvalues function in the “ranger” package. The *p*-values were corrected across all residues by controlling the false discovery rate at <5% (Benjamini and Hochberg, 1995). To account for the randomness involved in the classifier construction, the random forest classifier was implemented ten times with the same dataset, and the average importance scores were calculated at each of the residue positions of the enzyme. The reported *p*-value for each position is presented as the maximum value of the ten classifiers.

To further refine the search for the important residue positions that determine enzyme substrate specificity, an incremental feature selection approach was used to identify the random forest classifier with a minimum number of features, but having an optimal predictive performance for substrate specificity. Briefly, the residue positions were ranked in descending order based on their importance scores. For incremental feature selection, an initial random forest model was constructed using the two residue positions with the highest importance scores as the features. Additional models were subsequently constructed by iteratively adding one position based on the importance score rank to the initial model. We then applied a ten-fold cross-validation to evaluate the predictive performance of the models. The predictive performance of each model was evaluated by the metrics of recall, specificity, and Matthews Correlation Coefficient (MCC). We define the enzyme pairs displaying the same substrate specificity as a negative instance (i.e., having a binary response score of 0), and the enzyme pairs displaying different substrate specificities as a positive instance (i.e., the binary response score being 1). The three evaluating metrics were calculated based on the number of true positives (TP), number of false negatives (FN), number of true negatives (TN), and number of false positives (FP) in a classification model, using the following formula:

$$\text{Recall}=\frac{\text{TP}}{\text{TP}+\text{FN}}$$

$$\text{Specificity}=\frac{\text{TN}}{\text{TN}+\text{FP}}$$

$$\text{MCC}=\frac{\left(\text{TP}\times \text{TN}\right)\;-\left(\text{FP}\times \text{FN}\right)\;}{\sqrt{\left(\text{TP}+\text{FP}\right)\left(\text{TP}+\text{FN}\right)\left(\text{TN}+\text{FP}\right)\left(\text{TN}+\text{FN}\right)}}$$

This incremental feature selection calculation was iterated 20 times, and the average values of recall, specificity, and MCC were deduced. The model with the highest MCC value is considered as the optimal model, and the residue positions included in this model were identified as the most significant residues that influence the enzyme’s substrate specificity. The R scripts used for hierarchical clustering analysis of fatty acid profiles, random forest classification and incremental feature selection strategy are available at: https://github.com/ketingchen/Acyl_ACP_TE_MachineLearning.

## 3 Results

### 3.1 Sequence polymorphisms encompassed by the acyl-ACP TE variant library

Six parental acyl-ACP TEs (i.e., CnFatB3, CvFatB1, CnFatB2, UaFatB1, CvFatB2, and CpFatB1) (Supplementary Figure S1) were selected to initiate the directed evolution study because prior characterizations had identified that these enzymes express diverse substrate specificities and generate diverse *in vivo* fatty acid titers upon expression in *E. coli* (Jing et al., 2011). The directed evolution strategy implemented herein generated variant enzymes that were initially screened for increased fatty acid titers in *E. coli*. Ninety-eight sequence polymorphisms (i.e., residue variations) that occur among the six parental acyl-ACP TEs were randomly recombined *in vitro* by a PCR-based reassembly of the acyl-ACP TE-coding sequences (See Methods).

An initial pilot study evaluated the diversity of the acyl-ACP TE sequences recoverable from the constructed variant library. In this pilot experiment, 47 colonies were randomly chosen from the initial transformants without the Neutral Red selection for enhanced fatty acid accumulation, and the acyl-ACP TE sequences were determined from the recovered plasmids. The sequences of these 47 variant acyl-ACP TEs all differ from each other and from the six parental acyl-ACP TE sequences that went into the design of the variant library. However, only two of the reassembled acyl-ACP TE sequences encode a fully translatable, full length acyl-ACP TE protein. The majority of the recovered mutants in this small sub-sample contained nonsense mutations (e.g., premature stop codon), or frame shifts due to an insertion or deletion of a single nucleotide. These are likely due to mis-alignments during PCR assembly.

### 3.2 Neutral Red colony-staining screen to identify hyperactive acyl-ACP TEs

**FIGURE 1:**
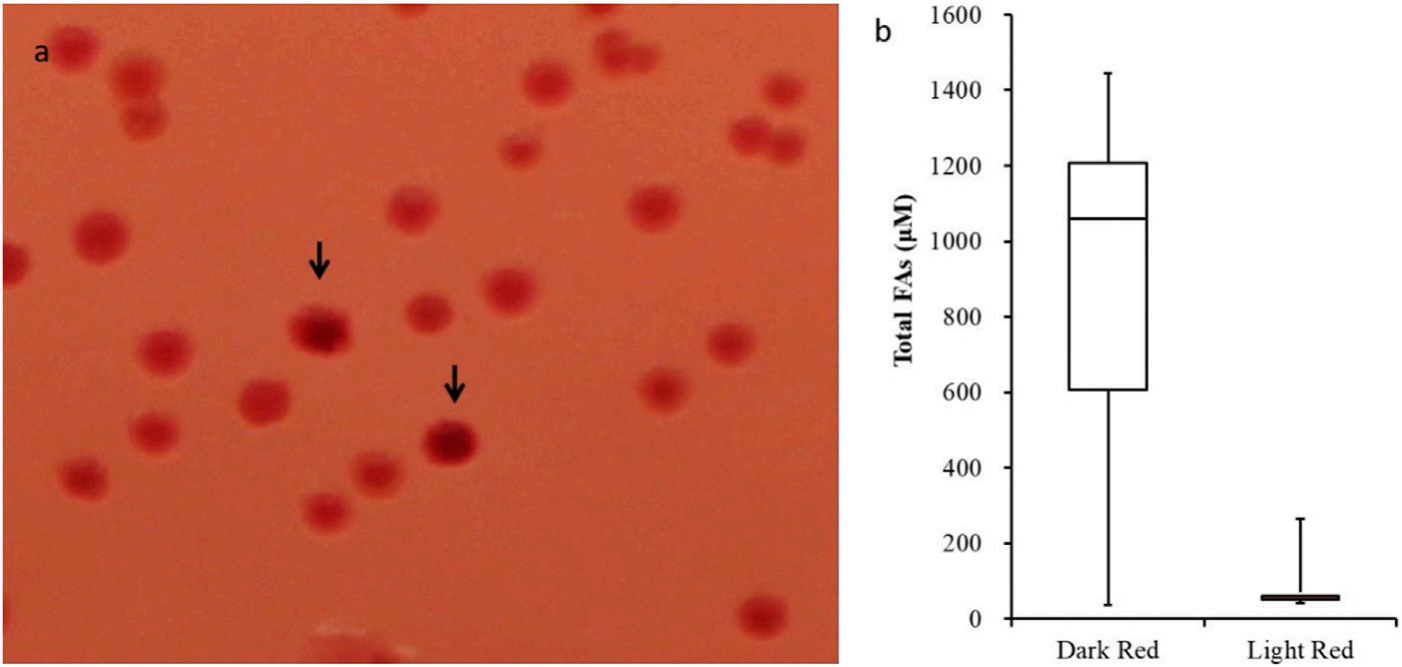
Efficacy of the Neutral Red plate screening assay. **(A)** Colonies expressing acyl-ACP TE variants were grown at 30°C for 3 days on Petri plates with media supplemented with Neutral Red dye. The colonies displaying a more intense red color are indicated by arrows. **(B)** Box-and-whisker plot of fatty acid titer of cultures that were inoculated from “dark-red” (*n* = 177) and “light-red” (*n* = 77) colonies. *t*-test *p*-value <0.01.

Prior studies established that the *E. coli* strain K27 host used to propagate the variant acyl-ACP TE library, which carries a mutation in acyl-CoA synthetase (*fadD*), results in the over-production of free fatty acids (Voelker and Davies, 1994). Indeed, when expressed in this strain there is a direct relationship between the levels of acyl-ACP TE activity and the titer of free fatty acids produced (Jing et al., 2018a; Jing et al., 2018b). Therefore, the acyl-ACP TE variant library was bulk screened by growing transformants on media plates supplemented with the pH indicator dye, Neutral Red. Because the higher accumulation of free fatty acids acidifies the media, the Neutral Red dye is a gauge of fatty acid accumulation within individual colonies. Figure 1A shows colonies on a typical Neutral Red-containing plate. The majority of the recovered colonies (−98%) displayed a light red/pink color, but about 2% of the colonies exhibited a more intense red color, indicative of acidification due to increased fatty acid accumulation.

Based on this rationale, we initially selected 133 dark red-staining colonies and 77 light red/pink colored colonies and determined the fatty acid titers generated by these strains. Among the 133 dark red-staining strains, 75% produced more than 600 µM of fatty acids, 50% produced more than 1000 µM of fatty acids, and 25% produced even more, reaching levels greater than 1200 µM of fatty acids (Figure 1B). In contrast, the majority of the strains identified as light red/pink colored colonies produced <100 µM of fatty acids; the maximum amount of fatty acid produced by these light red/pink colonies was 260 µM (Figure 1B). These results confirm that there is a positive correlation between the intensity of the color produced by Neutral Red staining of colonies and the fatty acid titers generated by these strains.

**FIGURE 2:**
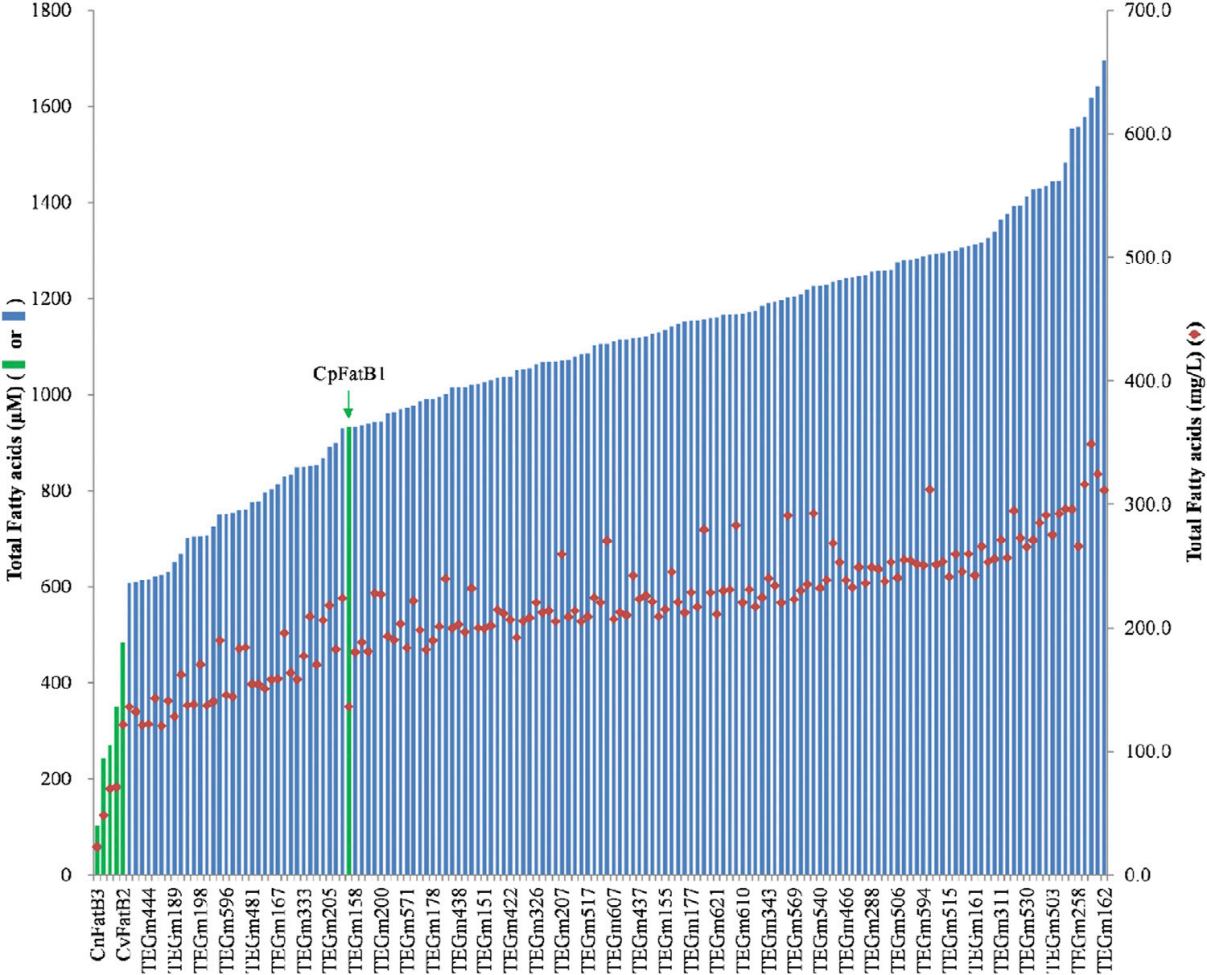
Fatty acid titers of six parental acyl-ACP TEs (green data-bars) and representative acyl-ACP TE variants (blue data-bars and red diamond data-points). Data-bars represent fatty acid titer data and are presented as µmol/L (data bars) and as mg/L (red-diamond data-points).

Ultimately, approximately 30,000 colonies were screened, which resulted in the selection of 480 strains that were expected to express a higher fatty acid titer based on enhanced Neutral Red staining (Supplementary Table S2). The fatty acid titers of these strains were determined and compared to the titers of the strains expressing the original six parental acyl-ACP TEs that were used as guides for the design of the acyl-ACP TE variant library. The fatty acid titer of the strains expressing these parental acyl-ACP TEs range between 100 μM and 900 µM (Figure 2, green data bars). Among the 480 colonies that were selected with the Neutral Red colony-staining assay, 151 expressed a fatty acid titer that is higher than 600 μM, ranging up to a maximum of 1700 µM (Supplementary Table S3). These titers are between 4- and 15-fold higher than five of the parental acyl-ACP TEs. Even compared to the most productive parental acyl-ACP TE (i.e., CpFatB1), the titer expressed by the variant acyl-ACP TEs are nearly 2-fold higher (Figure 2).

### 3.3 Sequences of acyl-ACP TE variants

The 174 acyl-ACP TE variants that expressed the highest *in vivo* fatty acid titers (ranging between 500 μM and 1700 µM) were sequenced. These sequences identified 26 distinct acyl-ACP TE variant proteins (Supplementary Figure S3). One of these variant proteins, TEGm162, recurred 147 times in the sequenced collection, TEGm204 was recovered 3 times, and TEGm198 was recovered twice; the remaining 22 sequences occurred uniquely in this collection (Supplementary Tables S2, S3). None of these recovered sequences identified by the Neutral Red staining screen were included among the original 47 randomly selected control sequence variants that were isolated without the Neutral Red-staining screen. Hence, these findings indicate that the Neutral Red staining screen has strong selection capability for acyl-ACP TE variants that express higher titers of fatty acids. The collective average of the fatty acid titers of the 147 independently-isolated TEGm162 variants was 1170 ± 210 μM, and the average for the three TEGm204 variants was 1100 ± 140 µM. These titers are −30% higher than the most effective parental acyl-ACP TE (i.e., CpFatB1), and 10-fold higher than the titer obtained with the least effective parental acyl-ACP TE (i.e., CnFatB3).

The sequences of the 26 distinct acyl-ACP TE variants selected by this directed evolution strategy (Supplementary Figure S3) were compared to each other and to the sequences of the six parental acyl-ACP TEs that were used to initiate this study. These analyses demonstrate that the recovered acyl-ACP TE variants share an overall sequence identity of −67%. Among the 307 amino acid positions of these recovered variant enzymes, polymorphisms occur at 100 positions, which is very close to the number of positions (i.e., 98) that we targeted for mutagenesis in the design of the variant library. The two additional polymorphic positions may be attributable to variants introduced by errors in DNA primer synthesis or by PCR errors.

**FIGURE 3:**
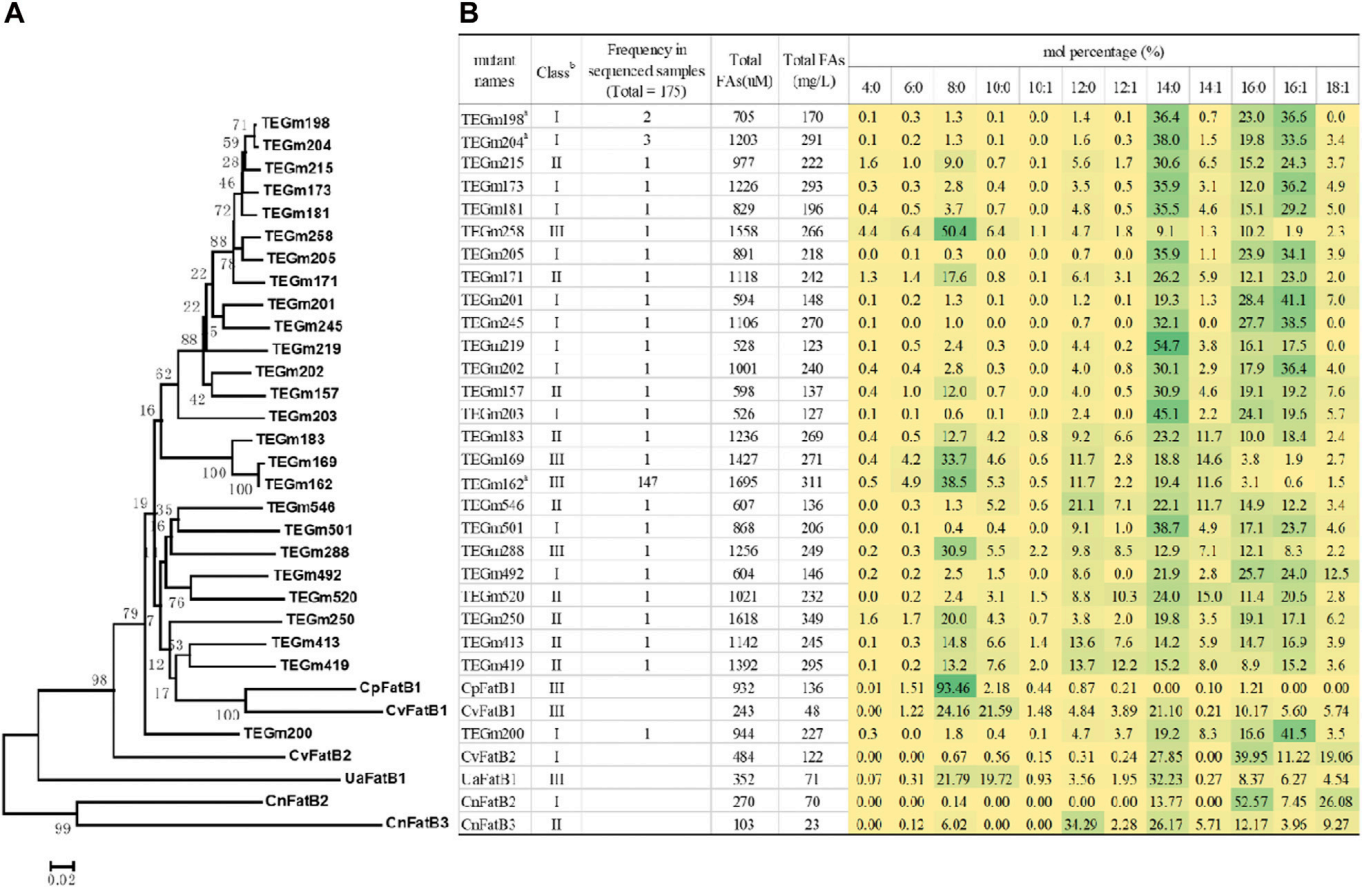
Fatty acid titers and fatty acid specificity of evolved acyl-ACP TE variants. **(A)** Dendrogram representation of sequence similarities among acyl-ACP TE variants. The dendrogram was inferred using the Minimum Evolution method (Rzhetsky and Nei, 1993). The bootstrap consensus tree (bootstrap value identified at each node), which was inferred from 250 replicates, represents the evolutionary history of each acyl-ACP TE. **(B)** Fatty acid profiles of 26 unique acyl-ACP TE variants generated in this study and compared to the six parental acyl-ACP TEs used to constrain the directed evolution strategy. The intensity of the green shading of each cell is proportional to the mol% of each fatty acid. ^a^ Among the 175 acyl-ACP TE variants recovered in this study, the TEGm2198, TEGm204 and TEGm162 variants recurred 2, 3, and 147 times, respectively. ^b^ Acyl-ACP TEs can be classified into three groups based on their substrate specificity: Class I enzymes primarily hydrolyze acyl-ACPs of 14- and 16-carbon acyl-chains, Class II enzymes prefer 8- to 16-carbon acyl-chains, and Class III enzymes have a preference for 8-carbon acyl-chains.

Hierarchical clustering analysis of these variant sequences identify a majority clade that is most similar to two of the parental sequences, CvFatB1 and CpFatB1 (Figure 3A). Within this clade, variants TEGm413 and TEGm419 are closest in sequence to the CpFatB1 and CvFatB1 parents, and these four proteins share −64% amino acid identity, and they yield fatty acid titers that range between approximately 240 μM and approximately 1390 µM (Figure 3B).

### 3.4 The substrate specificities of acyl-ACP TE variants

In addition to generating differences in *in vivo* fatty acid titer, the six parental acyl-ACP TEs that were used to guide this directed evolution strategy also displayed differences in acyl-chain length substrate specificity. This variation provided an added opportunity to explore the relationship between the structure and substrate specificity attributes of acyl-ACP TEs. Therefore, we evaluated how substrate specificity evolved in the acyl-ACP TE variants that were selected for inducing enhanced *in vivo* fatty acid titers.

Figure 3B shows the fatty acid profiles produced by the 26 evolved acyl-ACP TE variants as compared to the six parental acyl-ACP TEs. Prior characterizations of the six parental acyl-ACP TEs, in the context of 31 naturally occurring diverse acyl-ACP TEs from plant and microbial sources, had categorized these parental enzymes into three classes, Class I, Class II and Class III (Jing et al., 2011). CvFatB2 and CnFatB2 are Class I enzymes that primarily hydrolyze acyl-ACPs of 14- and 16-carbon fatty acyl-chains, CnFatB3 is a Class II enzyme that prefers acyl-ACPs of 8- to 16-carbon acyl-chains, and CpFatB1, CvFatB1, and UaFatB1 are Class III enzymes that have a preference for 8-carbon acyl-chains (Jing et al., 2011). The 26 acyl-ACP TE variants generated by the directed evolution study distributed somewhat unevenly among these three functional classes, with a preference for Class I and Class II enzymes (13 and 9 variants, respectively), and only four variants (TEGm162, TEGm169, TEGm258, and TEGm288) belonged to Class III acyl-ACP TEs. Although these 26 variant acyl-ACP TEs are classifiable among these three categories, an analysis of variance (ANOVA) demonstrates that these substrate specificity classifications do not correlate with the observed variations in the *in vivo* fatty acid titer generated by the *E. coli* host (*p*-value >0.05). Therefore, structural features that determine substrate specificity are independent of the structural features that determine catalytic efficiency of these enzymes.

### 3.5 Machine learning model reveals structural constraints to substrate specificity

**FIGURE 4:**
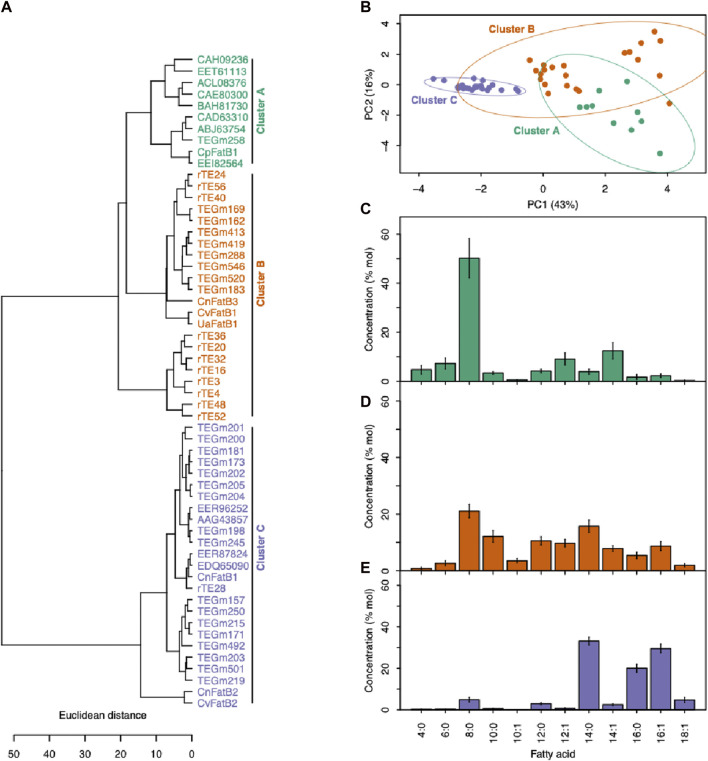
Categorizing acyl-ACP TEs based on fatty acid profiles. **(A)** Enzyme cluster membership was determined by hierarchical clustering of fatty acid profiles produced when each acyl-ACP TE was expressed in *E. coli*. **(B)** The PCA plot based on the fatty acid profiles produced when each acyl-ACP TE was expressed in *E. coli*. PC1 and PC2 together explain 59% of the data variation, and segregate the 57 enzymes into three clusters demonstrated by 95% confidence ellipses. **(C)** The fatty acid profiles produced by the acyl-ACP TEs that belong to Cluster A (as defined in panels **(A)** and **(B)**). **(D)** The fatty acid profiles produced by the acyl-ACP TEs that belong to Cluster B (as defined in panels **(A)** and **(B)**). **(E)** The fatty acid profiles produced by the acyl-ACP TEs that belong to Cluster C (as defined in panels **(A)** and **(B)**).

Because acyl-ACP TE classification based solely on sequence similarity and diversity does not fully predict the fatty acid titers generated by these enzymes, we adopted an alternative classification strategy based on the fatty acid product profiles. Thus, in addition to clustering the variant acyl-ACP TEs relative to their sequence similarity (Figure 3A), clustering was performed based on the fatty acid profiles produced when variant enzymes were expressed *in vivo* to evaluate their substrate specificity (Figure 4A). These analyses not only evaluated the acyl-ACP TEs generated by the current *in vitro* directed evolution study, but also included previously characterized natural variants of acyl-ACP TE isolated from a wide variety of different phylogenetic clades (Jing et al., 2011; Jing et al., 2018a). Thus, collectively 57 acyl-ACP TE variants were analyzed, 26 being products of *in vitro* directed evolution selection, and 31 being products of natural evolution selection.

Hierarchical clustering that minimized within-cluster variance in substrate specificity separated the 57 acyl-ACP TE variants into three distinct clusters (Clusters A-C) (Figure 4A). A similar segregation pattern occurs upon principal component analysis (PCA) of these data (Figure 4B), and in combination the two primary principal components (PC1 and PC2) explain nearly 60% of the variation in the substrate specificity among these variants. PC1, which accounts for 43% of the variation in the fatty acid profiles, primarily separates Cluster C-enzymes from Clusters A and B, while PC2 explains 16% of the variation, and separates Cluster A from Cluster B and Cluster C (Figure 4B). This tripartite classification of the variants reflects the prior classification of naturally occurring acyl-ACP TEs variants (Jing et al., 2011), which identified three classes of acyl-ACP TEs, with preferences for C14/C16 (Class I), C8 (Class III) or broad range chain-length (Class II) acyl-ACP TE substrates. Similarly in this study, Cluster A and Cluster C enzymes exhibit preferences for C8 and C14/C16 acyl-ACPs, respectively, whereas Cluster B enzymes have broader substrate specificities, enabling hydrolysis of C8 to C16 acyl-ACPs (Figures 4C–E).

Manual comparisons of the recovered acyl-ACP TE sequence variants and their substrate specificities can provide constraints on the relationship between primary structure and substrate specificity of these enzymes. For example, by comparing the acyl-ACP TE sequence variants that are sorted into the same sequence-based hierarchical cluster, but are separated into different functional classes based on substrate specificities (i.e., Classes A-C; Figure 4A), one can heuristically identify those polymorphic residues that contribute to altered substrate specificity. We instead developed a systematic computational machine learning random forest classification model that improves on this manual strategy, and quantitatively assesses the importance of each polymorphic amino acid residue in determining the substrate specificity of the acyl-ACP TE variants.

**FIGURE 5:**
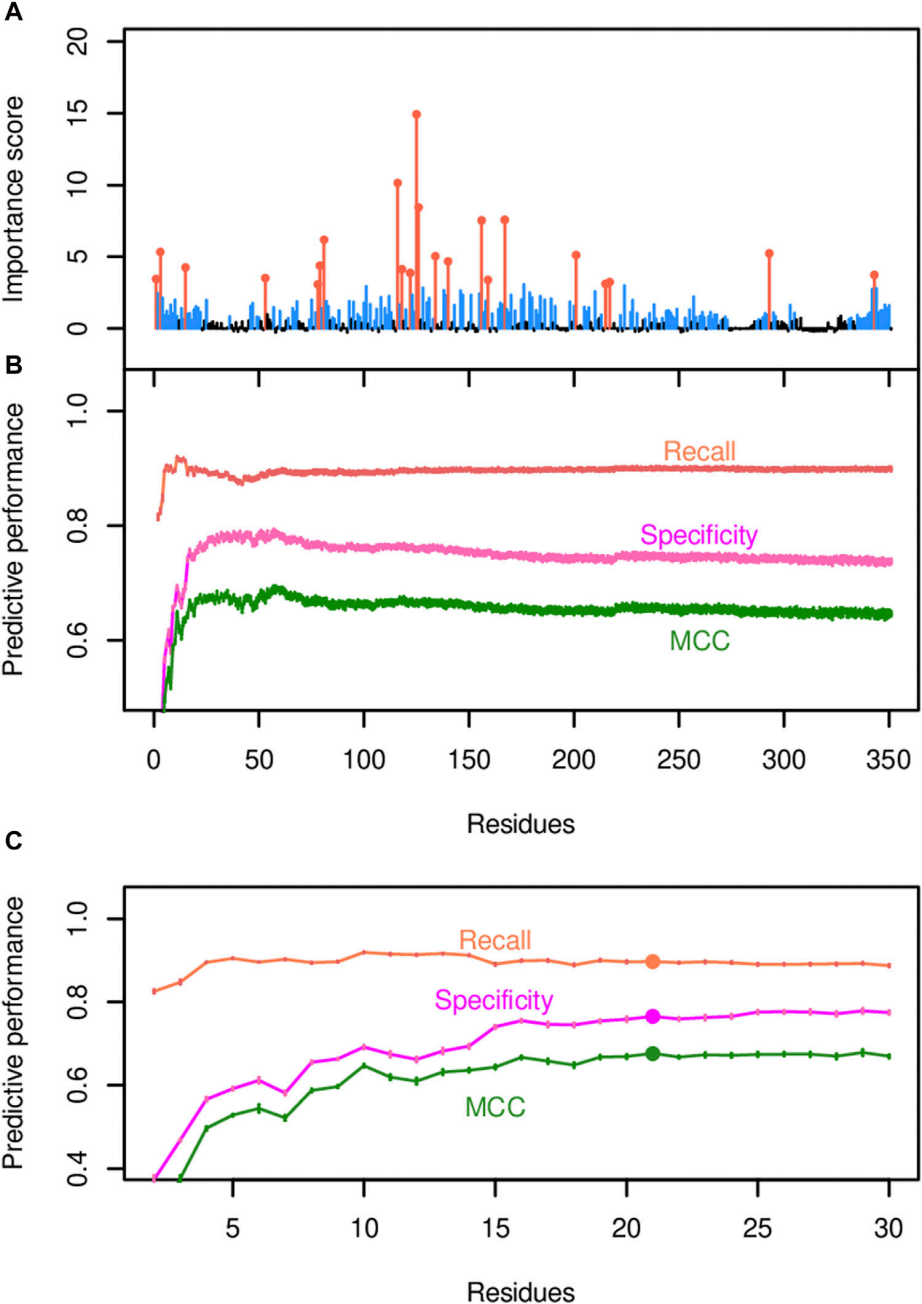
Identification of residue positions predicted to govern acyl-ACP TE substrate specificity. **(A)** The importance scores for each residue position were generated by the random forest model that uses all 350 positions and one random variable as the predictors. The most impactful positions that determine the substrate specificity of the enzyme (orange-colored data points) were identified via Incremental Feature Selection (IFS) and have q-values <0.001. Non-significant positions are in black. **(B)** IFS selects the most important predictor set by evaluating the predictive performance of the associated model, as demonstrated by recall, specificity, and MCC. **(C)** A zoom-in view of the predictive performance evaluated by IFS. MCC hits the plateau when the top 22 residue positions (highlighted by filled circles) are included in the model.

The random forest classification strategy utilized both binarized substrate specificity data and amino acid sequence data as described in the Methods. Substrate specificity was binarized according to the fatty acid profiles produced by each variant enzyme in *E. coli*, and two acyl-ACP TEs were defined as sharing substrate specificity if they were members of the same Cluster (A, B or C) (Figure 4A). In juxtaposition, two acyl-ACP TEs that had membership in separate Clusters were deemed as having different substrate specificities. After transforming and encoding the data, a random forest classifier was trained with all encoded data, and the mean feature importance scores for the 350 amino acid positions were calculated based on ten iterations of the model (Figure 5A and Supplementary Table S4A). These analyses quantified the importance of individual residues in determining substrate specificity of each acyl-ACP TE variant. A total of 174 residue positions with importance scores ranging from approximately 0.5 to approximately 15, had a statistically significant impact on substrate specificity (i.e., corrected *p*-values <0.001; Supplementary Table S4A), and these are blue-highlighted in Figure 5A.

This list of residues was refined by a two-step approach. Initially, an incremental feature selection (IFS) approach was used that built a series of random forest models, in which each model added an additional residue to the evaluation process. The random forest classifier that included the 59 residue positions with the highest importance scores as the predictors exhibited optimal predictive performance, with a recall (i.e., true positive rate) of 92%, a specificity (true negative rate) of 95%, and a MCC of 0.87, which measures the correlation between the predicted and actual outcomes (Figure 5B; Supplementary Table S4A). Next, the list of 59 residues was further prioritized by pairwise comparisons of MCC scores using Student’s t-tests between every pair of adjacent models (i.e., the model that included one additional residue position versus the previous model that did not include that residue) until all 59 residues were examined (Figures 5B,C). The final model that contained the top 22 residue positions (orange-highlighted in Figure 5A) reached the statistical plateau of MCC (q-value >0.05; Supplementary Table S4A), and thus these 22 residues were considered as most impactful in determining the substrate specificity of the enzyme.

**FIGURE 6:**
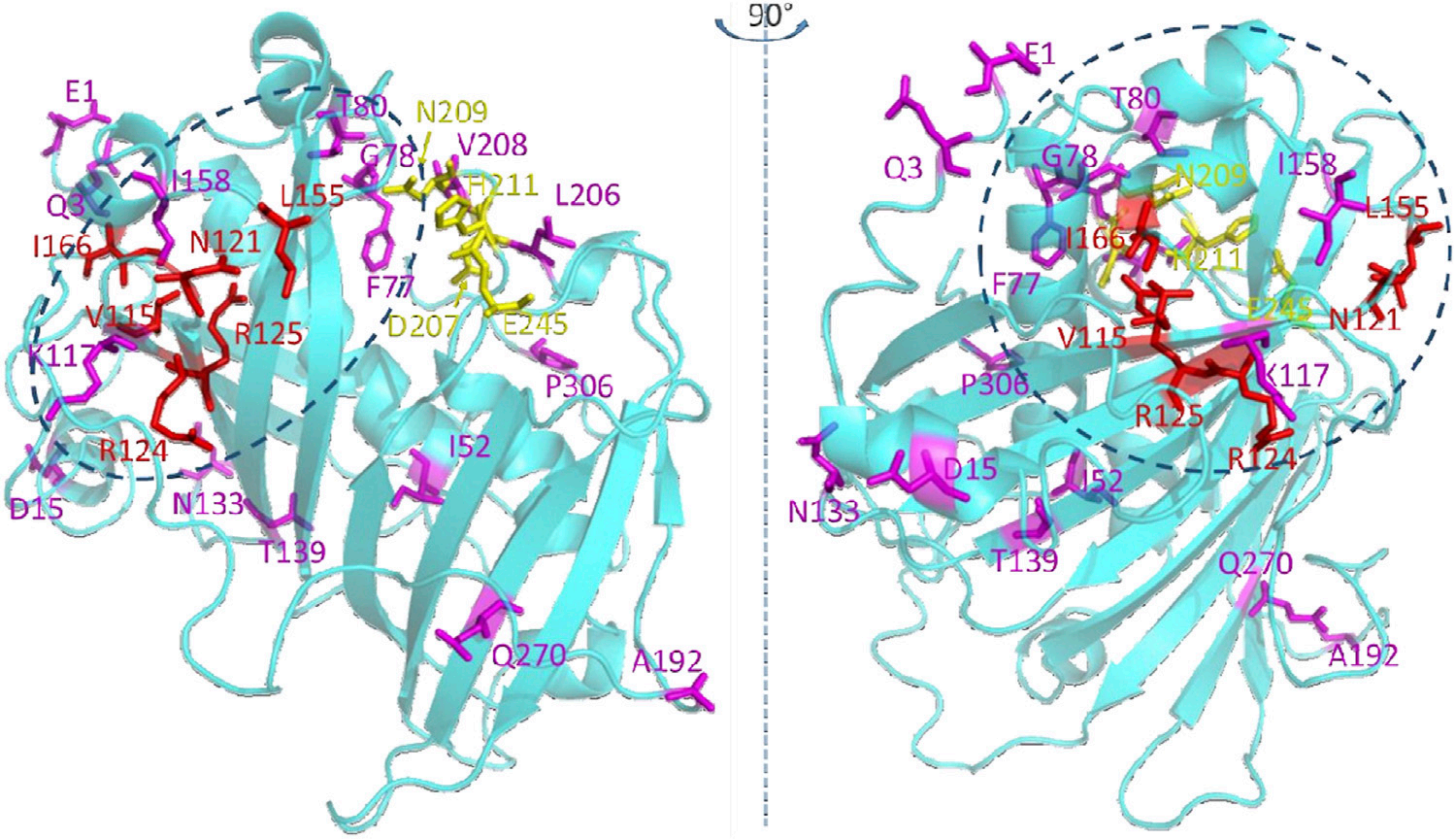
Residues that are significant in determining the substrate specificity of acyl-ACP TE. The top twenty-two residues selected by the random forest classifier (Figure 5) are shown as stick models. Red colored residues have previously been experimentally verified to affect substrate specificity (Jing et al., 2018a; Jing et al., 2018b). Catalytic residues are shown in yellow (Mayer and Shanklin, 2005; Serrano-Vega et al., 2005; Feng et al., 2017; Jing et al., 2018a). The dotted ovals indicate the structural region where the substrate binding pocket is located.

Mapping these 22 residues onto a predicted three dimensional structure of CvFatB2 indicates that the majority of these residues (17 of 22) are located in the N-terminal hot-dog domain structure (Figure 6). The other five residues are in the C-terminal hot-dog domain structure, among which four are adjacent to the catalytic residues we identified in a previous study (Jing et al., 2018b).

## 4 Discussion

A number of microbial chassis have been proposed for the conversion of sugar feedstocks to fatty acids, including bacteria and yeast (Lennen and Pfleger, 2012; Leber and Da Silva, 2014; Cho et al., 2020). One of the key biocatalysts that has been the focus of these conversion processes is acyl-ACP TE, the enzyme that terminates the process of fatty acid biosynthesis by hydrolyzing the fatty acid product from the FAS enzyme (Swarbrick et al., 2020). Beginning with the pioneering work conducted at the biotechnology company, Calgene Inc. (Voelker et al., 1992), a bioengineering strategy has been developed to use diverse acyl-ACP TEs (Jing et al., 2011) to intercept the FAS system, and thereby generate new products from this metabolic process (e.g., Hernández Lozada et al., 2018; Cahoon and Li-Beisson, 2020). This bioengineering strategy releases the FAS system from the “normal” regulatory circuit that controls the fatty acid productivity of the chassis, resulting in the over-production of fatty acids. Because of this utility of acyl-ACP TEs, the ThYme database (Cantu et al., 2011) has compiled nearly 40,000 sequences of such enzymes from plant and bacterial sources (i.e., Families TE14 to TE19, and TE30) (Caswell et al., 2022).

In this study we developed a facile, directed evolution strategy to generate novel acyl-ACP TEs for the purpose of enhancing the fatty acid titers generated by *E. coli*. The effectiveness of this strategy is exemplified by the fact that just a single round of directed evolution, screening only approximately 30,000 variants, yielded 26 distinct acyl-ACP TEs, with up to 10-fold increase in fatty acid titer, as compared to the initial parental enzymes that constrained the *in vitro* directed evolution strategy. Thus, the resultant bacterial strains that harbor these enhanced biocatalysts improved the efficiency of the conversion of glucose to fatty acids.

While the improvement of enzymatic activity is an important target for using acyl-ACP TE enzymes to overproduce fatty acids, this enzyme’s substrate specificity is another significant attribute that can be bioengineered because it determines the chain length of fatty acids produced by a microbial chassis. This latter trait is important in determining the “performance” of the fatty acid products in the application arena, which is a prominent determinant for the market niche of these fatty acid products.

We had previously determined substrate specificity of a small subset of these acyl-ACP TEs cataloged in the ThYme database (Cantu et al., 2011), which enabled the classification of acyl-ACP TEs into three categories (Class I, II, and III) (Jing et al., 2011). More recently, using such substrate specificity data and primary sequences for 115 experimentally characterized acyl-ACP TEs gleamed from the academic (e.g., (Jing et al., 2011; Jing et al., 2018a), and patent literature, a machine learning discriminatory strategy (i.e., EnZymClass) was developed (Banerjee et al., 2022). EnZymClass was used to predict the substrate specificity categorization of 617 acyl-ACP TEs from primary sequences, considering common sequence motifs, physicochemical properities, and evolutionary history. This categorization was validated by the identification of two novel Class I acyl-ACP TEs (Banerjee et al., 2022). However, the underlying sequence features dictating the substrate specificity of these enzymes remains elusive.

Although the directed evolution strategy implemented in the current study was designed to increase fatty acid titers generated by the microbial chassis, the recovered enzymes also diversified the fatty acids produced by the microbial chassis. These data therefore provided an opportunity to explore the relationship between acyl-ACP TE sequence and substrate specificity. Because the directed evolution strategy changed the parent enzymes’ catalytic capabilities by mutating individual amino acids, we aimed at quantifying the importance of each residue relative to these changes. Hence, we integrated data from the 26 variant enzymes generated within this study with data previously generated from 31 naturally occurring variant enzymes isolated from plants and bacteria (Jing et al., 2011). Using fatty acid profile data combined from these 57 variant enzymes, a random forest classification algorithm systematically assessed the impact of each acyl-ACP TE residue on determining the substrate specificity of the enzyme. Such random forest strategies have proven useful in quantitatively modeling relationships between protein sequence and different protein functionalities, including protein folding and crystallizability (Jahandideh and Mahdavi, 2012; Jo and Cheng, 2014; Bonetta and Valentino, 2020). Collectively, these analyses assigned an importance score to each residue for its ability to affect a change in substrate specificity of the enzyme. Twenty two of the most significant contributors in determining the substrate specificity of the enzyme were identified. Six of these residues had previously been identified in the CvFatB2 enzyme (i.e., V115, N121, R124, R125, L155, and I166 of the CvFatB2 sequence) via a domain shuffling strategy and confirmed by site-directed mutagenesis studies as being critical in determining substrate specificity (Jing et al., 2018b).

The majority of highest scoring residues (17 out of 22 residues) reside within the N-terminal hot-dog structure of the predicted tertiary structure model of CvFatB2a (Figure 6). This location is consistent with our prior postulate (Jing et al., 2018a) that the substrate specificity of this enzyme is determined by the chemophysical nature of the substrate binding pocket located in the N-terminal hot-dog domain of these enzymes; the substrate binding pocket being formed between the central α-helix and the antiparallel β-sheets in the N-terminal hotdog domain (Jing et al., 2018b). Specifically, the active site residues of acyl-ACP TE are located on the C-terminal hot-dog structure at the interface between the N-terminal and C-terminal hot-dog structures. While four of these residues (i.e., V115, N121, R124, and R15) that are located on the antiparallel β-sheets had previously been identified as being important in determining substrate specificity (Jing et al., 2018a), five additional amino acids (i.e., I52, L59, A63, L64, and V67) located on the central α-helix of the N-terminal hot-dog structure have been identified by the current machine learning strategy.

By localizing the three dimensional positions of the 22 most significant residues identified by machine learning (Figure 6), we hypothesize that the residues on the central α-helix and the antiparallel β-sheets of acyl-ACP TE determine the substrate specificity of this enzyme by defining the size and chemophysical properties of the substrate binding pocket. Other residues identified (i.e., V26, D29, N74, Y84, D87, N100, and A192) are located at the opening of the active site cleft near the surface of the acyl-ACP TE protein, and they may affect the substrate specificity by modulating the interactions between the enzyme and the ACP moiety of the substrate. Indeed, we had previously identified residues at the surface of the acyl-ACP TE enzyme that are important for protein-protein interaction and thus affect catalytic efficiency and substrate specificity of acyl-ACP TE (Jing et al., 2018b).

Collectively, the findings presented herein provide an experimentally-based computational model that pinpoints amino acid residues that potentially determine the substrate specificity of acyl-ACP TEs. This study demonstrates the feasibility of combining an *in vitro* directed evolution approach with downstream computational analysis to identify key structural features (i.e., amino acid residues) of an enzyme that can be targeted in a rational redesign strategy to further enhance the titer and specificity of a microbial fatty acid biofactory.

In this study, we demonstrate an integrated directed evolution-machine learning strategy that has been used to understand the structural features of the protein that contribute to increasing the catalytic efficiency of acyl-ACP TE and further expand the knowledge on the structural determinants of the substrate specificity of this enzyme. Such a strategy enables the alteration of two attributes of this important biocatalyst and its utilization to build an efficient biosynthetic pathway for producing desired fatty acids as feedstocks for biorenewable chemicals.

## Data Availability

The datasets presented in this study can be found in online repositories. The names of the repository/repositories and accession number(s) can be found in the article/Supplementary Material.
