# Supplementary material for: Machine learning model of the catalytic efficiency and substrate specificity of acyl-ACP thioesterase variants generated from natural and in vitro directed evolution
Source: Front Bioeng Biotechnol. 2024 Apr 11;12:1379121. doi: 10.3389/fbioe.2024.1379121 (PMC11043601; doi:10.3389/fbioe.2024.1379121)
Supplement: Supplementary file 1 [file DataSheet1.PDF]

Supplementary Material

Supplementary Material

1 Supplementary Figures and Tables

1.1 Supplementary Figures

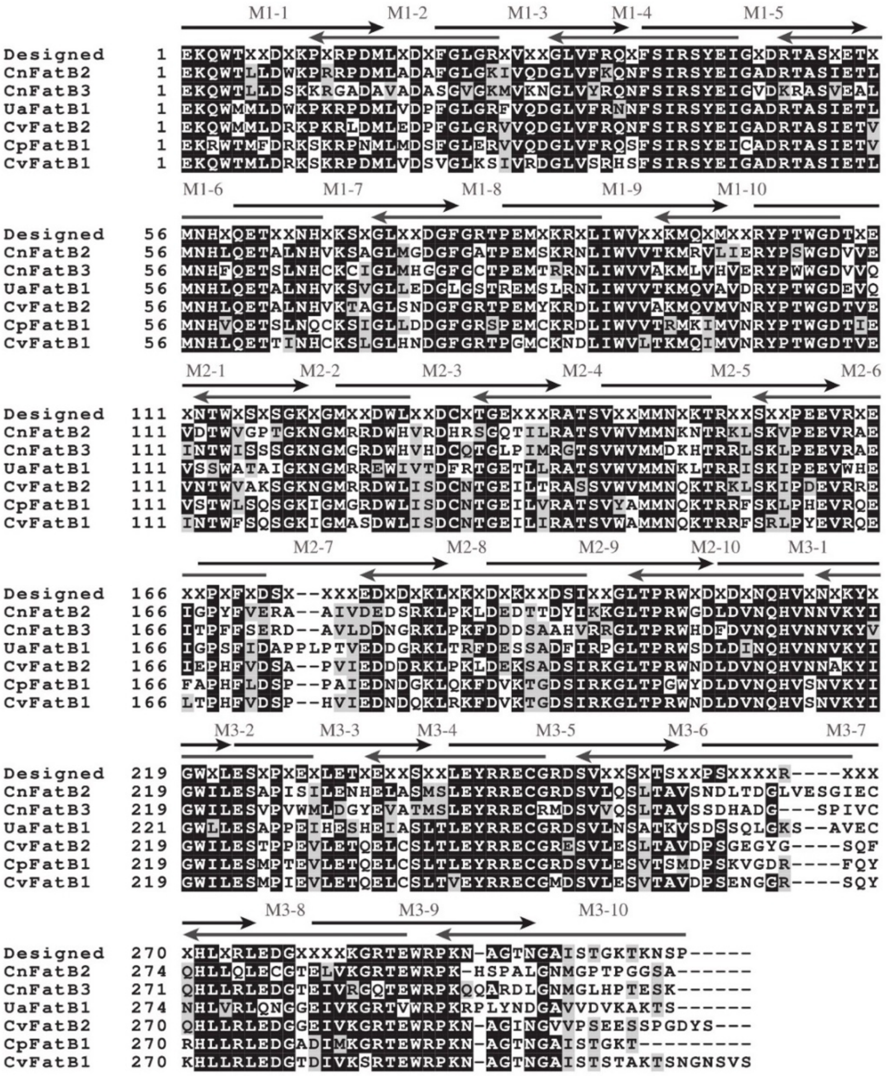

Supplementary Figure 1. Sequence alignment of the six parental acyl-ACP TEs and the Designed acyl-ACP TE protein. The multiple sequence alignment was constructed by using ClustalW2 (http://www.ebi.ac.uk/Tools/msa/clustalw2/). The “X” residues in the Designed sequence identify the amino acids that were potentially varied in the reassembled acyl-ACP TE variants. Arrows (labeled as M1-1 to M1-10, M2-1 to M2-10, and M3-1 to M3-10) indicate the location of the primers that were used to reassemble the Designed acyl-ACP TE sequences. Conserved residues are shown with white font on a black background, and white font residues on a grey background identify residues that are functionally similar.

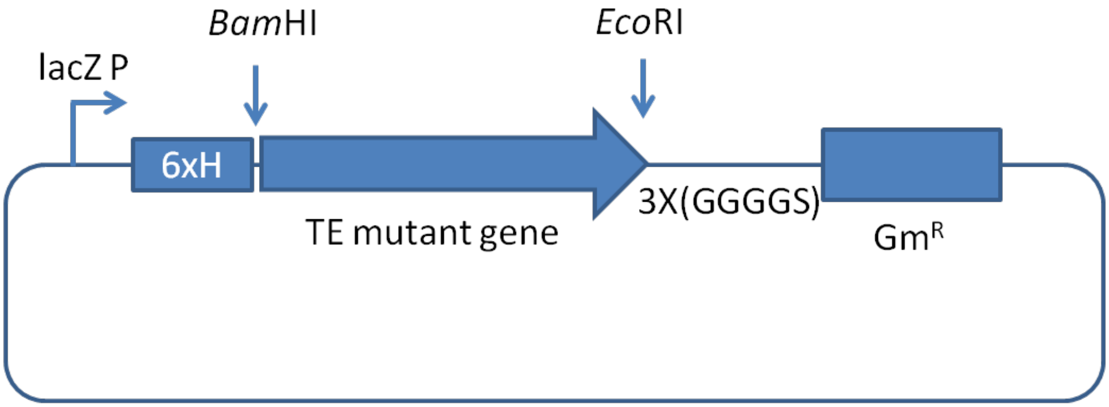

Supplementary Figure 2. Schematic map of the pUCHisGm expression vector used to construct the acyl-ACP TE variant library. 6xH, hexa-histidine sequence tag; lacZ P, promoter of *lacZ* gene; 3x(GGGGS), [Gly-Gly-Gly-Gly-Ser]$_3$ linker sequence; Gm^R, gentamicin-resistance gene, fused in-frame with the acyl-ACP TE sequence.

Supplementary Figure 3. Multiple sequence alignment of acyl-ACP TE variants characterized in this study.

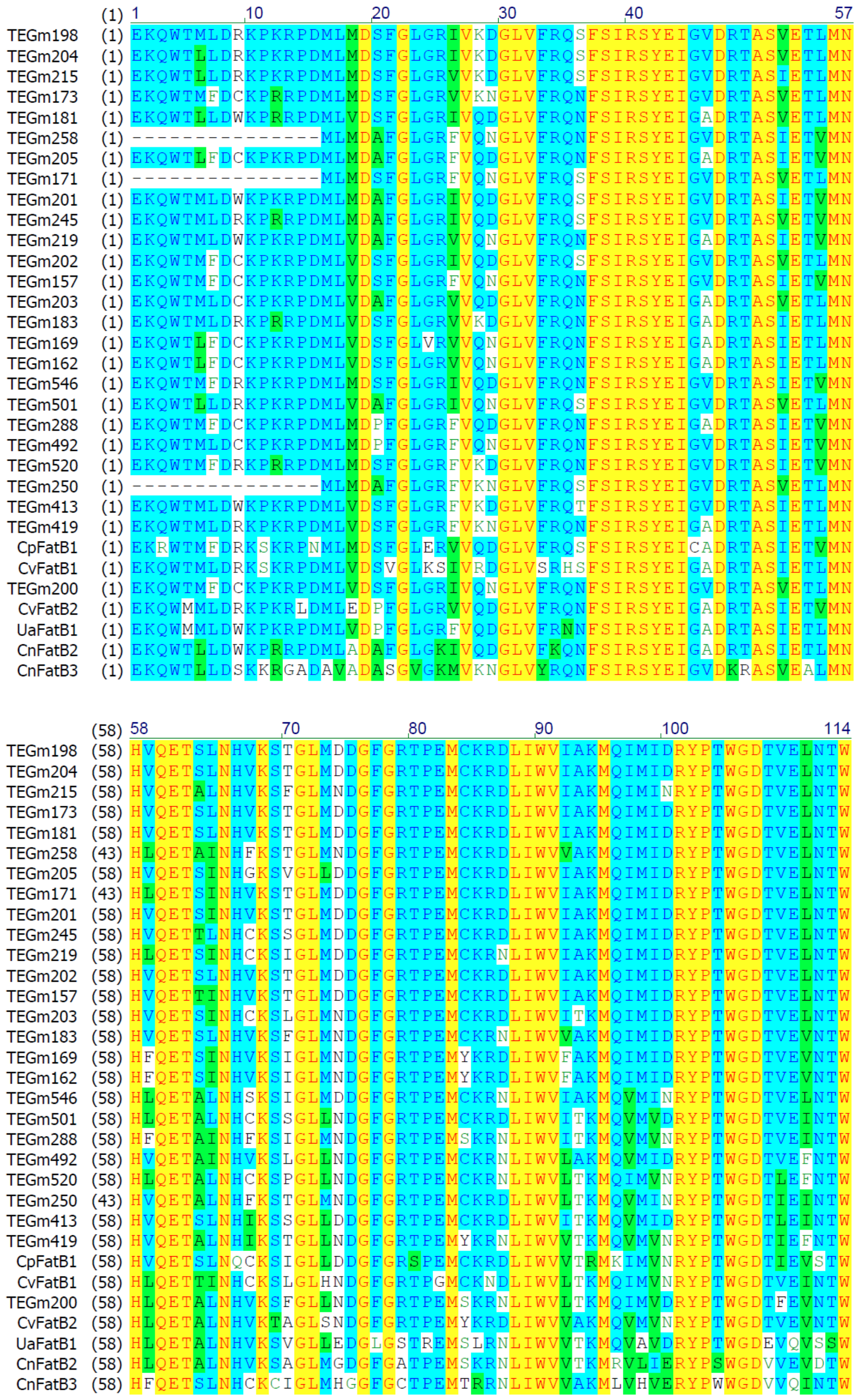

Supplementary Figure 3 (continued)

```
        (115) 115 120           130           140           150           160          171
TEGm198 (115) ISESGKNGMRRDWLICDCNTGEILVRATSVWVMMNEKTRKLSKFPEEVRQEVAPHFI
TEGm204 (115) ISESGKNGMRRDWLICDCNTGEILVRATSVWVMMNEKTRKLSKFPEEVRQEVAPHFI
TEGm215 (115) ISESGKNGMRRDWLICDCNTGEILVRATSVWVMMNEKTRKLSKFPEEVRQEVAPHFI
TEGm173 (115) ISESGKNGMRRDWLICDCNTGEILVRATSVWVMMNEKTRKLSKFPEEVRQEVAPHFI
TEGm181 (115) ISESGKNGMRRDWLICDCNTGEILVRATSVWVMMNEKTRKLSKFPEEVRQEVAPHFI
TEGm258 (100) ISESGKNGMRRDWLICDCNTGEILVRATSVWVMMNEKTRKLSKFPEEVRQEVAPHFI
TEGm205 (115) ISESGKNGMRRDWLICDCNTGEILVRATSVWVMMNEKTRKLSKFPEEVRQEVAPHFI
TEGm171 (100) ISESGKNGMRRDWLICDCNTGEILVRATSVWVMMNEKTRKLSKFPEEVRQEVAPHFI
TEGm201 (115) ISESGKNGMRRDWLICDCNTGEILVRATSVWVMMNEKTRKLSKFPEEVRQEVAPHFI
TEGm245 (115) ISESGKNGMRRDWLICDCNTGEILVRATSVWVMMNEKTRKLSKFPEEVRQEVAPHFI
TEGm219 (115) ISESGKNGMRRDWLICDCNTGEILVRATSVWVMMNEKTRKLSKFPEEVRQEVAPHFI
TEGm202 (115) ISESGKNGMRRDWLICDCNTGEILVRATSVWVMMNEKTRKLSKFPEEVRQEVAPHFI
TEGm157 (115) ISESGKNGMRRDWLICDCNTGEILVRATSVWVMMNEKTRKLSKFPEEVRQEVAPHFI
TEGm203 (115) ISESGKNGMRRDWLICDCNTGETIIRATSVCVMMNQKTRKLSRLPEEVRQEIGPHFL
TEGm183 (115) FSKSGKNGMGRDWLIRDCQTGETIIRATSVWVMMNQKTRKLSKIPEEVRRELAPYFI
TEGm169 (115) FSKSGKNGMGRDWLIRDCQTGETIIRATSVWVMMNQKTRKLSKIPEEVRRELAPYFI
TEGm162 (115) FSKSGKNGMGRDWLIRDCQTGETIIRATSVWVMMNQKTRKLSKIPEEVRRELAPYFI
TEGm546 (115) ISKSGKIGMGSDWLVCDCKTGETIIRATSVYVMMNEKTRRFSRFPEEVRREIAPHFF
TEGm501 (115) ISKSGKIGMASDWLVSDCNTGETIVRATSVYAMMNKKTRRLSKIPEEVRREVAPHFI
TEGm288 (115) FSKSGKIGMASDWLITDCKTGEIIVRATSVCVMMNKKTRRLSKIPEEVRQEVGPYFF
TEGm492 (115) FSKSGKNGMGRDWLVSDCHTGEIIMRATSVYVMMNKKTRKLSRIPEEVRREVGPYFF
TEGm520 (115) FSQSGKNGMGSDWLVTDCHTGETIMRATSVCVMMNQKTRKISKIPEEVRREIAPYFL
TEGm250 (100) VSQSGKIGMGRDWLIRDCNTGETLIRATSVWAMMNEKTRRISRFPEEVRREVGPYFF
TEGm413 (115) FSQSGKNGMASDWLITDCKTGEIIVRATSVYVMMNEKTRKLSRLPEEVRREFGPHFI
TEGm419 (115) FSKSGKNGMASDWLICDCNTGEILIRATSVYAMMNEKTRRFSRLPEEVRREFAPYFI
CpFatB1 (115) LSQSGKIGMGRDWLISDCNTGEILVRATSVYAMMNQKTRRFSKLPHEVRQEFAPHFL
CvFatB1 (115) FSQSGKIGMASDWLISDCNTGEILIRATSVWAMMNQKTRRFSRLPYEVRQELTPHFV
TEGm200 (115) FSKSGKNGMRRDWLIPDCQTGEIIVRATSVWVMMNQKTRKLSKIPEEVRREIAPYFF
CvFatB2 (115) VAKSGKNGMRRDWLISDCNTGEILTRASSVWVMMNQKTRKLSKIPDEVRREIEPHFV
UaFatB1 (115) ATAIGKNGMRREWIVTDFRTGETLLRATSVWVMMNKLTRRISKIPEEVWHEIGPSFI
CnFatB2 (115) VGPTGKNGMRRDWHVRDHRSGQTILRATSVWVMMNKNTRKLSKVPEEVRAEIGPYFV
CnFatB3 (115) ISSSGKNGMGRDWHVHDCQTGLPIMRGTSVWVMMDKHTRRLSKLPEEVRAEITPFFS

        (172) 172    180           190           200           210                     228
TEGm198 (172) DS--APVLEDDDRKLRKIDVKSADSIRRGLTPRWNDIDINQHVNNVKYFGWFLESVP
TEGm204 (172) DS--APVLEDDDRKLRKIDVKSADSIRRGLTPRWNDIDINQHVNNVKYFGWFLESVP
TEGm215 (172) DS--APVLEDDDRKLRKIDVKSADSIRRGLTPRWNDIDINQHVNNVKYFGWFLESVP
TEGm173 (172) DS--APVLEDDDRKLRKIDVKSADSIRRGLTPRWNDIDINQHVNNVKYFGWFLESVP
TEGm181 (172) DS--APVLEDDDRKLRKIDVKSADSIRRGLTPRWNDIDINQHVNNVKYFGWFLESVP
TEGm258 (157) DS--APVLEDDDRKLRKIDVKSADSIRRGLTPRWNDIDINQHVNNVKYFGWFLESVP
TEGm205 (172) DS--APVLEDDDRKLRKIDVKSADSIRRGLTPRWNDIDINQHVNNVKYFGWFLESVP
TEGm171 (157) DS--APVLEDDDRKLRKIDVKSADSIRRGLTPRWNDIDINQHVNNVKYFGWFLESVP
TEGm201 (172) DS--APVLEDDDRKLRKIDVKSADSIRRGLTPRWNDIDINQHVSNVKYFGWILESMP
TEGm245 (172) DS--APVLEDDDRKLRKIDVKSADSIRRGLTPRWNDIDINQHVNNVKYVGWVLESVP
TEGm219 (172) DS--APVLEDDDRKLRKIDVKSADSIRRGLTPRWNDIDLNQHVSNVKYVGWFLESMP
TEGm202 (172) DS--APVLEDDDRKLRKIDVKSADSIRRGLTPRWNDIDINQHVNNAKYLGWVLESVP
TEGm157 (172) DS--APVFEDNDRKLRKIDVKSGDSIKRGLTPRWNDIDLNQHVNNAKYLGWILESVP
TEGm203 (172) DS--APAIEDDDRKLRKFDVKSADSIKKGLTPRWNDFDINQHVNNAKYIGWLLESVP
TEGm183 (172) DS--AAVLEDNDRKLQKIDVKSGDSIKRGLTPRWNDVDVNQHVNNAKYLGWVLESVP
TEGm169 (172) DS--AAVLEDNDRKLQKIDVKSGDSIKRGLTPRWNDVDVNQHVNNAKYLGWVLESVP
TEGm162 (172) DS--AAVLEDNDRKLQKIDVKSGDSIKRGLTPRWNDVDVNQHVNNAKYLGWVLESVP
TEGm546 (172) DS--AAVLEDDDRKLQKLDEKTGDSIRRGLTPRWNDIDLNQHVNNAKYIGWILESMP
TEGm501 (172) DS--AAVVEDNDGKLQKVDEKSGDSIKKGLTPRWNDIDVNQHVNNAKYIGWILESMP
TEGm288 (172) DS--AAVLEDDDRKLQKLDEKSGDSIRRGLTPRWSDLDINQHVNNVKYIGWLLESVP
TEGm492 (172) DS--AAAIEDDDGKLQKFDVKTGDSIRKGLTPRWNDIDFNQHVNNVKYIGWILESMP
TEGm520 (172) DS--AAAVEDDDGKLPKLDEKTADSIRKGLTPRWNDIDLNQHVNNAKYLGWILESVP
TEGm250 (157) DS--APVFEDNDGKLPKIDVKTADSIKRGLTPRWNDFDLNQHVNNVKYFGWILESMP
TEGm413 (172) DS--PPVLKDDDRKLRKIDVKSGDSIKRGLTPRWNDFDVNQHVNNAKYIGWLLESMP
TEGm419 (172) DS--PAALEDDDRKLRKLDVKTGDSIRRGLTPRWNDLDLNQHVNNAKYFGWLLESVP
CpFatB1 (172) DS--PPAIEDNDGKLQKFDVKTGDSIRKGLTPGWYDLDVNQHVSNVKYIGWILESMP
CvFatB1 (172) DS--PHVIEDNDQKLRKFDVKTGDSIRKGLTPRWNDLDVNQHVSNVKYIGWILESMP
TEGm200 (172) DS--PPVIEDDDRKLPKFDVKTADSIRRGLTPRWNDFDINQHVNNVKYFGWILESVP
CvFatB2 (172) DS--APVIEDDDRKLPKLDEKSADSIRKGLTPRWNDLDVNQHVNNAKYIGWILESTP
UaFatB1 (172) DAPPLPTVEDDGRKLTRFDESSADFIRPGLTPRWSDLDINQHVNNVKYIGWLLESAP
CnFatB2 (172) ER--AAIVDEDSRKLPKLDEDTTDYIKKGLTPRWGDLDVNQHVNNVKYIGWILESAP
CnFatB3 (172) ER--DAVLDDNGRKLPKFDDDSAAHVRRGLTPRWHDFDVNQHVNNVKYVGWILESVP
```

Supplementary Figure 3 (continued)

```
        (229) 229       240       250       260       270           285
TEGm198 (227) IEILETHEVCSLSLEYRRECGRDSVLQSLTSVDPSKEGDR----FEYQHLLRLEDGT
TEGm204 (227) IEILETHEVCSLSLEYRRECGRDSVLQSLTSVDPSKEGDR----FEYQHLLRLEDGT
TEGm215 (227) IEILETHEVCSLSLEYRRECGRDSVLQSLTSVDPSKEGDR----FEYQHLLRLEDGT
TEGm173 (227) IEILETHEVCSLSLEYRRECGRDSVLQSLTSVDPSKEGDR----FEYQHLLRLEDGT
TEGm181 (227) IEILETHEVCSLSLEYRRECGRDSVLQSLTSVDPSKEGDR----FEYQHLLRLEDGT
TEGm258 (212) IEILETHEVCSLSLEYRRECGRDSVLQSLTSVDPSKEGDR----FEYQHLLRLEDGT
TEGm205 (227) IEILETHEVCSLSLEYRRECGRDSVLQSLTSVDPSKEGDR----FEYQHLLRLEDGT
TEGm171 (212) IEILETHEVCSLSLEYRRECGRDSVLQSLTSVDPSKVDDR----VQYQHLVRLEDGT
TEGm201 (227) TEILETHEICSMTLEYRRECGRDSVLESVTSVDPSEEDGR----FECQHLLRLEDGT
TEGm245 (227) TEILETQEICSLTLEYRRECGRDSVLESLTSMNPSKEDGR----VQCRHLLRLEDGA
TEGm219 (227) IEILETQEIASLSLEYRRECGRDSVVQSVTSMDPSEVGDR----FQCQHLVRLEDGT
TEGm202 (227) IEVLETQEIASVTLEYRRECGRDSVLESLTSMSPSKEDDR----FQYQHLVRLEDGT
TEGm157 (227) IEVLETQELSSVSLEYRRECGRDSVVQSVTSVNPSEEDGR----FQCQHLVRLEDGT
TEGm203 (227) TEILETHEVCSLTLEYRRECGRDSVVQSVTSVNPSEVDDR----FEYQHLLRLEDGA
TEGm183 (227) IEVLETHEISSVSLEYRRECGRDSVLQSLTSMSPSKVGDR----FQCRHLLRLEDGT
TEGm169 (227) IEVLETHEISSVSLEYRRECGRDSVLQSLTSMSPSKVGDR----FQCRHLLRLEDGT
TEGm162 (227) IEVLETHEISSVSLEYRRECGRDSVLQSLTSMSPSKVGDR----FQCRHLLRLEDGT
TEGm546 (227) TEVLETHEIASVSLEYRRECGRDSVVQSVTSMSPSKEGDR----FQCQHLVRLEDGT
TEGm501 (227) TEVLETHELSSVSLEYRRECGRDSVVESVTSVNPSKEGGR----VEYRHLVRLEDGA
TEGm288 (227) TEVLETHEICSMSLEYRRECGRDSVLESVTSVSPSEEDGR----VEYRHLLRLEDGT
TEGm492 (227) IEVLETHELASMSLEYRRECGRDSVLQSLTSVNPSKVDDR----FQCQHLLRLEDGT
TEGm520 (227) IEILETQELASLTLEYRRECGRDSVLQSLTSVNPSKEDDR----VQCQHLLRLEDGT
TEGm250 (212) IEVLETQEVASLTLEYRRECGRDSVLESVTSMSPSEEDGR----FQCQHLVRLEDGT
TEGm413 (227) IEILETQEVASLSLEYRRECGRDSVLESVTSVDPSKVDGR----FQYRHLLRLEDGT
TEGm419 (227) TEVLETQEIASLSLEYRRECGRDSVLESLTSMDPSKVDGR----FEYQHLLRLEDGT
 CpFatB1 (227) TEVLETQELCSLTLEYRRECGRDSVLESVTSMDPSKVGDR----FQYRHLLRLEDGA
 CvFatB1 (227) IEVLETQELCSLTVEYRRECGMDSVLESVTAVDPSENGGR----SQYKHLLRLEDGT
TEGm200 (227) TEVLETHEISSLTLEYRRECGRDSVVESLTSMGPSEVDGR----FEYQHLLRLEDGA
 CvFatB2 (227) PEVLETQELCSLTLEYRRECGRESVLESLTAVDPSGEGYG----SQFQHLLRLEDGG
 UaFatB1 (229) PEIHESHEIASLTLEYRRECGRDSVLNSATKVSDSSQLGKSA--VECNHLVRLQNGG
 CnFatB2 (227) ISILENHELASMSLEYRRECGRDSVLQSLTAVSNDLTDGLVESGIECQHLLQLECGT
 CnFatB3 (227) VWMLDGYEVATMSLEYRRECRMDSVVQSLTAVSSDHADGSP---IVCQHLLRLEDGT
```

```
        (286) 286           300       310         322
TEGm198 (280) EIVKGRTEWRPKN---AGTNGAISTGKTKNS------
TEGm204 (280) EIVKGRTEWRPKN---AGTNGAISTGKTKNS------
TEGm215 (280) EIVKGRTEWRPKN---AGTNGAISTGKTKNS------
TEGm173 (280) EIVKGRTEWRPKN---AGTNGAISTGKTKNS------
TEGm181 (280) EIVKGRTEWRPKN---AGTNGAISTGKTKNS------
TEGm258 (265) EIVKGRTEWRPKN---AGTNGAISTGKTKNS------
TEGm205 (280) EIVKGRTEWRPKN---AGTNGAISTGKTKNS------
TEGm171 (265) DIMKGRTEWRPKN---AGTNGAISTGKTKNS------
TEGm201 (280) DIMKGRTEWRPKN---AGTNGAISTGKTKNS------
TEGm245 (280) EIMKGRTEWRPKN---AGTNGAISTGKTKNS------
TEGm219 (280) EFVKGRTEWRPKN---AGTNGAISTGKTKNS------
TEGm202 (280) DIVKGRTEWRPKN---AGTNGAISTGKTKNS------
TEGm157 (280) DIVKGRTEWRPKN---AGTNGAISTGKTKNS------
TEGm203 (280) DIVKGRTEWRPKN---AGTNGAISTGKTKNS------
TEGm183 (280) EIVKGRTEWRPKN---AGTNGAISTGKTKNS------
TEGm169 (280) EIVKGRTEWRPKN---AGTNGAISTGKTKNS------
TEGm162 (280) EIVKGRTEWRPKN---AGTNGAISTGKTKNS------
TEGm546 (280) EIVKGRTEWRPKN---AGTNGAISTGKTKNS------
TEGm501 (280) EIVKGRTEWRPKN---AGTNGAISTGKTKNS------
TEGm288 (280) DIMKGRTEWRPKN---AGTNGAISTGKTKNS------
TEGm492 (280) EFVKGRTEWRPKN---AGTNGAISTGKTKNS------
TEGm520 (280) DIVKGRTEWRPKN---AGTNGAISTGKTKNS------
TEGm250 (265) DIVKGRTEWRPKN---AGTNGAISTGKTKNS------
TEGm413 (280) EIMKGRTEWRPKN---AGTNGAISTGKTKNS------
TEGm419 (280) EIMKGRTEWRPKN---AGTNGAISTGKTKNS------
 CpFatB1 (280) DIMKGRTEWRPKN---AGTNGAISTGKT---------
 CvFatB1 (280) DIVKSRTEWRPKN---AGTNGAISTSTAKTSNGNSVS
TEGm200 (280) EIMKGRTEWRPKN---AGTNGAISTGKTKNS------
 CvFatB2 (280) EIVKGRTEWRPKN---AGINGVVPSEESSPGDYS---
 UaFatB1 (284) EIVKGRTVWRPKRP--LYNDGAVVDVKAKTS------
 CnFatB2 (284) ELVKGRTEWRPKHSPALGNMGPTPGGSA---------
 CnFatB3 (281) EIVRGQTEWRPKQQ--ARDLGNMGLHPTESK------
```

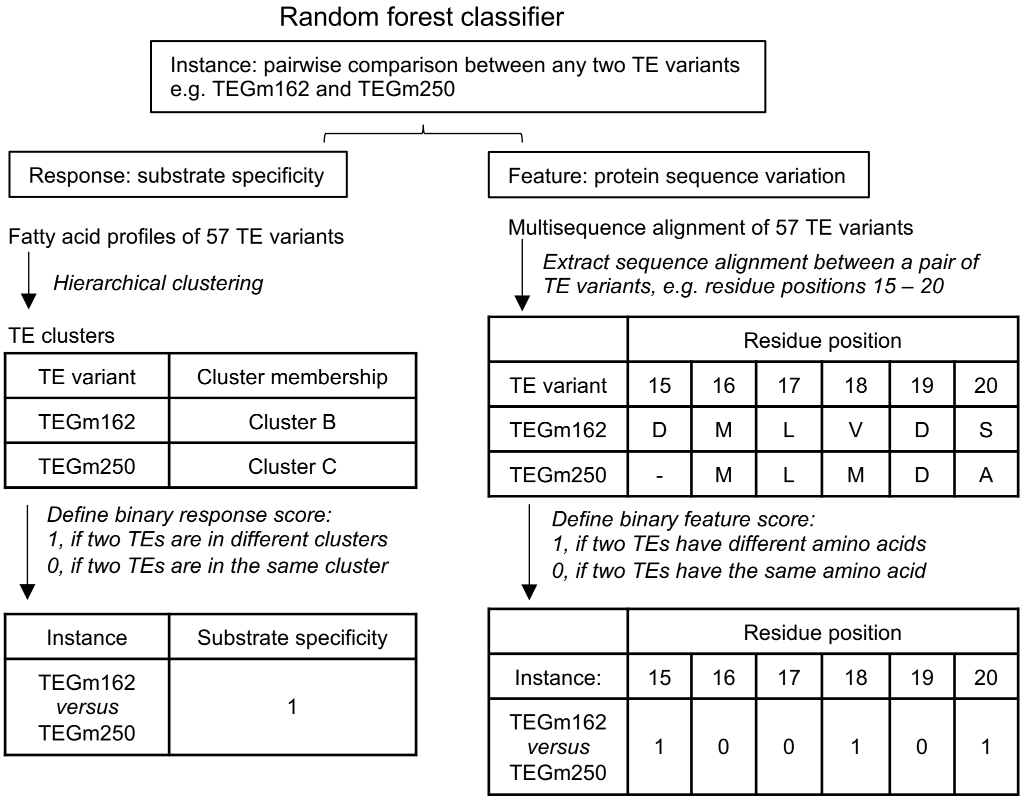

Supplementary Figure 4. Calculation of the response scores and the feature scores for the random forest classifier

1.2 Supplementary Tables

Supplementary Table 1. Nucleotide sequences of DNA primers used for generating the acyl-ACP TE variant library.

Primer Name	Sequence (5'->3')[1]
M1-1	TCATGGATCCGGAAAAACAGTGGACCMTGTTWGACYGKAAACCGARACGTCCGGACATGC
M1-2	CGAHACGACCCAGACCGAAAGVGTCCAYCAGCATGTCCGGACGTYTCGGT
M1-3	CTTTCGGTCTGGGTCGTDTCGTTMAGRACGGTCTGGTTTTCCGTCAGARC
M1-4	GATTTCGTAAGAACGGATAGAGAAGYTCTGACGGAAAACCAGACCGT
M1-5	TTCTCTATCCGTTCTTACGAAATCGGTGYTGACCGTACCGCTTCTRTCGAAACC
M1-6	GTGGTTGAKCGHGGTTTCCTGMAMGTGGTTCATCASGGTTTCGAYAGAAGCGGTACG
M1-7	CAGGAAACCDCGMTCAACCACKKCAAATCTNYCGGTCTGMTGRACGACGGTTTC
M1-8	CAGGTYACGTTTGBACATTTCCGGGGTACGACCGAAACCGTCGTYCAKCAGACC
M1-9	CCGGAAATGTVCAAACGTRACCTGATCTGGGTTNTCRCCAAAATGCAGRTCATGRTCRACC
M1-10	GTCACCCCAGGTCGGGTAACGGTYGAYCATGAYCTGCATTTTGGYGA
M2-1	CGTTACCCGACCTGGGGTGACACCNTTGAGNTCAACACCTGGNTTTCTMAATCTGGTAAAA
M2-2	CAGCCAGTCACKASSCATACCGWTTTTACCAGATTKAGAAANCCAGGTGTTG
M2-3	GTATGSSTMGTGACTGGCTGRTCHSTGACTGCMAMACCGGTGAAAYCMTCVTSCGT
M2-4	GGTTTTCTKGTTCATCATARCSYAAACAGAGGTAGCACGSABGAKGRTTTCACCGGT
M2-5	TGTTTRSGYTATGATGAACMAGAAAACCCGTARANTCTCTARANTCCCGGAAGAAGTT
M2-6	GAGTCGANGAAGTRCGGASCGANCTCCYGACGAACTTCTTCCGGGANTYTAGAGA
M2-7	TCGSTCCGYACTTCNTCGACTCTSCGSCGGYTNTCGAAGACRACGACSGTAAACTGC
M2-8	GATAGAGTCASCGSTTTTCWCGTCGANTTTCBGCAGTTTACSGTCGTYGTCTTCGA

M2-9	CGACGWGAAAASCGSTGACTCTATCARAARAGGTCTGACCCCGCGTTGGARCGAC
M2-10	AACGTGCTGGTTGANGTCGANGTCGYTCCAACGCGGGGTCAGA
M3-1	GACNTCGACNTCAACCAGCACGTTARCAACGYGAAATACNTCGGTTGGNTTCTG
M3-2	AGAAYTTCGRTCGGCAYAGATTCCAGAANCCAACCGANGTATTTCRCGTT
M3-3	GAATCTRTGCCGAYCGAARTTCTGGAAACCCAKGAGNTCKSCTCTNTG
M3-4	ACCGCATTCACGACGGTATTCCAGGSTCANAGAGSMGANCTCMTGGG
M3-5	CTGGAATACCGTCGTGAATGCGGTCGTGACTCTGTTSTGSAATCTSTTACCTCTRTG
M3-6	GANACGGYCAYCCWCTTYAGACGGGYYCAYAGAGGTAASAGATTSCASAACAGA
M3-7	CCGTCTRAAGWGGRTGRCCGTNTCSAGTRCCRGCACCTGSTGCGTCTGG
M3-8	TTCGGTACGACCTTTCAYGAWSTCAGYACCGTCTTCCAGACGCASCAGGTGCYG
M3-9	GASWTCRTGAAAGGTCGTACCGAATGGCGTCCGAAAAACGCTGGTACCAACG
M3-10	AGGTGAATTCTTGGTTTTACCGGTAGAGATAGCACCGTTGGTACCAGCGTTTTTCGG

[1] Footnote

R = A or G ; Y = C or T; M = A or C ; K = G or T; S = C or G; W = A or T; H = A or C or T ; B = C or G or T ; V = A or C or G ; D = A or G or T; N = A or C or G or T

Supplemental Table 2. Fatty acids productivity and profiles of 6 parental acyl-ACP TEs and 480 variants selected by Neutral Red staining

Colony Number	Fatty acid profiles (%) [a]												Fatty acid productivity (μM) [b]
	C4:0	C6:0	C8:0	C10:0	C10:1	C12:0	C12:1	C14:0	C14:1	C16:0	C16:1	C18:1	
m162	0.5	4.9	38.5	5.3	0.5	11.7	2.2	19.4	11.6	3.1	0.6	1.5	1695.0
m261	0.3	3.3	25.1	5.7	0.6	12.9	1.2	32.7	10.9	4.7	0.7	2.0	1642.4
m250	1.6	1.7	20.0	4.3	0.7	3.8	2.0	19.8	3.5	19.1	17.1	6.2	1618.3
m265	0.2	2.4	25.3	5.4	0.6	13.7	1.1	31.1	11.9	6.0	1.0	1.4	1610.2
m312	0.2	3.1	25.7	4.1	0.1	9.2	1.0	32.7	14.6	5.4	1.4	2.4	1578.3
m258	4.4	6.4	50.4	6.4	1.1	4.7	1.8	9.1	1.3	10.2	1.9	2.3	1557.8
m170	0.4	4.9	30.7	6.0	0.6	13.0	2.3	21.4	13.2	3.1	1.6	2.7	1552.7
m313	0.3	3.7	27.2	4.6	0.3	10.1	1.0	32.1	13.0	3.8	1.2	2.8	1532.6
m172	0.2	4.0	24.1	4.3	0.4	10.2	2.1	23.7	13.4	6.5	8.0	3.3	1504.2
m310	0.3	3.1	25.7	4.2	0.0	8.9	0.8	33.9	14.0	4.8	1.3	3.0	1489.9
m163	0.4	5.0	28.2	5.9	0.6	12.6	1.7	25.8	14.9	3.4	1.6	0.0	1486.2
m320	0.2	2.9	24.7	3.9	0.0	9.4	0.7	38.1	15.2	4.9	0.0	0.0	1483.0
m314	0.2	2.9	23.2	4.8	0.3	10.8	1.0	31.5	13.0	10.4	0.0	1.9	1444.9
m503	0.2	4.4	31.7	6.6	0.7	14.2	1.2	25.7	6.8	3.8	1.4	3.3	1444.1
m174	0.1	3.8	24.1	4.2	0.4	9.2	1.5	25.6	12.8	6.7	8.6	3.0	1433.8
m322	0.3	2.9	24.2	4.2	0.0	9.0	0.7	39.7	15.9	3.1	0.0	0.0	1428.6
m547	0.2	4.1	34.8	5.7	0.6	13.1	1.0	21.7	11.2	4.0	1.4	2.2	1427.7
m169	0.4	4.2	33.7	4.6	0.6	11.7	2.8	18.8	14.6	3.8	1.9	2.7	1426.9
m317	0.3	3.1	25.1	4.4	0.0	9.4	0.9	37.3	15.4	3.1	1.1	0.0	1422.5
m530	0.3	4.2	34.5	6.8	0.8	15.0	0.2	24.8	5.7	3.1	1.6	3.0	1411.8
m263	0.3	3.1	26.5	6.2	0.5	13.7	1.1	31.2	10.6	4.4	0.8	1.5	1392.5
m419	0.1	0.2	13.2	7.6	2.0	13.7	12.2	15.2	8.0	8.9	15.2	3.6	1391.8
m532	0.2	4.4	36.6	5.5	0.6	13.9	0.8	27.1	4.6	3.4	1.2	1.7	1377.1
m525	0.3	4.1	32.6	6.5	0.9	15.5	0.8	22.3	9.2	3.6	1.3	3.1	1367.6
m311	0.3	3.3	25.2	4.8	0.0	10.1	1.0	33.9	14.6	4.8	0.9	1.0	1364.4
m283	0.3	2.7	27.5	6.4	0.4	12.4	0.8	30.1	11.3	6.4	0.5	1.2	1361.4
m505	0.3	4.2	30.8	6.0	0.7	13.3	0.9	25.7	7.9	4.1	1.5	4.7	1359.9
m499	0.3	4.2	31.8	6.5	0.7	15.4	0.7	27.2	6.1	3.0	1.3	2.9	1349.8
m514	0.3	4.0	33.3	6.8	0.8	13.8	1.1	24.8	5.6	3.2	1.4	4.9	1346.1
m543	0.3	3.9	33.4	5.6	0.6	13.2	0.9	24.3	11.3	3.0	1.2	2.5	1342.0
m164	0.5	5.6	29.4	6.5	0.6	11.9	1.9	22.8	13.0	3.2	1.6	3.0	1339.7
m179	0.5	5.3	33.4	5.5	0.6	12.0	2.0	18.4	13.8	3.6	2.1	2.7	1337.5
m552	0.2	3.7	32.4	6.0	0.7	15.2	0.8	21.7	10.0	5.6	1.2	2.5	1325.6
m175	0.1	3.6	23.7	4.0	0.4	10.2	1.8	28.2	15.4	5.0	4.3	3.3	1317.2
m526	0.3	3.9	31.7	6.3	0.8	14.2	0.7	25.5	9.4	3.2	1.2	2.8	1313.2
m566	0.1	3.7	33.8	5.8	0.6	13.6	1.0	24.3	9.6	3.8	1.0	2.6	1313.2
m593	0.0	3.5	37.1	5.8	0.6	13.9	0.0	21.1	11.3	3.2	1.2	2.4	1312.8
m161	0.6	6.3	35.7	5.9	0.6	11.9	2.4	18.5	10.9	2.8	1.8	2.7	1312.0
m264	0.3	3.1	24.7	4.8	0.4	13.4	0.8	33.5	11.6	4.7	1.2	1.6	1309.0
m512	0.1	4.2	36.3	6.4	0.7	13.6	0.7	26.3	6.0	3.0	1.1	1.5	1306.8
m504	0.3	4.6	34.1	6.4	0.7	13.2	0.9	26.3	7.3	2.4	1.4	2.4	1305.6
m316	0.1	3.0	24.1	4.5	0.0	10.1	0.7	36.5	15.8	5.2	0.0	0.0	1300.1
m515	0.3	5.1	34.6	7.2	0.8	15.0	1.1	24.3	5.2	2.5	1.4	2.3	1298.9
m267	0.1	2.8	27.7	5.4	0.5	12.7	1.2	31.8	11.0	5.0	0.8	0.9	1293.9
m271	0.3	3.4	27.9	6.1	0.5	14.9	0.9	27.3	10.2	6.7	0.6	1.2	1292.5
m214	0.2	0.2	1.6	0.1	0.0	2.0	0.4	36.1	1.8	18.6	35.5	3.5	1290.4
m594	0.1	3.0	29.3	5.4	0.5	12.8	0.8	26.3	14.7	3.3	1.6	2.1	1287.1
m474	0.2	3.5	28.5	5.2	0.5	12.6	0.9	29.8	8.9	4.8	1.2	3.8	1283.5
m321	0.1	3.0	25.4	4.2	0.0	9.4	0.7	36.4	16.6	4.2	0.0	0.0	1280.7
m330	0.3	3.2	24.8	4.3	0.0	9.0	0.5	36.7	15.7	5.7	0.0	0.0	1280.2
m506	0.3	4.4	32.3	6.5	0.7	14.5	0.8	29.3	5.8	3.7	1.8	0.0	1276.2
m472	0.1	3.2	26.9	5.3	0.6	13.1	1.0	29.3	9.2	5.7	1.2	4.4	1274.2
m166	0.4	4.7	30.2	5.4	0.6	11.9	2.4	21.8	13.6	3.6	2.0	3.4	1271.9
m487	0.1	3.5	28.2	5.6	0.6	13.7	0.8	30.2	7.7	4.0	1.4	4.3	1266.1
m529	0.3	4.5	32.5	6.3	0.7	9.7	1.2	31.2	6.6	3.3	1.4	2.3	1264.2
m469	0.2	3.3	27.8	5.4	0.6	13.8	1.0	27.7	9.0	6.0	1.3	3.8	1264.0
m595	0.2	3.5	32.4	6.6	0.8	14.7	1.2	20.5	13.1	3.3	1.3	2.3	1261.2
m344	0.2	2.9	24.3	3.9	0.0	9.8	0.6	37.1	12.4	8.7	0.0	0.0	1259.9
m563	0.1	3.8	34.6	5.9	0.7	13.8	1.0	21.5	11.8	3.8	1.1	2.0	1258.5
m293	0.3	2.7	26.3	6.1	0.5	14.8	1.0	30.4	9.8	5.5	1.0	1.8	1258.0
m288	0.2	0.3	30.9	5.5	2.2	9.8	8.5	12.9	7.1	12.1	8.3	2.2	1256.4
m567	0.1	3.6	34.7	6.0	0.7	13.3	0.9	23.3	10.0	3.0	1.6	2.6	1248.1
m440	0.2	3.0	25.5	4.8	0.6	12.3	1.0	32.0	10.3	5.0	1.0	4.2	1246.5
m154	0.6	5.7	33.2	6.1	0.7	12.0	2.7	18.4	13.4	2.7	1.7	2.8	1244.0
m466	0.1	3.7	31.8	5.8	0.6	13.7	1.0	26.0	10.1	2.8	1.2	3.4	1242.9
m309	0.2	2.3	20.4	3.8	0.0	7.7	0.6	44.2	16.9	3.1	0.7	0.0	1239.3
m183	0.4	0.5	12.7	4.2	0.8	9.2	6.6	23.2	11.7	10.0	18.4	2.4	1235.8
m491	0.2	3.6	30.2	5.5	0.6	13.2	1.0	28.7	7.8	3.4	1.9	3.9	1228.6

m540	0.2	3.9	34.2	5.9	0.8	13.2	1.1	22.9	9.7	5.1	1.0	2.0	1226.5
m173	0.3	0.3	2.8	0.4	0.0	3.5	0.5	35.9	3.1	12.0	36.2	4.9	1225.8
m542	0.2	3.3	30.8	5.8	0.6	12.9	0.8	26.5	12.5	3.3	1.0	2.3	1219.1
m471	0.2	3.6	28.6	5.3	0.6	13.5	1.0	26.8	8.8	5.8	1.5	4.1	1214.0
m467	0.2	3.2	28.7	5.4	0.5	12.9	0.8	29.2	9.9	3.8	1.3	4.1	1212.3
m301	0.0	0.0	0.1	0.4	0.1	5.2	1.4	40.8	8.2	17.2	26.5	0.0	1208.8
m541	0.2	3.8	32.9	6.2	0.8	13.2	0.5	23.2	11.7	3.5	1.2	2.8	1208.4
m569	0.2	3.9	36.9	6.3	0.8	14.1	1.2	22.7	9.2	3.2	1.5	0.0	1204.4
m204	0.1	0.2	1.3	0.1	0.0	1.6	0.3	38.0	1.5	19.8	33.6	3.4	1202.6
m634	0.2	4.2	38.7	6.3	0.8	13.8	0.6	16.7	13.5	2.8	1.0	1.5	1197.2
m486	0.2	3.7	27.2	6.0	0.7	14.3	0.6	29.2	8.3	4.2	1.5	4.2	1192.6
m343	0.3	2.6	23.1	3.8	0.0	9.0	0.7	39.6	14.5	6.4	0.0	0.0	1190.0
m548	0.2	3.6	33.4	6.5	0.8	14.9	1.2	25.3	6.7	3.7	1.3	2.5	1184.9
m341	0.3	3.0	24.8	4.2	0.0	10.0	0.6	36.6	13.8	6.7	0.0	0.0	1183.4
m436	0.2	3.1	26.3	5.5	0.6	13.5	1.0	29.8	10.7	4.7	1.0	3.5	1183.3
m315	0.3	3.4	26.1	5.5	0.4	12.1	1.5	26.6	14.0	5.1	1.6	3.5	1183.0
m269	0.4	3.4	27.6	5.9	0.4	13.0	1.1	29.2	11.8	5.4	0.6	1.2	1180.6
m490	0.3	4.4	33.3	6.1	0.6	13.5	0.9	25.8	7.7	3.5	1.2	2.7	1178.9
m575	0.0	3.7	34.8	6.1	0.7	13.4	1.3	23.0	11.4	3.7	1.5	0.5	1178.1
m589	0.0	3.7	37.7	6.1	0.7	13.9	0.0	21.8	9.6	3.4	1.1	2.0	1173.9
m608	0.1	3.8	38.7	5.8	0.6	14.0	0.5	20.0	10.5	3.6	0.6	1.7	1173.3
m446	0.3	3.6	27.1	5.6	0.0	13.5	1.3	28.5	10.1	4.0	1.2	4.8	1170.9
m610	0.0	4.0	35.0	6.0	0.7	13.7	0.0	20.4	12.9	3.6	0.9	2.7	1168.4
m210	0.4	0.3	1.7	0.1	0.0	2.0	0.4	33.4	1.3	19.4	36.5	4.5	1167.2
m346	0.1	3.2	26.4	4.5	0.0	9.5	0.6	36.5	13.7	5.5	0.0	0.0	1166.7
m335	0.4	3.5	27.0	4.1	0.0	9.8	0.6	33.8	14.0	6.9	0.0	0.0	1165.9
m443	0.3	3.5	26.9	5.1	0.0	12.5	1.0	32.3	10.5	3.7	0.9	3.3	1164.8
m621	0.1	4.7	41.1	6.0	0.7	11.9	0.1	17.8	13.3	3.3	1.0	0.0	1160.8
m434	0.2	3.3	26.5	5.4	0.5	13.0	1.2	30.2	11.8	3.6	1.0	3.2	1159.8
m182	0.3	0.3	2.1	0.3	0.0	2.7	0.4	33.7	2.2	17.7	34.5	6.0	1156.7
m629	0.0	3.5	36.3	6.0	0.7	13.5	0.1	18.5	14.6	3.5	1.0	2.3	1154.1
m177	0.2	4.4	26.0	4.6	0.4	11.3	1.7	24.6	14.2	5.8	4.1	2.8	1152.9
m580	0.2	4.0	37.8	6.4	0.8	14.5	1.0	21.4	9.0	2.9	0.8	1.1	1151.4
m600	0.1	4.0	35.6	5.9	0.6	14.5	0.0	21.7	11.9	3.8	1.3	0.6	1149.4
m528	0.2	3.4	31.0	5.7	0.6	13.0	0.0	33.9	5.8	3.0	1.1	2.1	1146.8
m413	0.1	0.3	14.8	6.6	1.4	13.6	7.6	14.2	5.9	14.7	16.9	3.9	1141.7
m155	0.6	5.5	31.9	5.2	0.4	12.3	2.4	20.5	13.6	3.2	1.7	2.8	1135.0
m586	0.1	4.3	36.4	6.8	0.8	15.0	1.4	17.4	12.0	3.5	1.0	1.3	1128.9
m518	0.3	3.9	33.9	6.2	0.8	14.2	0.8	28.0	4.4	3.8	1.1	2.7	1127.6
m588	0.2	3.6	33.5	5.8	0.7	13.0	0.9	20.7	13.2	5.0	1.1	2.2	1126.3
m460	0.2	3.2	28.2	5.4	0.6	13.5	1.0	27.8	9.8	5.2	1.2	3.7	1126.2
m318	0.2	2.4	22.5	4.4	0.0	9.7	0.7	40.7	15.2	4.1	0.0	0.0	1120.9
m437	0.3	3.1	25.4	4.9	0.5	12.2	1.0	32.7	10.9	3.4	1.2	4.5	1119.6
m171	1.3	1.4	17.6	0.8	0.1	6.4	3.1	26.2	5.9	12.1	23.0	2.0	1118.5
m527	0.3	3.9	34.6	6.1	0.8	14.2	1.0	25.3	6.6	3.9	1.1	2.4	1114.6
m193	0.3	5.3	31.3	4.4	0.4	13.9	2.1	21.2	12.1	4.5	1.8	2.7	1114.2
m617	0.1	3.6	35.0	5.9	0.7	13.3	0.6	20.9	14.5	4.5	0.7	0.1	1110.0
m607	0.0	4.2	37.0	6.1	0.7	12.7	0.2	21.3	11.8	3.2	1.0	1.8	1109.7
m245	0.1	0.0	1.0	0.0	0.0	0.7	0.0	32.1	0.0	27.7	38.5	0.0	1105.6
m327	0.1	3.1	25.5	4.5	0.0	9.8	0.7	30.9	16.5	8.9	0.0	0.0	1105.3
m319	0.1	2.5	22.9	4.2	0.1	9.8	0.7	37.5	13.5	5.6	0.0	3.2	1102.9
m484	0.3	3.9	28.4	6.2	0.7	13.9	0.8	29.1	7.9	4.7	1.2	3.1	1100.0
m517	0.2	3.5	31.7	5.8	0.7	13.0	0.8	29.8	5.7	4.5	1.3	2.9	1085.3
m156	0.6	5.4	32.5	5.1	0.5	12.2	2.0	19.9	13.9	3.4	1.8	2.8	1084.1
m430	0.2	3.1	24.9	4.7	0.2	11.7	0.8	37.5	11.2	5.5	0.0	0.0	1079.9
m420	0.3	3.6	25.1	5.7	0.6	12.5	1.2	30.4	11.6	7.8	1.2	0.0	1078.7
m149	0.4	3.8	23.6	4.4	0.4	10.8	1.9	27.5	18.0	4.7	1.6	2.9	1078.4
m478	0.1	3.4	30.0	5.1	0.5	12.3	0.8	30.1	9.4	4.5	1.0	2.9	1071.5
m207	0.1	0.2	1.3	0.2	0.0	1.9	0.3	35.2	1.1	21.9	33.9	3.7	1070.3
m502	0.4	4.7	31.4	5.8	0.7	13.8	1.2	21.5	7.2	8.3	2.0	3.0	1068.7
m180	0.1	3.1	23.8	5.3	0.4	13.0	1.6	27.6	14.8	5.0	2.2	3.1	1067.8
m218	0.4	3.2	24.3	5.7	0.4	12.5	0.7	32.3	9.5	8.5	2.5	0.0	1067.3
m326	0.1	2.0	19.4	3.2	0.0	7.6	0.5	43.5	14.8	8.9	0.0	0.0	1063.5
m150	0.5	4.4	27.3	4.8	0.4	11.3	1.7	24.4	15.2	4.4	1.8	3.8	1061.7
m426	0.2	3.3	27.7	5.5	0.3	13.2	1.1	27.3	12.4	4.2	1.5	3.3	1060.1
m442	0.3	3.3	26.3	5.2	0.0	12.9	1.0	29.4	10.2	6.1	1.3	4.0	1059.3
m152	0.5	5.2	30.4	4.9	0.5	11.9	2.4	23.9	15.0	3.6	1.8	0.0	1058.7
m329	0.3	3.1	26.1	4.6	0.0	10.1	0.5	31.6	16.7	7.1	0.0	0.0	1056.1
m433	0.1	3.4	26.7	5.1	0.6	12.8	1.1	30.3	10.7	4.9	0.9	3.3	1053.3
m168	0.4	4.5	28.6	4.5	0.4	11.8	2.0	23.1	13.3	5.5	1.9	3.9	1051.5
m633	0.0	3.9	41.7	5.9	0.8	13.0	0.4	16.6	11.7	3.8	0.9	1.4	1050.6
m439	0.2	3.3	27.6	5.1	0.5	12.3	1.1	28.0	11.4	5.0	1.3	4.1	1050.3
m331	0.1	2.9	24.9	4.3	0.0	10.1	0.5	32.7	14.8	9.6	0.0	0.0	1044.2
m282	0.3	3.0	28.0	6.1	0.5	13.6	0.8	29.3	11.4	6.3	0.8	0.0	1043.9

m421	0.3	3.4	26.3	5.4	0.4	12.1	1.0	30.6	11.4	4.6	1.3	3.1	1043.2
m422	0.3	3.1	25.2	5.1	0.3	12.6	1.0	33.2	10.4	4.3	1.2	3.3	1037.9
m336	0.2	2.4	21.5	3.8	0.0	9.1	0.4	41.5	13.3	7.8	0.0	0.0	1037.1
m334	0.1	2.0	19.0	3.6	0.0	8.6	0.4	42.7	14.1	9.5	0.0	0.0	1035.1
m498	0.2	3.3	27.4	6.2	0.8	13.9	0.9	31.4	6.8	4.5	1.3	3.2	1029.6
m151	0.6	5.0	27.9	4.9	0.4	11.6	2.3	22.8	14.7	4.8	1.9	3.2	1025.5
m475	0.2	3.2	27.6	5.1	0.6	13.7	1.0	30.5	9.1	4.1	1.3	3.7	1025.0
m425	0.4	3.8	27.6	5.3	0.3	13.3	1.1	30.8	10.1	6.4	1.0	0.0	1022.5
m296	0.3	2.9	27.4	6.7	0.4	10.1	1.4	31.9	12.3	5.7	0.8	0.0	1022.2
m520	0.0	0.2	2.4	3.1	1.5	8.8	10.3	24.0	15.0	11.4	20.6	2.8	1020.6
m611	0.1	3.3	30.7	5.6	0.6	12.7	1.4	24.1	13.5	4.2	1.0	2.7	1016.0
m438	0.3	3.1	24.9	5.2	0.0	13.2	1.3	32.2	10.6	4.4	1.4	3.6	1016.0
m445	0.3	3.8	27.3	5.2	0.0	12.5	1.1	30.3	10.2	4.7	1.2	3.3	1014.7
m202	0.4	0.4	2.8	0.3	0.0	4.0	0.8	30.1	2.9	17.9	36.4	4.0	1001.2
m339	0.1	2.6	23.3	4.0	0.0	9.3	0.6	39.0	13.0	8.1	0.0	0.0	994.8
m178	0.6	5.3	29.8	5.3	0.4	12.3	1.7	20.1	15.0	4.9	2.4	2.1	989.8
m565	0.3	4.2	39.0	1.9	5.3	12.3	0.9	19.5	10.3	3.7	1.1	1.6	989.6
m435	0.3	2.9	24.5	4.8	0.4	12.1	0.9	31.6	11.5	5.8	1.5	3.9	986.0
m427	0.3	3.0	25.3	5.0	0.2	12.4	1.0	32.7	10.3	4.7	1.3	3.8	982.4
m423	0.4	3.6	27.8	5.6	0.5	12.5	1.2	28.5	10.8	5.6	0.0	3.6	981.2
m215	1.6	1.0	9.0	0.7	0.1	5.6	1.7	30.6	6.5	15.2	24.3	3.7	977.5
m571	0.0	4.0	34.3	6.5	0.7	14.7	0.0	20.1	12.4	4.3	1.1	2.0	973.6
m328	0.1	1.8	16.8	3.2	0.0	6.9	0.3	47.7	15.7	7.5	0.0	0.0	969.2
m441	0.2	3.4	28.2	4.9	0.0	12.0	0.9	28.1	11.5	5.0	1.3	4.3	963.7
m428	0.3	3.2	24.5	5.2	0.2	11.9	0.9	32.0	10.3	6.1	1.4	4.2	961.2
m200	0.3	0.0	1.8	0.4	0.1	4.7	3.7	19.2	8.3	16.6	41.5	3.5	944.1
m211	0.0	0.3	1.4	0.1	0.0	1.5	0.2	35.6	1.2	18.9	40.9	0.0	943.4
m592	0.1	3.0	31.5	6.1	0.7	14.7	0.8	22.7	12.5	4.2	1.1	2.5	940.0
m345	0.1	2.8	25.1	4.1	0.0	9.7	0.0	34.2	14.1	9.9	0.0	0.0	936.5
m158	0.6	5.0	30.8	4.6	0.4	11.6	2.1	19.9	11.9	6.5	3.8	2.7	934.2
CpFatB1	0.01	1.51	93.46	2.18	0.44	0.87	0.21	0.00	0.10	1.21	0.00	0.00	932.09
m212	0.1	0.3	1.2	0.1	0.0	1.3	0.1	39.1	1.4	18.0	38.5	0.0	929.4
m342	0.2	2.4	22.8	3.8	0.0	9.1	0.5	39.7	12.7	8.8	0.0	0.0	899.5
m205	0.0	0.1	0.3	0.0	0.0	0.7	0.0	35.9	1.1	23.9	34.1	3.9	890.8
m501	0.0	0.1	0.4	0.4	0.0	9.1	1.0	38.7	4.9	17.1	23.7	4.6	867.6
m449	0.4	3.4	25.4	5.3	0.0	12.7	1.0	29.8	10.0	7.6	1.3	3.1	854.0
m418	0.2	0.0	1.1	0.0	0.0	1.7	1.2	26.3	2.7	27.7	30.6	8.4	850.6
m333	0.0	2.0	19.5	3.3	0.0	8.6	0.6	34.8	16.1	15.2	0.0	0.0	848.8
m630	0.0	4.0	38.2	6.1	0.6	12.5	0.6	16.1	13.1	5.8	0.9	2.1	847.7
m165	0.4	4.1	26.8	4.3	0.4	9.3	1.6	27.0	15.0	6.3	1.8	3.0	847.0
m188	0.5	4.8	26.2	4.8	0.4	11.4	1.9	24.7	14.8	5.8	2.2	2.4	833.6
m181	0.4	0.5	3.7	0.7	0.0	4.8	0.5	35.5	4.6	15.1	29.2	5.0	828.7
m167	0.4	4.2	27.1	5.1	0.4	11.8	1.9	26.3	15.0	6.3	1.6	0.0	814.6
m195	0.5	5.1	32.3	5.2	0.5	10.2	1.6	19.2	13.1	7.4	2.7	2.3	808.8
m476	0.3	3.8	29.0	5.2	0.5	12.7	1.3	22.4	9.2	10.1	0.9	4.4	803.0
m302	0.2	0.0	0.3	0.5	0.0	4.4	1.0	35.7	14.6	21.4	21.9	0.0	799.1
m301	0.2	0.0	2.8	5.7	1.4	17.9	7.7	24.6	12.1	15.5	12.3	0.0	798.7
m624	0.1	3.0	35.1	5.8	0.6	12.4	0.2	22.1	15.1	4.5	1.1	0.0	796.8
m570	0.2	0.5	30.2	5.8	0.6	13.2	1.1	27.4	12.5	5.5	1.1	2.0	796.6
m480	0.3	2.9	26.9	5.0	0.5	10.9	0.5	32.5	10.2	5.5	1.3	3.5	789.7
m551	0.4	7.5	15.9	8.5	0.9	19.7	1.6	26.5	6.9	7.3	1.9	2.8	777.9
m481	0.1	2.9	26.7	5.0	0.5	10.7	0.4	35.0	9.5	5.0	1.0	3.2	776.9
m412	0.0	0.0	2.2	0.0	0.0	3.8	1.7	28.2	3.4	26.8	29.1	4.7	760.6
m197	0.1	0.3	1.4	0.1	0.0	1.4	0.1	37.9	1.4	22.7	33.1	1.4	759.1
m187	0.7	4.7	30.4	5.2	0.6	12.1	2.0	20.7	14.2	7.3	2.0	0.1	753.6
m596	0.0	3.3	31.8	5.1	0.5	10.8	0.0	24.5	15.5	5.1	1.4	2.1	750.9
m251	0.1	0.1	1.0	0.2	0.0	0.9	0.0	17.0	0.5	36.3	26.3	17.6	750.1
m199	0.1	0.2	1.1	0.1	0.0	1.5	0.2	35.6	1.0	20.9	35.5	3.8	737.5
m254	0.2	3.4	31.7	5.4	0.4	12.0	0.8	20.3	11.4	12.1	0.9	1.4	731.8
m256	0.3	3.9	28.2	6.6	0.5	13.7	0.9	23.4	12.9	8.3	1.5	0.0	724.4
m495	0.4	4.0	30.9	5.3	0.6	11.6	0.9	25.7	8.3	7.7	1.8	2.7	706.4
m198	0.1	0.3	1.3	0.1	0.0	1.4	0.1	36.4	0.7	23.0	36.6	0.0	704.5
m191	0.2	3.9	27.1	4.6	0.4	11.0	1.6	27.2	16.3	6.2	1.6	0.0	704.0
m561	0.1	3.1	28.8	5.5	0.6	12.7	1.0	28.1	10.7	6.3	1.3	1.9	701.7
m303	0.0	0.1	4.9	2.8	0.3	14.2	5.4	34.7	11.1	15.6	11.0	0.0	682.3
m479	0.1	3.4	27.0	4.9	0.5	12.1	0.8	28.1	10.4	8.1	0.8	3.9	681.9
m424	0.3	2.6	23.6	4.7	0.2	11.9	0.8	33.1	12.9	9.8	0.0	0.0	678.1
m415	0.0	0.3	2.1	0.0	0.0	3.5	1.7	27.2	3.6	26.5	28.2	7.0	667.8
m189	0.6	3.9	26.1	4.8	0.4	12.6	1.7	26.1	13.4	6.7	2.4	1.4	652.3
m184	1.2	2.0	14.5	2.1	0.2	10.7	2.9	24.2	10.2	14.5	16.5	0.9	643.3
m176	0.6	0.9	8.4	1.2	0.0	7.8	1.2	39.5	10.0	14.0	16.3	0.0	630.3
m192	0.5	3.4	27.8	5.8	0.6	13.4	2.1	25.6	13.5	5.5	1.9	0.0	628.3
m507	0.1	4.2	31.2	5.3	0.5	12.0	0.6	27.1	8.5	7.0	0.8	2.5	624.3
m248	0.4	0.0	0.5	0.3	0.0	15.7	0.7	42.0	10.9	21.6	8.0	0.0	621.6

m444	0.5	3.7	27.6	4.9	0.0	12.1	0.9	24.9	10.9	9.7	0.9	4.0	615.7
m186	0.2	4.5	27.1	3.9	0.4	10.1	1.6	24.4	14.6	10.0	2.6	0.5	614.4
m262	0.3	1.5	11.8	2.5	0.1	5.7	0.2	46.8	15.5	15.6	0.0	0.0	609.2
m546	0.0	0.3	1.3	5.2	0.6	21.1	7.1	22.1	11.7	14.9	12.2	3.4	607.4
m492	0.2	0.2	2.5	1.5	0.0	8.6	0.0	21.9	2.8	25.7	24.0	12.5	603.7
m157	0.4	1.0	12.0	0.7	0.0	4.0	0.5	30.9	4.6	19.1	19.2	7.6	598.0
m618	0.1	4.1	36.4	5.4	0.6	13.3	0.0	18.3	14.4	6.3	0.9	0.0	595.1
m201	0.1	0.2	1.3	0.1	0.0	1.2	0.1	19.3	1.3	28.4	41.1	7.0	594.0
m160	0.4	3.3	24.4	4.2	0.3	10.3	1.3	29.0	15.9	9.1	2.0	0.0	582.3
m400	0.0	0.3	8.0	11.0	0.3	13.1	5.2	27.4	9.5	19.9	5.3	0.0	580.4
m473	0.2	3.1	24.4	4.4	0.4	12.1	0.0	22.3	9.6	17.1	1.2	5.3	572.5
m493	0.0	0.2	0.3	0.2	0.0	6.0	0.0	37.1	4.1	21.2	25.1	5.8	558.5
m294	0.2	0.1	2.4	6.7	0.5	9.3	2.7	29.2	5.0	23.2	17.1	3.7	530.2
m219	0.1	0.5	2.4	0.3	0.0	4.4	0.2	54.7	3.8	16.1	17.5	0.0	528.2
m203	0.1	0.1	0.6	0.1	0.0	2.4	0.0	45.1	2.2	24.1	19.6	5.7	525.5
m601	0.2	3.0	29.2	5.7	0.4	14.6	0.1	24.1	9.9	9.4	1.2	2.1	514.2
m194	0.8	3.0	22.6	3.1	0.3	10.2	2.1	21.3	9.5	15.4	7.7	4.1	507.5
m305	0.0	0.0	0.0	1.1	0.0	5.6	0.0	40.9	10.9	23.4	18.1	0.0	487.0
CvFatB2	0.00	0.00	0.67	0.56	0.15	0.31	0.24	27.85	0.00	39.95	11.22	19.06	484.14
m142	0.7	3.2	24.8	4.5	0.4	11.7	0.7	19.7	10.9	17.0	1.0	5.3	481.9
m337	0.2	1.7	13.4	0.1	0.0	1.6	0.0	16.9	1.6	25.8	27.5	11.2	481.7
m147	0.8	2.2	16.8	3.1	0.1	11.1	0.4	22.7	7.3	31.9	1.5	2.2	479.5
m623	0.1	2.6	31.2	5.5	0.6	12.0	0.0	23.2	15.3	8.6	0.9	0.0	468.6
m144	0.3	3.4	26.0	4.4	0.4	10.6	0.6	21.2	10.1	17.6	1.0	4.3	457.1
m148	0.2	3.0	23.9	3.9	0.3	10.7	0.5	24.0	9.8	20.4	1.2	2.0	456.2
m411	0.4	0.4	2.3	0.0	0.0	4.2	0.8	30.1	3.0	30.7	28.1	0.0	455.7
m141	0.7	3.2	24.6	4.4	0.4	11.7	0.7	20.1	9.9	17.6	1.3	5.5	433.9
m609	0.0	3.9	29.8	4.7	0.5	11.5	0.0	24.8	8.3	13.4	0.8	2.4	419.2
m206	0.7	1.3	8.7	3.7	0.8	6.3	2.9	24.0	7.4	27.6	16.7	0.0	414.1
m308	0.0	3.1	21.4	3.1	0.0	8.0	0.0	28.9	14.7	20.7	0.0	0.0	400.3
m213	0.1	0.3	0.5	0.0	0.0	0.8	0.0	24.9	0.3	43.2	29.9	0.0	397.8
m216	0.5	0.2	1.2	0.1	0.0	1.5	0.0	35.3	1.3	29.8	30.1	0.0	387.4
m290	0.2	0.0	5.5	3.4	0.4	9.7	5.6	25.4	7.6	31.0	9.9	1.3	383.5
m287	3.8	1.3	11.0	1.3	0.1	6.9	3.8	29.1	5.9	29.1	7.8	0.0	379.4
m103	0.9	3.6	30.0	4.6	0.4	11.7	0.8	22.0	10.8	9.9	1.1	4.1	379.1
m196	0.0	3.2	29.7	4.6	0.5	11.6	0.8	22.3	12.4	10.2	1.3	3.4	379.0
m130	0.7	3.3	25.3	4.1	0.3	10.5	0.7	24.0	11.6	14.6	0.9	3.8	378.6
m416	0.4	0.5	1.9	0.6	0.0	3.9	1.5	28.3	4.3	32.0	26.7	0.0	375.7
m139	0.6	3.4	26.7	4.8	0.4	11.0	0.8	23.7	10.4	12.6	2.2	3.3	375.4
m208	0.2	0.2	0.9	0.2	0.0	1.5	0.0	29.9	0.5	34.1	32.5	0.0	371.5
m128	0.8	2.9	23.9	3.8	0.2	11.1	0.5	21.8	8.7	22.0	1.3	2.9	370.6
m153	12.9	6.5	20.7	5.6	1.0	6.6	2.4	20.5	8.5	9.9	0.0	5.3	364.5
UaFatB1	0.07	0.31	21.79	19.72	0.93	3.56	1.95	32.23	0.27	8.37	6.27	4.54	351.80
m123	0.8	3.9	31.8	4.6	0.4	10.6	0.6	23.4	10.0	10.1	0.5	3.2	349.0
m190	0.7	3.4	19.5	3.5	0.1	9.4	0.5	35.8	10.7	16.5	0.0	0.0	348.2
m249	0.4	1.7	13.5	2.6	0.1	10.3	0.2	25.8	7.3	35.6	2.4	0.0	346.1
m217	0.3	0.2	0.7	0.0	0.0	1.1	0.0	28.6	0.4	45.2	23.4	0.0	345.7
m111	0.0	3.3	29.1	4.5	0.5	11.8	0.7	23.0	11.7	9.8	1.8	3.8	341.5
m574	0.0	4.1	35.6	6.0	0.4	13.3	0.0	21.6	6.0	12.0	0.6	0.5	339.7
m285	0.0	0.0	3.3	5.1	0.1	5.0	0.0	24.8	0.3	46.5	14.9	0.0	334.5
m304	0.7	0.2	4.3	0.0	0.0	6.0	1.9	45.3	6.0	24.2	11.3	0.0	325.5
m302	0.4	0.5	2.3	5.4	0.0	16.8	4.3	24.2	19.5	16.0	10.6	0.0	324.2
m110	0.0	3.3	26.1	4.2	0.3	10.9	0.5	27.4	10.2	10.5	2.9	3.8	324.1
m124	0.9	3.3	29.3	4.7	0.4	11.4	0.0	23.4	11.2	11.3	0.6	3.6	322.1
m119	0.8	3.4	29.8	4.4	0.5	10.8	0.0	20.8	11.7	12.0	1.2	4.7	319.8
m401	0.3	0.0	1.8	3.4	0.2	11.5	4.3	24.1	9.0	32.0	13.5	0.0	316.5
m129	0.9	3.1	23.6	4.0	0.2	10.2	0.7	22.9	9.7	19.5	1.3	3.9	316.2
m108	0.0	3.4	28.4	4.4	0.6	11.5	0.8	23.9	11.4	10.5	1.3	3.7	315.0
m118	0.8	3.6	28.5	4.4	0.3	11.5	0.0	24.2	11.1	13.8	1.7	0.0	311.6
m105	0.0	2.8	23.3	3.8	0.1	11.4	0.7	27.8	11.5	13.1	1.1	4.4	310.1
m409	0.7	0.8	9.9	4.7	0.2	17.8	2.0	28.4	9.7	23.6	2.4	0.0	307.6
m122	0.0	3.8	31.7	4.7	0.5	11.0	0.0	21.0	9.8	13.5	1.0	3.1	306.9
m306	0.3	0.0	1.6	0.1	0.0	1.5	0.0	34.6	0.0	43.5	18.4	0.0	301.0
m146	0.9	0.5	1.5	0.1	0.0	4.3	0.0	20.9	0.5	52.1	16.2	2.9	298.0
m587	0.1	2.9	21.5	4.5	0.3	10.5	0.0	31.9	11.8	14.0	0.9	1.7	297.9
m544	0.3	4.4	29.3	5.3	0.3	12.1	0.0	24.7	5.8	17.9	0.0	0.0	291.8
m133	0.9	3.5	25.3	4.2	0.4	9.4	0.7	23.8	9.9	15.4	2.7	3.9	286.3
m230	0.2	0.2	0.8	0.0	0.0	7.2	0.0	50.0	3.9	21.8	15.7	0.0	285.1
m408	0.4	1.0	22.7	5.6	0.1	16.3	4.1	15.5	5.1	25.3	4.0	0.0	284.8
m447	0.5	0.0	3.1	2.3	0.0	11.3	4.1	27.1	13.9	26.4	11.3	0.0	284.1
m448	0.0	0.2	1.1	0.9	0.0	11.8	1.1	39.6	5.4	28.7	11.1	0.0	282.3
m104	0.0	3.5	30.3	4.5	0.4	11.0	0.9	25.6	12.3	11.6	0.0	0.0	282.0
m136	0.9	3.3	26.1	4.2	0.3	12.0	0.6	25.8	9.8	16.1	0.9	0.0	281.6
m270	1.2	2.2	14.4	3.1	0.0	8.8	0.0	36.8	9.4	24.1	0.0	0.0	279.1

m519	0.4	3.2	24.7	4.7	0.3	10.5	0.3	33.8	6.1	15.9	0.0	0.0	278.3
m275	1.2	3.5	24.2	4.9	0.1	9.2	0.2	23.1	7.5	26.1	0.0	0.0	275.2
m102	0.0	3.6	30.5	4.5	0.4	10.5	0.8	23.6	9.4	11.9	1.7	3.2	271.2
m113	0.0	0.6	4.5	0.7	0.0	6.1	0.0	28.8	2.7	28.4	21.3	7.0	270.6
CnFatB2	0.00	0.00	0.14	0.00	0.00	0.00	0.00	13.77	0.00	52.57	7.45	26.08	270.03
m114	0.1	1.0	15.0	3.0	0.0	9.3	0.0	40.1	9.3	22.2	0.0	0.0	267.7
m494	0.0	3.3	27.1	4.3	0.3	10.6	0.6	23.6	10.7	16.2	0.0	3.4	266.2
m257	0.5	0.5	0.4	0.1	0.0	3.1	0.0	35.0	0.0	29.2	25.6	5.7	263.8
m279	0.5	0.3	4.4	0.0	0.0	4.0	0.6	32.2	7.3	26.8	23.8	0.0	262.1
m414	0.0	3.9	26.8	4.4	0.3	11.1	0.0	28.8	4.2	20.5	0.0	0.0	258.9
m286	0.3	0.0	0.7	0.0	0.0	2.7	0.0	18.4	0.0	62.3	15.5	0.0	253.4
m417	0.7	3.5	23.8	4.2	0.3	9.4	0.6	25.5	7.4	19.9	4.7	0.0	245.4
CvFatB1	0.00	1.22	24.16	21.59	1.48	4.84	3.89	21.10	0.21	10.17	5.60	5.74	243.03
m614	0.2	3.3	19.7	4.5	0.2	9.8	0.0	30.1	3.5	27.9	0.8	0.0	240.7
m114	0.0	0.3	1.9	0.2	0.0	3.1	0.0	27.3	1.1	32.9	24.1	9.2	239.8
m494	0.6	0.5	0.7	0.3	0.0	1.8	0.0	35.1	0.4	36.8	19.4	4.5	237.6
m257	0.5	2.1	13.2	3.2	0.0	10.4	0.0	35.0	4.0	31.6	0.0	0.0	231.2
m279	1.2	2.8	16.6	4.6	0.1	10.2	0.0	25.4	6.8	32.3	0.0	0.0	225.2
m414	0.6	0.8	3.5	0.9	0.0	4.9	1.4	23.3	4.1	37.3	23.1	0.0	223.6
m286	1.1	0.0	1.6	0.0	0.0	3.1	0.0	16.2	0.0	58.0	20.0	0.0	216.4
m417	0.2	0.6	13.3	6.2	0.0	10.5	5.6	13.9	4.6	35.6	9.4	0.0	216.0
m272	0.5	0.0	5.2	1.7	0.0	5.4	0.0	37.9	6.1	39.6	3.7	0.0	213.8
m209	0.8	0.8	1.0	0.0	0.0	1.5	0.0	26.1	0.0	46.9	23.0	0.0	211.7
m284	0.0	0.3	4.8	0.0	0.0	4.4	0.0	28.4	3.3	58.8	0.0	0.0	201.5
m252	0.7	0.4	2.4	0.4	0.0	2.1	0.0	13.3	0.0	58.9	11.4	10.5	200.6
m143	1.3	1.1	5.9	0.9	0.0	7.9	0.0	22.5	2.8	46.8	2.7	8.0	197.2
m185	0.3	0.4	3.4	1.0	0.0	4.7	0.3	29.9	5.8	39.8	14.2	0.0	195.0
m135	1.3	3.7	25.9	3.7	0.1	11.5	0.0	24.3	8.3	20.4	0.7	0.0	192.1
m137	0.9	1.1	7.0	0.6	0.1	4.9	0.3	30.6	4.8	26.1	19.7	3.8	188.9
m291	1.4	0.0	7.1	4.6	0.0	9.1	0.0	19.9	1.0	53.0	3.9	0.0	187.3
m281	3.8	5.0	12.9	1.1	0.0	3.2	0.4	24.6	0.9	45.1	3.0	0.0	185.8
m268	1.5	0.7	4.1	1.6	0.0	7.1	0.0	34.5	5.9	44.6	0.0	0.0	182.3
m553	0.2	2.4	16.5	3.7	0.2	8.8	0.0	34.1	4.1	28.7	1.3	0.0	181.6
m266	1.0	0.0	3.4	1.3	0.0	6.3	0.0	37.4	8.2	42.4	0.0	0.0	180.5
m132	1.1	0.7	1.8	0.2	0.0	3.3	0.0	17.7	0.7	57.1	11.9	5.4	178.8
m402	0.8	0.0	0.6	0.6	0.0	4.1	0.0	18.6	1.9	52.0	21.4	0.0	175.0
m303	1.6	0.4	11.1	4.7	0.0	20.8	2.7	23.1	3.5	32.2	0.0	0.0	171.5
m404	0.8	0.4	4.0	1.6	0.0	11.3	0.0	29.4	3.7	45.0	3.8	0.0	171.2
m554	0.0	0.7	2.6	0.8	0.0	4.1	0.0	30.2	1.3	50.5	9.8	0.0	163.7
m138	1.1	0.7	2.3	0.2	0.0	3.5	0.0	26.8	1.3	38.2	20.4	5.4	163.3
m568	0.6	1.0	4.0	1.1	0.0	5.2	0.0	37.7	0.0	36.8	13.6	0.0	161.6
m220	1.9	0.3	1.4	0.0	0.0	2.8	0.0	39.1	1.1	42.1	11.2	0.0	159.9
m107	0.0	0.8	9.3	1.7	0.0	14.5	0.8	31.0	6.6	29.2	3.0	3.0	156.9
m116	0.0	0.4	1.7	0.1	0.0	2.1	0.0	24.6	0.0	36.7	23.9	10.5	156.5
m516	0.2	1.4	12.9	1.9	0.0	9.2	0.0	24.9	4.7	32.9	3.4	8.6	155.8
m581	0.0	2.1	24.1	4.4	0.0	10.6	0.0	26.9	2.6	28.1	1.2	0.0	154.2
m255	0.7	0.7	3.8	1.0	0.0	6.2	0.0	27.6	1.9	58.1	0.0	0.0	151.8
m159	0.0	0.9	9.4	1.7	0.0	8.9	0.0	37.2	7.8	34.2	0.0	0.0	151.2
m145	2.3	1.0	4.0	1.0	0.0	8.6	0.0	14.5	2.1	66.6	0.0	0.0	150.3
m292	1.0	0.4	2.8	0.3	0.0	14.6	0.0	23.0	0.0	56.3	1.7	0.0	146.3
m410	1.3	1.5	2.3	0.5	0.0	4.9	0.0	21.6	0.0	56.8	11.1	0.0	145.0
m247	0.8	0.0	0.4	0.0	0.0	3.0	0.0	10.9	0.0	84.9	0.0	0.0	141.4
m295	1.7	0.7	3.2	0.6	0.0	3.5	0.0	26.2	1.6	58.4	4.1	0.0	139.4
m109	0.0	1.1	7.4	1.0	0.0	6.6	0.3	31.9	3.7	31.2	16.8	0.0	138.7
m429	1.3	1.2	3.0	0.0	0.0	5.1	0.0	34.4	2.9	52.0	0.0	0.0	135.1
m564	0.0	2.8	17.9	3.8	0.3	8.3	0.0	32.9	3.1	30.8	0.0	0.0	134.2
m259	1.3	0.0	10.5	1.9	0.0	5.1	0.0	15.2	0.0	66.0	0.0	0.0	127.3
m140	1.6	0.7	4.1	0.6	0.0	6.2	0.0	29.3	2.8	38.5	7.7	8.5	126.9
m405	1.4	0.9	2.3	0.3	0.0	5.0	0.0	29.0	0.0	53.6	7.4	0.0	126.4
m273	0.0	0.0	3.5	0.5	0.0	3.7	0.0	23.3	2.2	66.9	0.0	0.0	125.3
m289	0.0	0.3	6.6	1.4	0.0	4.7	1.0	21.6	1.3	58.9	4.1	0.0	124.4
m406	1.6	0.6	2.2	0.0	0.0	5.2	0.0	31.5	0.0	47.7	11.2	0.0	123.9
m278	0.0	0.0	0.0	0.0	0.0	4.4	0.0	12.1	0.0	83.5	0.0	0.0	123.8
m545	1.3	3.3	19.3	3.5	0.0	9.0	0.0	26.9	4.1	32.5	0.0	0.0	122.9
m332	0.0	0.0	1.8	0.3	0.0	5.8	0.0	15.3	0.0	76.7	0.0	0.0	120.7
m450	0.3	1.0	4.0	0.7	0.0	7.3	0.0	29.8	3.8	45.4	7.6	0.0	119.9
m276	1.6	0.0	2.5	0.0	0.0	2.5	0.0	21.1	0.0	72.3	0.0	0.0	119.7
m531	2.0	1.9	8.7	2.0	0.0	8.1	0.0	27.9	4.7	43.1	0.9	0.7	119.2
m280	1.3	0.0	0.7	0.0	0.0	5.2	0.0	27.1	0.5	65.3	0.0	0.0	119.1
m125	1.5	1.3	4.7	0.7	0.0	6.6	0.0	31.6	2.9	38.9	11.8	0.0	114.3
m127	2.6	1.2	6.4	1.7	0.0	12.9	1.2	28.8	2.3	39.7	3.4	0.0	113.3
m260	3.3	0.0	5.2	0.0	0.0	5.3	0.0	10.3	0.0	75.9	0.0	0.0	112.3
m235	14.6	5.5	17.1	1.6	0.0	5.7	0.0	18.3	0.9	36.3	0.0	0.0	111.9
m338	0.0	0.0	1.4	0.2	0.0	6.1	0.0	8.5	0.0	83.8	0.0	0.0	110.5

m534	1.2	1.6	2.9	0.8	0.0	5.2	0.0	9.7	0.0	71.8	1.7	5.1	108.9
m407	0.3	1.6	3.0	0.7	0.0	7.1	0.0	37.1	4.6	45.6	0.0	0.0	108.7
m500	0.2	1.8	5.7	1.8	0.0	11.2	0.0	25.2	3.2	50.8	0.0	0.0	108.4
m347	0.0	0.0	2.4	0.3	0.0	3.9	0.0	14.1	0.0	79.3	0.0	0.0	108.1
m535	1.1	1.6	1.7	0.7	0.0	4.5	0.0	8.0	0.0	72.9	0.0	9.5	107.8
m134	1.8	1.4	8.1	1.2	0.0	7.2	0.5	33.6	5.9	37.4	2.9	0.0	107.8
m323	0.0	0.0	1.9	0.3	0.0	4.3	0.0	13.0	0.0	80.4	0.0	0.0	107.3
m253	1.2	0.0	0.8	0.0	0.0	1.1	0.0	14.0	0.0	78.5	4.3	0.0	106.7
CnFatB3	0.00	0.12	6.02	0.00	0.00	34.29	2.28	26.17	5.71	12.17	3.96	9.27	102.74
m115	0.0	0.6	2.2	0.3	0.0	5.1	0.0	18.8	0.0	55.5	12.5	5.0	101.8
m131	2.7	1.8	5.6	1.5	0.0	8.8	0.0	23.2	3.3	53.0	0.0	0.0	100.5
m549	1.0	1.3	5.8	1.9	0.0	6.9	0.0	30.3	0.0	50.5	2.3	0.0	97.6
m497	0.0	1.4	2.9	0.7	0.0	7.3	0.0	5.0	0.0	82.7	0.0	0.0	91.1
m533	0.9	2.3	7.3	1.8	0.0	6.3	0.0	30.4	5.2	45.1	0.0	0.8	90.9
m513	0.5	2.2	7.7	1.7	0.0	7.9	0.0	25.9	5.1	49.0	0.0	0.0	90.3
m307	0.5	0.0	0.0	0.0	0.0	2.7	0.0	6.4	0.0	90.3	0.0	0.0	90.3
m468	0.0	1.8	5.2	0.0	0.0	5.2	0.0	28.3	3.5	56.0	0.0	0.0	88.3
m496	0.3	1.4	4.6	1.1	0.0	7.5	0.0	19.9	3.0	62.2	0.0	0.0	87.7
m470	1.2	1.3	8.9	0.7	0.0	9.4	0.0	29.2	4.9	44.3	0.0	0.0	87.6
m274	0.9	0.0	1.8	0.0	0.0	3.3	0.0	19.0	0.0	74.9	0.0	0.0	87.2
m277	0.0	0.0	0.7	0.0	0.0	1.5	0.0	17.2	0.6	80.0	0.0	0.0	87.2
m612	0.0	2.9	6.7	2.1	0.0	8.3	0.0	25.3	1.5	53.2	0.0	0.0	86.9
m121	0.0	1.3	7.9	0.6	0.0	5.3	0.0	26.4	0.8	43.4	14.4	0.0	86.9
m221	1.6	0.6	1.4	0.0	0.0	1.6	0.0	24.0	0.0	70.7	0.0	0.0	85.4
m461	1.4	1.5	3.2	0.0	0.0	7.3	0.0	20.1	2.4	64.1	0.0	0.0	82.2
m583	1.0	2.9	18.0	3.2	0.0	2.3	0.0	23.1	1.5	48.1	0.0	0.0	79.4
m488	1.2	1.7	7.3	0.0	0.0	6.4	0.0	31.2	8.7	43.4	0.0	0.0	79.4
m485	0.0	0.9	5.6	1.1	0.0	9.4	0.0	26.1	4.1	52.8	0.0	0.0	79.3
m465	1.5	1.7	1.2	0.4	0.0	7.1	0.0	9.2	0.0	78.9	0.0	0.0	77.4
m117	3.9	1.2	2.5	0.3	0.0	4.8	0.0	15.6	0.0	60.0	11.6	0.0	75.7
m126	0.0	0.8	3.5	1.2	0.0	5.5	0.0	29.2	0.0	53.2	6.6	0.0	74.8
m299	1.8	0.6	0.5	0.0	0.0	0.0	0.0	26.3	0.0	61.2	9.6	0.0	73.7
m231	3.5	0.0	0.0	0.0	0.0	2.2	0.0	36.0	0.0	58.3	0.0	0.0	73.3
m572	0.0	2.4	7.2	1.9	0.0	6.5	0.0	25.5	3.4	53.1	0.0	0.0	72.5
m451	2.2	1.9	2.8	0.0	0.0	6.0	0.0	14.2	0.0	72.8	0.0	0.0	71.5
m555	1.4	1.7	2.8	0.5	0.0	4.1	0.0	14.5	0.0	71.5	3.4	0.0	71.4
m508	0.4	2.1	7.9	1.8	0.0	8.4	0.0	23.8	4.2	51.3	0.0	0.0	71.0
m550	0.6	1.6	3.7	0.9	0.0	6.2	0.0	14.6	1.0	71.3	0.0	0.0	70.6
m602	1.1	2.2	4.9	1.3	0.0	0.0	0.0	21.7	0.0	68.8	0.0	0.0	69.1
m403	0.9	2.1	2.9	0.9	0.0	0.0	0.0	10.9	0.0	82.3	0.0	0.0	69.1
m298	0.0	0.0	0.4	0.0	0.0	0.0	0.0	21.6	0.0	72.9	5.1	0.0	69.1
m120	3.4	2.1	8.9	2.2	0.0	9.1	0.0	24.4	4.5	45.5	0.0	0.0	68.7
m464	0.0	2.4	3.1	0.0	0.0	3.4	0.0	9.8	0.0	81.2	0.0	0.0	68.3
m603	0.9	2.5	0.0	1.2	0.0	5.2	0.0	11.0	0.0	77.4	1.8	0.0	67.9
m324	0.0	0.0	0.0	0.2	0.0	1.8	0.0	11.3	0.0	86.7	0.0	0.0	67.8
m628	0.0	3.6	9.1	2.4	0.0	0.0	0.0	18.1	0.0	66.7	0.0	0.0	67.1
m577	2.1	1.9	3.6	1.4	0.0	9.4	0.0	12.8	0.0	68.9	0.0	0.0	67.0
m590	0.0	0.0	4.4	1.8	0.0	7.7	0.0	29.6	1.2	54.0	1.3	0.0	65.8
m297	0.0	0.6	0.0	0.0	0.0	0.0	0.0	17.4	0.0	77.8	4.2	0.0	65.5
m463	0.0	1.7	3.8	0.0	0.0	4.4	0.0	8.9	0.0	81.2	0.0	0.0	65.4
m576	0.0	2.3	6.8	2.0	0.0	0.0	0.0	31.5	2.3	55.1	0.0	0.0	65.1
m522	0.4	2.0	4.2	0.8	0.0	5.8	0.0	13.2	0.0	70.3	3.2	0.0	64.6
m579	0.8	1.2	1.9	0.8	0.0	0.0	0.0	11.0	0.0	84.4	0.0	0.0	64.1
m222	3.5	0.0	1.0	0.0	0.0	3.0	0.0	17.9	0.0	74.6	0.0	0.0	64.0
m562	0.0	0.0	4.4	1.4	0.0	4.4	0.0	19.8	1.3	68.8	0.0	0.0	63.8
m622	0.0	2.5	5.4	1.6	0.0	2.6	0.0	22.4	2.1	63.5	0.0	0.0	62.7
m510	0.0	2.2	5.1	0.0	0.0	5.3	0.0	14.4	0.0	73.0	0.0	0.0	62.5
m483	1.5	1.3	0.0	0.8	0.0	6.5	0.0	13.7	0.0	76.2	0.0	0.0	62.1
m625	0.0	1.9	5.0	1.3	0.0	0.5	0.0	24.1	0.0	67.2	0.0	0.0	61.6
m558	0.2	0.0	2.2	0.6	0.0	5.8	0.0	8.2	0.0	82.9	0.0	0.0	61.4
m459	2.1	0.0	1.9	0.0	0.0	4.7	0.0	9.5	0.0	81.9	0.0	0.0	60.7
m584	0.3	0.0	3.0	0.7	0.0	1.1	0.0	10.9	0.0	84.1	0.0	0.0	59.5
m619	0.0	2.5	5.6	2.2	0.0	5.4	0.0	19.0	0.0	65.4	0.0	0.0	59.5
m453	2.7	0.8	2.7	0.0	0.0	7.4	0.0	10.9	0.0	75.5	0.0	0.0	59.4
m340	0.0	0.9	1.8	0.0	0.0	1.7	0.0	17.6	0.0	77.9	0.0	0.0	59.2
m228	4.4	0.0	0.4	0.0	0.0	0.0	0.0	14.7	0.0	80.4	0.0	0.0	58.8
m227	2.2	0.7	1.0	0.0	0.0	1.8	0.0	13.2	0.0	81.0	0.0	0.0	58.8
m536	1.4	2.7	3.6	0.7	0.0	5.7	0.0	8.5	0.0	77.4	0.0	0.0	58.4
m523	2.1	1.5	3.4	1.2	0.0	5.9	0.0	12.1	0.0	72.0	1.7	0.0	55.9
m224	2.0	0.0	1.1	0.0	0.0	2.3	0.0	14.4	0.0	80.2	0.0	0.0	55.4
m537	0.8	1.6	2.8	0.7	0.0	3.3	0.0	8.4	0.0	82.5	0.0	0.0	55.4
m457	1.0	2.4	1.1	0.0	0.0	3.3	0.0	9.2	0.0	83.0	0.0	0.0	55.4
m597	0.5	3.2	7.4	2.1	0.0	0.0	0.0	31.1	3.1	52.6	0.0	0.0	55.2
m524	0.7	1.7	3.2	1.0	0.0	5.5	0.0	8.4	0.0	79.5	0.0	0.0	54.9

m557	0.0	3.7	1.4	0.5	0.0	5.0	0.0	7.9	0.0	81.5	0.0	0.0	54.8
m606	0.9	2.0	2.3	0.0	0.0	0.0	0.0	10.1	0.0	84.7	0.0	0.0	54.2
m578	0.0	2.0	3.3	0.7	0.0	0.0	0.0	9.7	0.0	84.4	0.0	0.0	53.6
m452	0.0	2.1	2.9	0.0	0.0	0.0	0.0	12.1	0.0	82.8	0.0	0.0	53.3
m539	0.0	1.0	2.6	1.1	0.0	2.8	0.0	8.8	0.0	83.6	0.0	0.0	53.2
m223	2.0	1.1	0.7	0.0	0.0	2.6	0.0	18.7	0.0	74.7	0.0	0.0	53.0
m631	0.0	2.7	6.5	2.3	0.0	0.0	0.0	21.3	1.4	65.7	0.0	0.0	52.7
m456	2.4	2.0	2.0	0.0	0.0	2.3	0.0	2.0	0.0	89.3	0.0	0.0	52.2
m462	0.0	1.6	4.1	0.0	0.0	4.6	0.0	13.2	0.7	75.8	0.0	0.0	52.2
m626	0.0	1.5	2.7	0.8	0.0	0.0	0.0	13.7	0.0	81.3	0.0	0.0	51.8
m598	0.0	2.5	3.6	1.0	0.0	2.2	0.0	14.5	0.0	76.3	0.0	0.0	51.3
m604	1.5	2.7	0.0	0.5	0.0	0.0	0.0	13.7	0.0	81.5	0.0	0.0	51.1
m585	0.9	0.0	1.9	0.5	0.0	1.4	0.0	9.7	0.0	85.6	0.0	0.0	50.6
m627	0.0	2.1	1.8	0.6	0.0	0.0	0.0	11.3	0.0	84.2	0.0	0.0	50.1
m225	1.9	0.8	0.7	0.0	0.0	0.0	0.0	15.4	0.0	81.2	0.0	0.0	48.2
m613	0.0	1.8	4.1	0.6	0.0	0.0	0.0	15.5	0.0	78.0	0.0	0.0	47.7
m454	2.8	0.0	1.2	0.0	0.0	0.0	0.0	8.0	0.0	88.0	0.0	0.0	47.4
m560	0.0	3.0	2.0	0.6	0.0	3.7	0.0	7.9	0.0	82.8	0.0	0.0	47.2
m573	3.2	3.1	4.9	1.0	0.0	0.0	0.0	15.3	0.0	72.4	0.0	0.0	47.1
m458	0.9	2.4	2.3	0.0	0.0	2.9	0.0	4.5	0.0	87.0	0.0	0.0	46.7
m226	2.3	1.1	1.0	0.0	0.0	0.0	0.0	14.0	0.0	81.6	0.0	0.0	46.1
m232	2.6	0.0	0.8	0.0	0.0	0.0	0.0	29.5	0.0	67.0	0.0	0.0	46.1
m233	1.5	0.0	0.7	0.0	0.0	2.5	0.0	18.4	0.0	76.9	0.0	0.0	45.9
m559	0.0	0.0	2.2	0.5	0.0	5.5	0.0	9.1	0.0	82.8	0.0	0.0	45.4
m234	2.3	0.0	1.5	0.0	0.0	0.0	0.0	15.6	0.0	80.6	0.0	0.0	45.3
m591	0.5	0.0	3.8	1.2	0.0	0.0	0.0	17.2	0.0	77.3	0.0	0.0	44.9
m101	0.9	1.9	0.0	0.4	0.0	6.3	0.0	13.3	0.0	77.2	0.0	0.0	44.8
m620	0.0	1.3	1.4	0.9	0.0	0.0	0.0	10.2	0.0	86.1	0.0	0.0	44.5
m605	0.6	2.6	0.0	0.0	0.0	0.0	0.0	11.7	0.0	85.1	0.0	0.0	44.3
m511	0.7	2.8	3.9	0.0	0.0	3.9	0.0	11.0	0.0	77.8	0.0	0.0	43.9
m538	0.9	3.0	3.5	0.8	0.0	3.7	0.0	8.9	0.0	79.2	0.0	0.0	43.8
m632	0.0	2.5	1.9	0.8	0.0	0.0	0.0	8.8	0.0	85.9	0.0	0.0	43.8
m599	0.6	2.2	6.2	0.9	0.0	0.0	0.0	12.7	0.0	77.4	0.0	0.0	42.1
m582	0.3	3.9	5.0	1.9	0.0	0.0	0.0	17.3	0.0	71.5	0.0	0.0	41.7
m489	0.7	2.6	5.2	1.3	0.0	6.1	0.0	20.9	0.0	63.2	0.0	0.0	41.7
m239	2.8	0.0	0.7	0.0	0.0	0.0	0.0	12.4	0.0	84.1	0.0	0.0	41.5
m432	3.9	3.1	6.0	0.0	0.0	11.6	0.0	33.1	0.0	42.3	0.0	0.0	41.2
m236	1.6	0.0	2.1	0.0	0.0	1.2	0.0	15.5	0.0	79.6	0.0	0.0	40.9
m615	0.0	2.3	6.6	1.2	0.0	0.0	0.0	16.1	0.0	73.9	0.0	0.0	40.4
m229	3.0	1.1	1.3	0.0	0.0	0.0	0.0	12.4	0.0	82.2	0.0	0.0	40.3
m238	2.4	1.5	0.9	0.0	0.0	1.6	0.0	9.8	0.0	83.7	0.0	0.0	39.7
m237	3.3	0.0	0.8	0.0	0.0	0.0	0.0	14.4	0.0	81.6	0.0	0.0	38.2
m616	0.0	3.0	6.3	1.3	0.0	3.3	0.0	12.2	0.0	73.9	0.0	0.0	37.9
m244	3.4	1.7	0.9	0.0	0.0	0.0	0.0	10.2	0.0	83.8	0.0	0.0	36.1
m241	2.2	0.0	0.8	0.0	0.0	0.0	0.0	12.9	0.0	84.1	0.0	0.0	35.3
m240	3.7	0.0	1.3	0.0	0.0	0.0	0.0	13.2	0.0	81.8	0.0	0.0	34.3
m242	3.6	0.0	0.0	0.0	0.0	0.0	0.0	15.1	0.0	81.3	0.0	0.0	31.5
m243	4.7	0.0	1.6	0.0	0.0	0.0	0.0	11.5	0.0	82.2	0.0	0.0	29.6

[a] The intensity of the green shading of each cell is proportional to the mol % of each fatty acid.

[b] The length of each red-bar in each cell is proportional to the fatty acid productivity

Supplemental Table 3. Identification of acyl-ACP TE variants expressing highest fatty acid productivity

192 colonies identified by enhanced Neutral Red staining were cultured and their fatty acid productivity and profiles were determined, and in parallel the acyl-ACP TE sequences were determined. From 176 successfully recovered sequences identified 26 unique acyl-ACP TE variants.

Colony number	Fatty acid profiles (mol %) [a]												Fatty acid productivity (μM) [b]	Acyl-ACP TE variant
	C4:0	C6:0	C8:0	C10:0	C10:1	C12:0	C12:1	C14:0	C14:1	C16:0	C16:1	C18:1		
m162	0.5	4.9	38.5	5.3	0.5	11.7	2.2	19.4	11.6	3.1	0.6	1.5	1695.0	TEGm162
m261	0.3	3.3	25.1	5.7	0.6	12.9	1.2	32.7	10.9	4.7	0.7	2.0	1642.4	TEGm162
m250	1.6	1.7	20.0	4.3	0.7	3.8	2.0	19.8	3.5	19.1	17.1	6.2	1618.3	TEGm250
m265	0.2	2.4	25.3	5.4	0.6	13.7	1.1	31.1	11.9	6.0	1.0	1.4	1610.2	TEGm162
m312	0.2	3.1	25.7	4.1	0.1	9.2	1.0	32.7	14.6	5.4	1.4	2.4	1578.3	TEGm162
m258	4.4	6.4	50.4	6.4	1.1	4.7	1.8	9.1	1.3	10.2	1.9	2.3	1557.8	TEGm258
m170	0.4	4.9	30.7	6.0	0.6	13.0	2.3	21.4	13.2	3.1	1.6	2.7	1552.7	TEGm162
m313	0.3	3.7	27.2	4.6	0.3	10.1	1.0	32.1	13.0	3.8	1.2	2.8	1532.6	TEGm162
m172	0.2	4.0	24.1	4.3	0.4	10.2	2.1	23.7	13.4	6.5	8.0	3.3	1504.2	TEGm162
m310	0.3	3.1	25.7	4.2	0.0	8.9	0.8	33.9	14.0	4.8	1.3	3.0	1489.9	TEGm162
m163	0.4	5.0	28.2	5.9	0.6	12.6	1.7	25.8	14.9	3.4	1.6	0.0	1486.2	TEGm162
m320	0.2	2.9	24.7	3.9	0.0	9.4	0.7	38.1	15.2	4.9	0.0	0.0	1483.0	TEGm162
m314	0.2	2.9	23.2	4.8	0.3	10.8	1.0	31.5	13.0	10.4	0.0	1.9	1444.9	TEGm162
m503	0.2	4.4	31.7	6.6	0.7	14.2	1.2	25.7	6.8	3.8	1.4	3.3	1444.1	TEGm162
m174	0.1	3.8	24.1	4.2	0.4	9.2	1.5	25.6	12.8	6.7	8.6	3.0	1433.8	TEGm162
m322	0.3	2.9	24.2	4.2	0.0	9.0	0.7	39.7	15.9	3.1	0.0	0.0	1428.6	TEGm162
m547	0.2	4.1	34.8	5.7	0.6	13.1	1.0	21.7	11.2	4.0	1.4	2.2	1427.7	TEGm162
m169	0.4	4.2	33.7	4.6	0.6	11.7	2.8	18.8	14.6	3.8	1.9	2.7	1426.9	TEGm169
m317	0.3	3.1	25.1	4.4	0.0	9.4	0.9	37.3	15.4	3.1	1.1	0.0	1422.5	TEGm162
m530	0.3	4.2	34.5	6.8	0.8	15.0	0.2	24.8	5.7	3.1	1.6	3.0	1411.8	TEGm162
m263	0.3	3.1	26.5	6.2	0.5	13.7	1.1	31.2	10.6	4.4	0.8	1.5	1392.5	TEGm162
m419	0.1	0.2	13.2	7.6	2.0	13.7	12.2	15.2	8.0	8.9	15.2	3.6	1391.8	TEGm419
m532	0.2	4.4	36.6	5.5	0.6	13.9	0.8	27.1	4.6	3.4	1.2	1.7	1377.1	TEGm162
m525	0.3	4.1	32.6	6.5	0.9	15.5	0.8	22.3	9.2	3.6	1.3	3.1	1367.6	TEGm162
m311	0.3	3.3	25.2	4.8	0.0	10.1	1.0	33.9	14.6	4.8	0.9	1.0	1364.4	TEGm162
m283	0.3	2.7	27.5	6.4	0.4	12.4	0.8	30.1	11.3	6.4	0.5	1.2	1361.4	TEGm162
m505	0.3	4.2	30.8	6.0	0.7	13.3	0.9	25.7	7.9	4.1	1.5	4.7	1359.9	TEGm162
m499	0.3	4.2	31.8	6.5	0.7	15.4	0.7	27.2	6.1	3.0	1.3	2.9	1349.8	TEGm162
m514	0.3	4.0	33.3	6.8	0.8	13.8	1.1	24.8	5.6	3.2	1.4	4.9	1346.1	TEGm162
m543	0.3	3.9	33.4	5.6	0.6	13.2	0.9	24.3	11.3	3.0	1.2	2.5	1342.0	TEGm162
m164	0.5	5.6	29.4	6.5	0.6	11.9	1.9	22.8	13.0	3.2	1.6	3.0	1339.7	TEGm162
m179	0.5	5.3	33.4	5.5	0.6	12.0	2.0	18.4	13.8	3.6	2.1	2.7	1337.5	TEGm162
m552	0.2	3.7	32.4	6.0	0.7	15.2	0.8	21.7	10.0	5.6	1.2	2.5	1325.6	TEGm162
m175	0.1	3.6	23.7	4.0	0.4	10.2	1.8	28.2	15.4	5.0	4.3	3.3	1317.2	TEGm162
m526	0.3	3.9	31.7	6.3	0.8	14.2	0.7	25.5	9.4	3.2	1.2	2.8	1313.2	TEGm162
m566	0.1	3.7	33.8	5.8	0.6	13.6	1.0	24.3	9.6	3.8	1.0	2.6	1313.2	TEGm162
m593	0.0	3.5	37.1	5.8	0.6	13.9	0.0	21.1	11.3	3.2	1.2	2.4	1312.8	TEGm162
m161	0.6	6.3	35.7	5.9	0.6	11.9	2.4	18.5	10.9	2.8	1.8	2.7	1312.0	TEGm162
m264	0.3	3.1	24.7	4.8	0.4	13.4	0.8	33.5	11.6	4.7	1.2	1.6	1309.0	TEGm162
m512	0.1	4.2	36.3	6.4	0.7	13.6	0.7	26.3	6.0	3.0	1.1	1.5	1306.8	TEGm162
m504	0.3	4.6	34.1	6.4	0.7	13.2	0.9	26.3	7.3	2.4	1.4	2.4	1305.6	sequencing failed
m316	0.1	3.0	24.1	4.5	0.0	10.1	0.7	36.5	15.8	5.2	0.0	0.0	1300.1	TEGm162
m515	0.3	5.1	34.6	7.2	0.8	15.0	1.1	24.3	5.2	2.5	1.4	2.3	1298.9	TEGm162
m267	0.1	2.8	27.7	5.4	0.5	12.7	1.2	31.8	11.0	5.0	0.8	0.9	1293.9	TEGm162
m271	0.3	3.4	27.9	6.1	0.5	14.9	0.9	27.3	10.2	6.7	0.6	1.2	1292.5	TEGm162
m214	0.2	0.2	1.6	0.1	0.0	2.0	0.4	36.1	1.8	18.6	35.5	3.5	1290.4	sequencing failed
m594	0.1	3.0	29.3	5.4	0.5	12.8	0.8	26.3	14.7	3.3	1.6	2.1	1287.1	TEGm162
m474	0.2	3.5	28.5	5.2	0.5	12.6	0.9	29.8	8.9	4.8	1.2	3.8	1283.5	TEGm162
m321	0.1	3.0	25.4	4.2	0.0	9.4	0.7	36.4	16.6	4.2	0.0	0.0	1280.7	TEGm162
m330	0.3	3.2	24.8	4.3	0.0	9.0	0.5	36.7	15.7	5.7	0.0	0.0	1280.2	TEGm162
m506	0.3	4.4	32.3	6.5	0.7	14.5	0.8	29.3	5.8	3.7	1.8	0.0	1276.2	TEGm162
m472	0.1	3.2	26.9	5.3	0.6	13.1	1.0	29.3	9.2	5.7	1.2	4.4	1274.2	TEGm162
m166	0.4	4.7	30.2	5.4	0.6	11.9	2.4	21.8	13.6	3.6	2.0	3.4	1271.9	TEGm162
m487	0.1	3.5	28.2	5.6	0.6	13.7	0.8	30.2	7.7	4.0	1.4	4.3	1266.1	TEGm162
m529	0.3	4.5	32.5	6.3	0.7	9.7	1.2	31.2	6.6	3.3	1.4	2.3	1264.2	TEGm162
m469	0.2	3.3	27.8	5.4	0.6	13.8	1.0	27.7	9.0	6.0	1.3	3.8	1264.0	TEGm162
m595	0.2	3.5	32.4	6.6	0.8	14.7	1.2	20.5	13.1	3.3	1.3	2.3	1261.2	TEGm162
m344	0.2	2.9	24.3	3.9	0.0	9.8	0.6	37.1	12.4	8.7	0.0	0.0	1259.9	TEGm162
m563	0.1	3.8	34.6	5.9	0.7	13.8	1.0	21.5	11.8	3.8	1.1	2.0	1258.5	TEGm162
m293	0.3	2.7	26.3	6.1	0.5	14.8	1.0	30.4	9.8	5.5	1.0	1.8	1258.0	TEGm162
m288	0.2	0.3	30.9	5.5	2.2	9.8	8.5	12.9	7.1	12.1	8.3	2.2	1256.4	TEGm288
m567	0.1	3.6	34.7	6.0	0.7	13.3	0.9	23.3	10.0	3.0	1.6	2.6	1248.1	TEGm162
m440	0.2	3.0	25.5	4.8	0.6	12.3	1.0	32.0	10.3	5.0	1.0	4.2	1246.5	TEGm162
m154	0.6	5.7	33.2	6.1	0.7	12.0	2.7	18.4	13.4	2.7	1.7	2.8	1244.0	TEGm162
m466	0.1	3.7	31.8	5.8	0.6	13.7	1.0	26.0	10.1	2.8	1.2	3.4	1242.9	TEGm162
m309	0.2	2.3	20.4	3.8	0.0	7.7	0.6	44.2	16.9	3.1	0.7	0.0	1239.3	TEGm162
m183	0.4	0.5	12.7	4.2	0.8	9.2	6.6	23.2	11.7	10.0	18.4	2.4	1235.8	TEGm183
m491	0.2	3.6	30.2	5.5	0.6	13.2	1.0	28.7	7.8	3.4	1.9	3.9	1228.6	TEGm162
m540	0.2	3.9	34.2	5.9	0.8	13.2	1.1	22.9	9.7	5.1	1.0	2.0	1226.5	TEGm162
m173	0.3	0.3	2.8	0.4	0.0	3.5	0.5	35.9	3.1	12.0	36.2	4.9	1225.8	TEGm173
m542	0.2	3.3	30.8	5.8	0.6	12.9	0.8	26.5	12.5	3.3	1.0	2.3	1219.1	TEGm162
m471	0.2	3.6	28.6	5.3	0.6	13.5	1.0	26.8	8.8	5.8	1.5	4.1	1214.0	TEGm162
m467	0.2	3.2	28.7	5.4	0.5	12.9	0.8	29.2	9.9	3.8	1.3	4.1	1212.3	TEGm162
m541	0.2	3.8	32.9	6.2	0.8	13.2	0.5	23.2	11.7	3.5	1.2	2.8	1208.4	TEGm162
m569	0.2	3.9	36.9	6.3	0.8	14.1	1.2	22.7	9.2	3.2	1.5	0.0	1204.4	TEGm162
m204	0.1	0.2	1.3	0.1	0.0	1.6	0.3	38.0	1.5	19.8	33.6	3.4	1202.6	TEGm204
m634	0.2	4.2	38.7	6.3	0.8	13.8	0.6	16.7	13.5	2.8	1.0	1.5	1197.2	TEGm162
m486	0.2	3.7	27.2	6.0	0.7	14.3	0.6	29.2	8.3	4.2	1.5	4.2	1192.6	TEGm162
m343	0.3	2.6	23.1	3.8	0.0	9.0	0.7	39.6	14.5	6.4	0.0	0.0	1190.0	TEGm162
m548	0.2	3.6	33.4	6.5	0.8	14.9	1.2	25.3	6.7	3.7	1.3	2.5	1184.9	TEGm162
m341	0.3	3.0	24.8	4.2	0.0	10.0	0.6	36.6	13.8	6.7	0.0	0.0	1183.4	TEGm162
m436	0.2	3.1	26.3	5.5	0.6	13.5	1.0	29.8	10.7	4.7	1.0	3.5	1183.3	TEGm162
m315	0.3	3.4	26.1	5.5	0.4	12.1	1.5	26.6	14.0	5.1	1.6	3.5	1183.0	TEGm162
m269	0.4	3.4	27.6	5.9	0.4	13.0	1.1	29.2	11.8	5.4	0.6	1.2	1180.6	TEGm162
m490	0.3	4.4	33.3	6.1	0.6	13.5	0.9	25.8	7.7	3.5	1.2	2.7	1178.9	TEGm162
m575	0.0	3.7	34.8	6.1	0.7	13.4	1.3	23.0	11.4	3.7	1.5	0.5	1178.1	TEGm162
m589	0.0	3.7	37.7	6.1	0.7	13.9	0.0	21.8	9.6	3.4	1.1	2.0	1173.9	TEGm162
m608	0.1	3.8	38.7	5.8	0.6	14.0	0.5	20.0	10.5	3.6	0.6	1.7	1173.3	TEGm162

m446	0.3	3.6	27.1	5.6	0.0	13.5	1.3	28.5	10.1	4.0	1.2	4.8	1170.9	TEGm162
m610	0.0	4.0	35.0	6.0	0.7	13.7	0.0	20.4	12.9	3.6	0.9	2.7	1168.4	TEGm162
m210	0.4	0.3	1.7	0.1	0.0	2.0	0.4	33.4	1.3	19.4	36.5	4.5	1167.2	TEGm204
m346	0.1	3.2	26.4	4.5	0.0	9.5	0.6	36.5	13.7	5.5	0.0	0.0	1166.7	TEGm162
m335	0.4	3.5	27.0	4.1	0.0	9.8	0.6	33.8	14.0	6.9	0.0	0.0	1165.9	TEGm162
m443	0.3	3.5	26.9	5.1	0.0	12.5	1.0	32.3	10.5	3.7	0.9	3.3	1164.8	TEGm162
m621	0.1	4.7	41.1	6.0	0.7	11.9	0.1	17.8	13.3	3.3	1.0	0.0	1160.8	TEGm162
m434	0.2	3.3	26.5	5.4	0.5	13.0	1.2	30.2	11.8	3.6	1.0	3.2	1159.8	TEGm162
m182	0.3	0.3	2.1	0.3	0.0	2.7	0.4	33.7	2.2	17.7	34.5	6.0	1156.7	sequencing failed
m629	0.0	3.5	36.3	6.0	0.7	13.5	0.1	18.5	14.6	3.5	1.0	2.3	1154.1	TEGm162
m177	0.2	4.4	26.0	4.6	0.4	11.3	1.7	24.6	14.2	5.8	4.1	2.8	1152.9	TEGm162
m580	0.2	4.0	37.8	6.4	0.8	14.5	1.0	21.4	9.0	2.9	0.8	1.1	1151.4	TEGm162
m600	0.1	4.0	35.6	5.9	0.6	14.5	0.0	21.7	11.9	3.8	1.3	0.6	1149.4	TEGm162
m528	0.2	3.4	31.0	5.7	0.6	13.0	0.0	33.9	5.8	3.0	1.1	2.1	1146.8	TEGm162
m413	0.1	0.3	14.8	6.6	1.4	13.6	7.6	14.2	5.9	14.7	16.9	3.9	1141.7	TEGm413
m155	0.6	5.5	31.9	5.2	0.4	12.3	2.4	20.5	13.6	3.2	1.7	2.8	1135.0	TEGm162
m586	0.1	4.3	36.4	6.8	0.8	15.0	1.4	17.4	12.0	3.5	1.0	1.3	1128.9	TEGm162
m518	0.3	3.9	33.9	6.2	0.8	14.2	0.8	28.0	4.4	3.8	1.1	2.7	1127.6	TEGm162
m588	0.2	3.6	33.5	5.8	0.7	13.0	0.9	20.7	13.2	5.0	1.1	2.2	1126.3	TEGm162
m460	0.2	3.2	28.2	5.4	0.6	13.5	1.0	27.8	9.8	5.2	1.2	3.7	1126.2	TEGm162
m318	0.2	2.4	22.5	4.4	0.0	9.7	0.7	40.7	15.2	4.1	0.0	0.0	1120.9	TEGm162
m437	0.3	3.1	25.4	4.9	0.5	12.2	1.0	32.7	10.9	3.4	1.2	4.5	1119.6	TEGm162
m171	1.3	1.4	17.6	0.8	0.1	6.4	3.1	26.2	5.9	12.1	23.0	2.0	1118.5	TEGm171
m527	0.3	3.9	34.6	6.1	0.8	14.2	1.0	25.3	6.6	3.9	1.1	2.4	1114.6	TEGm162
m193	0.3	5.3	31.3	4.4	0.4	13.9	2.1	21.2	12.1	4.5	1.8	2.7	1114.2	TEGm162
m617	0.1	3.6	35.0	5.9	0.7	13.3	0.6	20.9	14.5	4.5	0.7	0.1	1110.0	TEGm162
m607	0.0	4.2	37.0	6.1	0.7	12.7	0.2	21.3	11.8	3.2	1.0	1.8	1109.7	TEGm162
m245	0.1	0.0	1.0	0.0	0.0	0.7	0.0	32.1	0.0	27.7	38.5	0.0	1105.6	TEGm245
m327	0.1	3.1	25.5	4.5	0.0	9.8	0.7	30.9	16.5	8.9	0.0	0.0	1105.3	TEGm162
m319	0.1	2.5	22.9	4.2	0.1	9.8	0.7	37.5	13.5	5.6	0.0	3.2	1102.9	TEGm162
m484	0.3	3.9	28.4	6.2	0.7	13.9	0.8	29.1	7.9	4.7	1.2	3.1	1100.0	TEGm162
m517	0.2	3.5	31.7	5.8	0.7	13.0	0.8	29.8	5.7	4.5	1.3	2.9	1085.3	TEGm162
m156	0.6	5.4	32.5	5.1	0.5	12.2	2.0	19.9	13.9	3.4	1.8	2.8	1084.1	TEGm162
m430	0.2	3.1	24.9	4.7	0.2	11.7	0.8	37.5	11.2	5.5	0.0	0.0	1079.9	TEGm162
m420	0.3	3.6	25.1	5.7	0.6	12.5	1.2	30.4	11.6	7.8	1.2	0.0	1078.7	TEGm162
m149	0.4	3.8	23.6	4.4	0.4	10.8	1.9	27.5	18.0	4.7	1.6	2.9	1078.4	TEGm162
m478	0.1	3.4	30.0	5.1	0.5	12.3	0.8	30.1	9.4	4.5	1.0	2.9	1071.5	TEGm162
m207	0.1	0.2	1.3	0.2	0.0	1.9	0.3	35.2	1.1	21.9	33.9	3.7	1070.3	sequencing failed
m502	0.4	4.7	31.4	5.8	0.7	13.8	1.2	21.5	7.2	8.3	2.0	3.0	1068.7	TEGm162
m180	0.1	3.1	23.8	5.3	0.4	13.0	1.6	27.6	14.8	5.0	2.2	3.1	1067.8	TEGm162
m218	0.4	3.2	24.3	5.7	0.4	12.5	0.7	32.3	9.5	8.5	2.5	0.0	1067.3	TEGm162
m326	0.1	2.0	19.4	3.2	0.0	7.6	0.5	43.5	14.8	8.9	0.0	0.0	1063.5	TEGm162
m150	0.5	4.4	27.3	4.8	0.4	11.3	1.7	24.4	15.2	4.4	1.8	3.8	1061.7	TEGm162
m426	0.2	3.3	27.7	5.5	0.3	13.2	1.1	27.3	12.4	4.2	1.5	3.3	1060.1	TEGm162
m442	0.3	3.3	26.3	5.2	0.0	12.9	1.0	29.4	10.2	6.1	1.3	4.0	1059.3	TEGm162
m152	0.5	5.2	30.4	4.9	0.5	11.9	2.4	23.9	15.0	3.6	1.8	0.0	1058.7	sequencing failed
m168	0.4	4.5	28.6	4.5	0.4	11.8	2.0	23.1	13.3	5.5	1.9	3.9	1051.5	TEGm162
m633	0.0	3.9	41.7	5.9	0.8	13.0	0.4	16.6	11.7	3.8	0.9	1.4	1050.6	sequencing failed
m439	0.2	3.3	27.6	5.1	0.5	12.3	1.1	28.0	11.4	5.0	1.3	4.1	1050.3	TEGm162
m336	0.2	2.4	21.5	3.8	0.0	9.1	0.4	41.5	13.3	7.8	0.0	0.0	1037.1	TEGm162
m334	0.1	2.0	19.0	3.6	0.0	8.6	0.4	42.7	14.1	9.5	0.0	0.0	1035.1	TEGm162
m498	0.2	3.3	27.4	6.2	0.8	13.9	0.9	31.4	6.8	4.5	1.3	3.2	1029.6	TEGm162
m151	0.6	5.0	27.9	4.9	0.4	11.6	2.3	22.8	14.7	4.8	1.9	3.2	1025.5	TEGm162
m520	0.0	0.2	2.4	3.1	1.5	8.8	10.3	24.0	15.0	11.4	20.6	2.8	1020.6	TEGm520
m611	0.1	3.3	30.7	5.6	0.6	12.7	1.4	24.1	13.5	4.2	1.0	2.7	1016.0	TEGm162
m438	0.3	3.1	24.9	5.2	0.0	13.2	1.3	32.2	10.6	4.4	1.4	3.6	1016.0	TEGm162
m202	0.4	0.4	2.8	0.3	0.0	4.0	0.8	30.1	2.9	17.9	36.4	4.0	1001.2	TEGm202
m339	0.1	2.6	23.3	4.0	0.0	9.3	0.6	39.0	13.0	8.1	0.0	0.0	994.8	sequencing failed
m565	0.3	4.2	39.0	1.9	5.3	12.3	0.9	19.5	10.3	3.7	1.1	1.6	989.6	TEGm162
m215	1.6	1.0	9.0	0.7	0.1	5.6	1.7	30.6	6.5	15.2	24.3	3.7	977.5	TEGm215
m571	0.0	4.0	34.3	6.5	0.7	14.7	0.0	20.1	12.4	4.3	1.1	2.0	973.6	TEGm162
m328	0.1	1.8	16.8	3.2	0.0	6.9	0.3	47.7	15.7	7.5	0.0	0.0	969.2	TEGm162
m428	0.3	3.2	24.5	5.2	0.2	11.9	0.9	32.0	10.3	6.1	1.4	4.2	961.2	TEGm162
m200	0.3	0.0	1.8	0.4	0.1	4.7	3.7	19.2	8.3	16.6	41.5	3.5	944.1	TEGm200
m211	0.0	0.3	1.4	0.1	0.0	1.5	0.2	35.6	1.2	18.9	40.9	0.0	943.4	TEGm204
m345	0.1	2.8	25.1	4.1	0.0	9.7	0.0	34.2	14.1	9.9	0.0	0.0	936.5	TEGm162
m212	0.1	0.3	1.2	0.1	0.0	1.3	0.1	39.1	1.4	18.0	38.5	0.0	929.4	TEGm198
m342	0.2	2.4	22.8	3.8	0.0	9.1	0.5	39.7	12.7	8.8	0.0	0.0	899.5	TEGm162
m205	0.0	0.1	0.3	0.0	0.0	0.7	0.0	35.9	1.1	23.9	34.1	3.9	890.8	TEGm205
m501	0.0	0.1	0.4	0.4	0.0	9.1	1.0	38.7	4.9	17.1	23.7	4.6	867.6	TEGm501
m449	0.4	3.4	25.4	5.3	0.0	12.7	1.0	29.8	10.0	7.6	1.3	3.1	854.0	TEGm162
m418	0.2	0.0	1.1	0.0	0.0	1.7	1.2	26.3	2.7	27.7	30.6	8.4	850.6	sequencing failed
m333	0.0	2.0	19.5	3.3	0.0	8.6	0.6	34.8	16.1	15.2	0.0	0.0	848.8	TEGm162
m630	0.0	4.0	38.2	6.1	0.6	12.5	0.6	16.1	13.1	5.8	0.9	2.1	847.7	TEGm162
m165	0.4	4.1	26.8	4.3	0.4	9.3	1.6	27.0	15.0	6.3	1.8	3.0	847.0	TEGm162
m181	0.4	0.5	3.7	0.7	0.0	4.8	0.5	35.5	4.6	15.1	29.2	5.0	828.7	TEGm181
m167	0.4	4.2	27.1	5.1	0.4	11.8	1.9	26.3	15.0	6.3	1.6	0.0	814.6	TEGm162
m195	0.5	5.1	32.3	5.2	0.5	10.2	1.6	19.2	13.1	7.4	2.7	2.3	808.8	TEGm162
m301	0.2	0.0	2.8	5.7	1.4	17.9	7.7	24.6	12.1	15.5	12.3	0.0	798.7	sequencing failed
m624	0.1	3.0	35.1	5.8	0.6	12.4	0.2	22.1	15.1	4.5	1.1	0.0	796.8	TEGm162
m551	0.4	7.5	15.9	8.5	0.9	19.7	1.6	26.5	6.9	7.3	1.9	2.8	777.9	TEGm162
m412	0.0	0.0	2.2	0.0	0.0	3.8	1.7	28.2	3.4	26.8	29.1	4.7	760.6	sequencing failed
m197	0.1	0.3	1.4	0.1	0.0	1.4	0.1	37.9	1.4	22.7	33.1	1.4	759.1	sequencing failed
m187	0.7	4.7	30.4	5.2	0.6	12.1	2.0	20.7	14.2	7.3	2.0	0.1	753.6	TEGm162
m596	0.0	3.3	31.8	5.1	0.5	10.8	0.0	24.5	15.5	5.1	1.4	2.1	750.9	TEGm162
m251	0.1	0.1	1.0	0.2	0.0	0.9	0.0	17.0	0.5	36.3	26.3	17.6	750.1	sequencing failed
m199	0.1	0.2	1.1	0.1	0.0	1.5	0.2	35.6	1.0	20.9	35.5	3.8	737.5	sequencing failed
m254	0.2	3.4	31.7	5.4	0.4	12.0	0.8	20.3	11.4	12.1	0.9	1.4	731.8	TEGm162
m198	0.1	0.3	1.3	0.1	0.0	1.4	0.1	36.4	0.7	23.0	36.6	0.0	704.5	TEGm198
m191	0.2	3.9	27.1	4.6	0.4	11.0	1.6	27.2	16.3	6.2	1.6	0.0	704.0	TEGm162
m424	0.3	2.6	23.6	4.7	0.2	11.9	0.8	33.1	12.9	9.8	0.0	0.0	678.1	TEGm162
m415	0.0	0.3	2.1	0.0	0.0	3.5	1.7	27.2	3.6	26.5	28.2	7.0	667.8	sequencing failed
m189	0.6	3.9	26.1	4.8	0.4	12.6	1.7	26.1	13.4	6.7	2.4	1.4	652.3	TEGm162
m176	0.6	0.9	8.4	1.2	0.0	7.8	1.2	39.5	10.0	14.0	16.3	0.0	630.3	sequencing failed
m248	0.4	0.0	0.5	0.3	0.0	15.7	0.7	42.0	10.9	21.6	8.0	0.0	621.6	sequencing failed
m262	0.3	1.5	11.8	2.5	0.1	5.7	0.2	46.8	15.5	15.6	0.0	0.0	609.2	TEGm162

m546	0.0	0.3	1.3	5.2	0.6	21.1	7.1	22.1	11.7	14.9	12.2	3.4	607.4	TEGm546
m492	0.2	0.2	2.5	1.5	0.0	8.6	0.0	21.9	2.8	25.7	24.0	12.5	603.7	TEGm492
m157	0.4	1.0	12.0	0.7	0.0	4.0	0.5	30.9	4.6	19.1	19.2	7.6	598.0	TEGm157
m618	0.1	4.1	36.4	5.4	0.6	13.3	0.0	18.3	14.4	6.3	0.9	0.0	595.1	TEGm162
m201	0.1	0.2	1.3	0.1	0.0	1.2	0.1	19.3	1.3	28.4	41.1	7.0	594.0	TEGm201
m400	0.0	0.3	8.0	11.0	0.3	13.1	5.2	27.4	9.5	19.9	5.3	0.0	580.4	sequencing failed
m219	0.1	0.5	2.4	0.3	0.0	4.4	0.2	54.7	3.8	16.1	17.5	0.0	528.2	TEGm219
m203	0.1	0.1	0.6	0.1	0.0	2.4	0.0	45.1	2.2	24.1	19.6	5.7	525.5	TEGm203

[a] The intensity of the green shading of each cell is proportional to the mol % of each fatty acid.

[b] The length of each red-bar in each cell is proportional to the fatty acid productivity

Supplemental Table 4a. Ranking of importance scores of individual residues that were evaluated by 10 independent random forest classificaiton models. Within each model, the p-values were corrected among all residue positions to control the false discovery rate <5%. The highest p-value among 10 models are reported for each position.

		Importance scores for 10 random forest classification models												
Residue position [a]	Residue Ranking	1	2	3	4	5	6	7	8	9	10	Mean	Number of significant p-values (p <0.001)	Corrected p-value
125	1	14.316	14.959	15.973	13.759	13.957	14.940	15.255	15.548	17.328	13.296	14.933	10	0.000
116	2	9.143	10.777	9.642	9.204	10.655	9.461	10.814	11.742	9.361	10.652	10.145	10	0.000
126	3	8.405	8.490	8.105	8.162	8.136	8.783	7.743	9.504	8.282	8.840	8.445	10	0.000
167	4	8.489	6.893	7.971	7.484	6.938	8.045	6.973	7.533	6.863	8.500	7.569	10	0.000
156	5	6.870	7.517	9.017	7.466	8.118	7.497	7.269	8.577	7.067	5.920	7.532	10	0.000
81	6	6.893	6.273	4.932	5.020	6.529	5.961	6.612	7.358	6.841	5.410	6.183	10	0.000
3	7	7.990	6.187	5.365	5.241	3.665	3.749	5.930	5.255	5.226	4.737	5.334	10	0.000
293	8	5.259	6.473	5.624	4.905	4.931	5.135	5.374	4.981	4.185	5.389	5.225	10	0.000
201	9	5.258	5.369	4.220	4.541	5.626	4.894	5.089	5.584	5.375	5.178	5.114	10	0.000
134	10	5.338	5.105	5.153	5.126	4.854	4.601	4.600	4.833	5.197	5.578	5.039	10	0.000
140	11	5.381	4.345	5.255	4.583	4.394	4.785	4.735	4.314	4.119	4.875	4.679	10	0.000
79	12	4.467	3.033	5.079	4.188	4.075	4.597	4.788	4.478	4.233	4.841	4.378	10	0.000
15	13	3.275	4.717	4.145	4.726	4.856	4.974	3.160	3.855	3.923	4.883	4.251	10	0.000
118	14	4.564	3.934	4.838	4.168	4.163	4.276	4.067	3.424	3.954	3.902	4.129	10	0.000
122	15	3.495	3.006	3.844	4.307	3.203	3.887	3.761	4.972	3.789	4.382	3.865	10	0.000
343	16	3.300	4.429	3.281	4.518	3.984	3.005	3.333	4.411	3.745	3.239	3.724	10	0.000
53	17	2.897	3.760	3.264	3.523	3.808	4.153	3.617	3.307	3.166	3.573	3.507	10	0.000
1	18	3.558	3.335	3.866	2.541	3.344	3.739	3.709	3.326	3.814	3.252	3.448	10	0.000
159	19	3.364	4.336	3.251	3.243	3.004	3.624	3.291	2.998	2.961	3.765	3.384	10	0.000
217	20	3.195	3.198	3.246	3.507	3.484	3.168	3.196	3.526	2.791	3.047	3.236	10	0.000
215	21	2.819	2.935	2.544	3.298	3.123	3.569	3.536	3.294	2.657	3.285	3.106	10	0.000
78	22	2.982	3.961	2.786	3.338	3.311	3.692	2.772	3.297	2.653	1.972	3.076	10	0.000
176	23	2.610	3.036	3.403	3.039	2.547	3.051	2.924	3.456	3.484	3.066	3.062	10	0.000
224	24	3.337	3.181	2.715	3.072	2.307	2.942	2.632	3.156	3.225	3.326	2.989	10	0.000
101	25	2.563	2.875	2.636	2.868	3.133	3.179	2.757	2.949	2.939	3.306	2.920	10	0.000
128	26	2.205	3.503	2.072	3.572	2.743	3.527	3.336	1.510	1.858	3.840	2.817	10	0.000
344	27	3.047	2.895	2.306	2.630	2.682	3.333	2.848	2.483	2.758	2.848	2.783	10	0.000
342	28	2.270	2.855	3.044	2.730	3.088	2.758	2.650	2.468	3.078	2.283	2.722	10	0.000
138	29	2.999	2.798	2.348	2.023	2.535	3.175	2.720	2.320	2.650	2.823	2.639	10	0.000
146	30	2.731	2.235	2.359	2.929	2.699	2.998	2.851	2.799	1.977	2.718	2.630	10	0.000
210	31	3.197	3.184	3.084	1.505	2.692	2.331	2.353	1.500	2.721	3.056	2.562	10	0.000
179	32	2.581	2.638	2.365	2.357	2.478	2.108	2.751	2.702	2.326	2.557	2.486	10	0.000
2	33	2.707	2.553	2.409	2.676	2.999	2.528	1.539	2.220	2.901	1.938	2.447	10	0.000
155	34	2.137	2.720	2.799	2.436	2.559	2.246	2.122	2.274	2.580	2.287	2.416	10	0.000
147	35	2.389	2.406	2.698	2.386	2.175	2.289	2.060	2.344	2.444	2.146	2.334	10	0.000
170	36	2.748	2.113	2.066	2.361	2.369	2.749	2.347	2.083	2.108	2.322	2.327	10	0.000
123	37	1.535	2.551	1.324	2.175	2.363	2.191	3.092	3.459	2.422	2.144	2.326	10	0.000
151	38	2.467	2.488	2.492	1.801	2.435	2.371	2.340	2.020	2.665	2.140	2.322	10	0.000
139	39	2.205	2.590	1.504	2.821	2.339	2.617	2.467	2.173	2.006	2.103	2.283	10	0.000
112	40	2.334	2.136	2.813	2.161	1.907	1.835	2.173	2.733	2.243	2.206	2.254	10	0.000
184	41	1.791	2.045	2.304	2.271	2.284	2.746	1.923	2.366	2.164	2.551	2.244	10	0.000
257	42	1.899	1.970	1.998	3.061	2.064	2.181	2.319	1.969	2.753	1.816	2.203	10	0.000
108	43	2.308	2.893	2.405	2.212	1.479	1.723	2.826	1.313	2.421	2.055	2.164	10	0.000
180	44	2.052	2.201	2.243	1.818	2.164	2.267	2.447	1.612	2.119	2.540	2.146	10	0.000
4	45	1.848	1.925	1.999	2.159	2.347	1.794	2.175	2.457	2.250	2.281	2.123	10	0.000
131	46	1.687	1.891	2.094	2.409	1.963	2.290	2.201	2.311	2.290	1.941	2.108	10	0.000
189	47	2.014	1.965	2.351	2.371	1.996	1.989	2.049	2.159	2.013	1.814	2.072	10	0.000
175	48	1.712	2.404	2.338	1.804	1.781	2.158	1.795	1.699	1.799	2.488	1.998	10	0.000
10	49	1.623	1.606	2.438	1.669	2.046	2.660	2.095	2.214	1.768	1.786	1.991	10	0.000
186	50	1.992	1.976	1.837	2.029	1.964	1.977	1.700	2.265	2.281	1.796	1.982	10	0.000
75	51	2.160	1.905	1.878	1.977	1.406	2.360	1.918	2.131	2.218	1.840	1.979	10	0.000
25	52	1.796	1.737	1.824	3.399	2.085	1.855	2.411	1.857	1.295	1.469	1.973	10	0.000
228	53	2.706	2.891	1.299	2.664	2.155	2.018	1.330	1.634	0.933	1.972	1.960	10	0.000
158	54	1.712	2.089	2.132	1.524	1.408	1.791	2.439	2.355	1.829	1.825	1.911	10	0.000
93	55	1.882	1.877	1.622	2.090	1.941	1.827	1.791	1.836	1.748	2.247	1.886	10	0.000
82	56	2.367	1.814	1.614	1.837	2.187	1.551	1.853	1.830	2.304	1.464	1.882	10	0.000
68	57	2.135	1.912	2.001	1.892	1.647	1.861	1.733	1.461	2.197	1.834	1.867	10	0.000
95	58	2.011	1.708	0.898	1.801	2.094	2.534	2.703	1.457	1.667	1.657	1.853	10	0.000
198	59	1.747	1.720	1.864	1.973	1.820	2.215	1.746	1.959	1.386	2.027	1.846	10	0.000
130	60	1.434	1.901	1.914	1.681	1.808	1.698	1.731	1.921	2.179	1.604	1.787	10	0.000
165	61	1.554	1.749	1.865	2.081	1.969	1.723	1.575	1.746	1.876	1.574	1.771	10	0.000
100	62	1.837	1.727	1.920	2.196	1.371	1.738	1.699	1.533	2.265	1.384	1.767	10	0.000
47	63	1.985	1.380	1.786	1.713	1.853	1.926	1.764	1.459	1.604	1.938	1.741	10	0.000
103	64	2.285	1.171	2.031	0.943	2.083	1.318	1.067	1.241	2.472	2.195	1.681	10	0.000
238	65	1.830	1.616	1.362	1.649	1.890	1.570	1.627	1.770	1.863	1.544	1.672	10	0.000
117	66	1.795	1.825	1.494	1.491	1.799	1.591	1.530	1.476	1.695	1.963	1.666	10	0.000
166	67	1.346	1.353	1.044	2.246	2.479	1.028	1.864	1.773	1.646	1.828	1.661	10	0.000
348	68	1.838	1.427	1.607	1.669	1.566	1.916	1.635	1.372	1.575	1.778	1.638	10	0.000
8	69	1.630	1.288	1.938	1.971	1.675	1.319	1.416	1.634	1.590	1.911	1.637	10	0.000
143	70	1.392	1.718	1.918	1.393	1.562	1.680	1.773	1.396	1.670	1.849	1.635	10	0.000
303	71	1.891	1.815	1.768	1.822	1.361	1.547	1.443	1.639	1.562	1.391	1.624	10	0.000
350	72	1.609	1.365	1.265	1.788	1.780	1.817	1.377	1.574	1.620	1.847	1.604	10	0.000
46	73	1.102	1.135	2.034	2.085	1.723	1.208	1.200	1.325	1.545	2.147	1.550	10	0.000
191	74	1.589	1.438	1.470	1.768	1.397	1.925	1.311	1.457	1.646	1.417	1.542	10	0.000
83	75	0.971	1.750	1.102	1.366	1.654	1.183	1.938	1.532	2.211	1.686	1.539	10	0.000
200	76	1.452	1.522	1.568	1.881	1.520	1.095	2.089	1.352	1.370	1.529	1.538	10	0.000
20	77	1.268	1.520	2.018	1.344	1.601	1.407	1.639	1.564	1.635	1.297	1.529	10	0.000
205	78	1.554	1.027	1.543	1.743	1.846	1.624	1.302	1.442	1.367	1.378	1.482	10	0.000
57	79	1.381	1.753	1.820	2.054	1.313	1.066	0.967	0.530	1.539	2.129	1.455	10	0.000
207	80	1.704	1.363	0.909	1.729	0.874	1.709	1.038	0.813	2.068	2.261	1.447	10	0.000
21	81	1.302	1.270	1.083	1.982	1.589	1.381	1.812	1.055	1.271	1.552	1.430	10	0.000

349	82	1.530	1.550	1.553	1.531	1.594	1.309	1.309	1.297	0.948	1.525	1.415	10	0.000
66	83	1.576	1.907	0.833	1.657	1.141	1.684	1.217	1.341	1.995	0.766	1.412	10	0.000
80	84	1.368	1.465	1.649	1.391	1.607	1.289	1.207	1.394	1.334	1.410	1.411	10	0.000
110	85	1.261	1.187	1.424	1.323	1.330	1.381	1.487	1.727	1.528	1.444	1.409	10	0.000
13	86	1.272	1.186	1.122	1.474	1.921	1.615	1.172	1.410	1.654	1.174	1.400	10	0.000
164	87	1.207	0.840	1.363	1.594	1.427	2.089	1.028	1.116	1.190	1.694	1.355	10	0.000
249	88	1.688	1.229	1.360	1.321	1.281	0.946	1.810	1.397	1.616	0.803	1.345	10	0.000
106	89	1.528	0.526	1.658	1.371	1.264	1.111	1.604	1.328	1.159	1.804	1.335	10	0.000
168	90	0.951	1.288	1.669	1.470	1.644	1.317	1.108	1.260	1.128	1.332	1.317	10	0.000
120	91	1.582	0.804	1.944	1.186	1.592	1.457	0.962	1.445	1.242	0.936	1.315	10	0.000
19	92	1.629	0.578	1.001	1.408	1.793	1.500	1.190	1.350	0.905	1.752	1.311	10	0.000
255	93	0.709	1.423	1.667	0.787	1.997	1.092	1.582	1.967	1.026	0.852	1.310	10	0.000
182	94	1.210	1.026	1.171	1.461	1.254	1.484	1.134	1.130	1.338	1.461	1.267	10	0.000
245	95	1.196	1.320	0.969	1.157	1.087	1.273	1.423	1.403	1.592	1.106	1.253	10	0.000
346	96	1.531	1.128	1.372	1.086	1.059	1.092	1.347	1.224	1.279	1.408	1.253	10	0.000
88	97	1.089	1.189	1.300	1.250	1.465	1.301	1.045	1.286	1.210	1.328	1.246	10	0.000
347	98	0.988	1.331	1.415	0.905	1.114	1.600	1.575	1.042	1.129	1.141	1.224	10	0.000
172	99	1.375	1.199	1.422	1.368	1.384	1.139	1.115	1.445	0.966	0.748	1.216	10	0.000
6	100	1.211	1.361	1.342	1.111	1.217	1.285	1.109	1.170	1.136	1.088	1.203	10	0.000
60	101	0.991	1.042	1.443	1.538	1.237	1.211	1.232	1.235	1.020	1.074	1.202	10	0.000
244	102	1.241	0.950	1.309	1.220	1.073	1.182	1.087	1.442	1.093	1.366	1.196	10	0.000
206	103	0.902	0.931	1.111	1.285	1.360	1.245	1.332	1.095	1.403	0.974	1.164	10	0.000
67	104	1.423	1.319	1.004	0.767	1.464	1.459	1.045	1.317	0.546	1.067	1.141	10	0.000
124	105	1.041	1.061	1.018	1.271	1.284	0.944	0.805	1.344	1.106	1.536	1.141	10	0.000
22	106	0.887	1.271	0.996	1.412	0.970	1.390	0.913	1.108	1.064	1.295	1.131	10	0.000
261	107	0.978	1.120	0.975	1.076	1.211	1.263	1.179	1.018	0.901	1.551	1.127	10	0.000
265	108	1.684	0.705	1.321	1.144	0.769	1.195	1.152	1.206	1.037	1.031	1.124	10	0.000
227	109	0.969	1.115	1.134	1.250	1.041	1.357	1.114	1.349	0.761	1.128	1.122	10	0.000
113	110	0.996	0.997	1.513	1.009	1.077	1.055	1.170	1.032	1.509	0.803	1.116	10	0.000
341	111	1.248	1.254	1.255	0.971	0.847	1.370	0.822	1.475	0.942	0.932	1.112	10	0.000
77	112	1.161	1.101	1.099	1.060	1.075	1.289	1.097	1.243	0.796	1.180	1.110	10	0.000
157	113	0.891	1.671	0.826	1.430	0.836	1.124	0.920	1.682	0.745	0.924	1.105	10	0.000
345	114	1.108	1.008	1.046	1.415	0.760	0.754	1.305	1.077	1.012	1.409	1.089	10	0.000
295	115	0.954	1.229	1.278	0.448	1.390	1.186	1.268	1.126	1.026	0.948	1.085	10	0.000
169	116	0.637	1.357	0.917	0.489	2.247	0.594	0.597	1.157	1.333	1.475	1.080	10	0.000
246	117	1.606	0.755	0.716	1.448	0.618	0.751	1.234	1.104	1.605	0.940	1.078	10	0.000
305	118	1.044	1.495	0.766	1.174	0.987	0.910	0.977	1.234	1.009	0.969	1.057	10	0.000
236	119	1.175	0.984	0.953	1.064	0.891	1.509	1.194	1.066	0.865	0.814	1.052	10	0.000
17	120	0.708	1.453	0.631	0.999	1.420	0.880	0.924	1.573	1.077	0.847	1.051	10	0.000
291	121	1.022	0.943	0.999	0.981	1.122	1.103	1.014	0.970	1.066	1.244	1.046	10	0.000
230	122	0.686	0.970	0.848	1.205	1.323	1.091	1.056	0.881	1.416	0.959	1.043	10	0.000
9	123	0.936	0.916	1.191	1.016	1.189	1.032	0.933	0.995	1.028	1.044	1.028	10	0.000
161	124	0.983	0.926	1.197	1.045	0.796	1.227	1.031	1.108	0.839	1.084	1.024	10	0.000
16	125	0.968	0.904	0.741	0.744	0.987	1.215	1.107	0.977	1.188	1.271	1.010	10	0.000
340	126	1.156	0.975	1.299	0.969	0.767	0.966	0.949	1.231	0.793	0.757	0.986	10	0.000
97	127	0.967	0.892	1.022	0.694	1.021	1.313	0.669	1.139	1.346	0.800	0.986	10	0.000
54	128	0.570	1.530	0.225	0.427	1.940	1.255	0.601	0.897	1.065	1.171	0.968	9	0.134
267	129	0.942	1.018	1.152	1.132	0.818	0.856	0.870	0.908	0.995	0.716	0.941	10	0.000
268	130	0.972	0.812	0.792	0.972	0.903	0.965	1.213	0.904	0.778	1.062	0.937	10	0.000
213	131	1.306	0.975	0.886	0.804	0.816	1.019	0.888	0.776	0.984	0.895	0.935	10	0.000
183	132	0.678	1.114	0.819	0.773	1.135	0.845	1.007	1.021	1.158	0.799	0.935	10	0.000
335	133	1.086	0.848	0.907	0.897	0.717	1.021	0.884	1.022	1.000	0.881	0.926	10	0.000
292	134	0.956	0.645	1.275	0.778	0.867	1.072	1.095	0.722	1.180	0.611	0.920	10	0.000
222	135	0.824	1.095	1.122	0.976	0.769	0.831	0.669	0.905	1.001	0.907	0.910	10	0.000
5	136	0.757	0.885	1.038	0.504	0.776	1.194	0.927	1.261	0.830	0.924	0.910	10	0.000
111	137	1.017	0.710	0.768	0.637	0.903	1.287	1.411	0.912	0.664	0.758	0.907	10	0.000
327	138	1.137	0.729	0.329	1.104	0.256	0.559	1.145	1.303	1.586	0.609	0.876	8	0.039
7	139	0.681	0.917	0.875	0.752	0.806	1.101	0.929	0.602	1.131	0.738	0.853	10	0.000
11	140	0.959	0.943	0.873	0.474	0.622	0.750	0.949	1.006	0.940	0.952	0.847	10	0.000
260	141	0.903	0.951	0.950	1.118	0.734	0.402	0.920	0.922	1.233	0.331	0.846	10	0.000
36	142	0.569	0.939	1.244	0.589	0.524	0.846	0.919	0.798	1.225	0.642	0.829	10	0.000
264	143	0.877	0.895	0.876	0.508	1.056	0.886	0.752	0.915	0.704	0.781	0.825	10	0.000
296	144	0.575	0.871	0.747	0.927	0.868	1.130	0.852	0.673	0.783	0.783	0.821	10	0.000
247	145	0.812	1.025	0.705	0.679	0.891	0.910	0.948	0.861	0.903	0.471	0.820	10	0.000
49	146	0.708	1.159	1.179	0.681	0.532	0.981	1.006	0.630	0.703	0.513	0.809	10	0.000
272	147	0.726	0.898	0.628	0.577	0.620	0.951	0.925	1.134	0.914	0.710	0.808	10	0.000
258	148	0.515	0.849	0.502	1.466	0.722	0.843	0.708	0.927	0.751	0.591	0.787	10	0.000
98	149	0.691	0.589	0.594	1.298	0.832	0.740	0.635	0.775	0.785	0.880	0.782	10	0.000
290	150	0.582	0.887	0.836	0.923	0.433	0.604	0.689	1.129	0.779	0.862	0.772	10	0.000
262	151	0.906	0.367	0.716	0.907	0.656	0.742	0.892	0.651	0.862	0.989	0.769	10	0.000
85	152	0.813	0.899	0.528	0.763	0.713	0.730	0.698	0.910	0.621	0.757	0.743	10	0.000
50	153	0.917	0.668	0.544	0.789	0.672	0.492	0.743	0.921	0.981	0.695	0.742	10	0.000
339	154	0.646	0.749	0.691	0.880	0.582	0.907	0.647	0.367	0.875	0.999	0.734	10	0.000
332	155	0.711	0.885	0.612	0.563	0.760	0.808	0.978	0.510	0.872	0.613	0.731	10	0.000
253	156	0.610	0.828	0.854	0.846	0.614	0.953	0.903	0.435	0.712	0.485	0.724	10	0.000
64	157	0.711	0.829	0.531	0.771	0.634	0.790	0.888	0.768	0.541	0.689	0.715	10	0.000
242	158	0.771	0.527	0.634	0.791	0.561	0.750	0.960	0.708	0.718	0.702	0.712	10	0.000
308	159	0.413	0.843	0.653	0.855	0.749	0.544	0.744	0.993	0.893	0.419	0.711	9	0.011
336	160	0.582	0.729	0.744	0.584	0.774	0.477	0.757	0.954	0.580	0.658	0.684	10	0.000
338	161	0.717	0.405	0.549	0.555	0.550	0.799	0.763	0.942	0.757	0.794	0.683	10	0.000
326	162	0.537	0.342	0.797	0.765	0.925	0.477	0.738	0.554	0.922	0.754	0.681	9	0.011
212	163	0.782	0.200	1.085	0.678	0.517	0.655	0.350	0.500	0.495	1.521	0.678	9	0.203
248	164	0.648	0.825	0.652	0.903	0.642	0.591	0.471	0.764	0.775	0.512	0.678	10	0.000
234	165	0.492	0.640	0.529	0.959	0.739	0.830	0.429	0.864	0.590	0.665	0.674	10	0.000
239	166	0.463	0.441	0.520	0.573	0.864	0.858	0.615	0.568	0.960	0.841	0.670	10	0.000
86	167	0.587	0.625	0.609	0.559	0.793	0.513	0.517	0.667	0.721	0.942	0.653	10	0.000
14	168	0.684	0.651	0.585	0.561	0.585	0.765	0.599	0.781	0.574	0.705	0.649	10	0.000
271	169	0.387	0.473	0.592	0.881	0.874	0.679	0.461	0.572	0.814	0.738	0.647	9	0.022
94	170	0.471	0.849	0.854	1.093	0.311	0.845	0.512	0.459	0.459	0.608	0.646	9	0.010

331	171	0.547	0.590	0.619	0.676	0.522	0.646	0.645	0.791	0.778	0.611	0.643	10	0.000
133	172	0.793	0.522	0.509	0.552	0.577	0.760	0.804	0.569	0.572	0.701	0.636	10	0.000
288	173	0.482	0.504	0.786	0.667	0.717	0.960	0.658	0.669	0.474	0.416	0.633	10	0.000
337	174	0.624	0.903	0.639	0.559	0.670	0.614	0.536	0.574	0.689	0.462	0.627	10	0.000
266	175	0.568	0.865	0.350	0.647	0.533	0.432	0.720	0.706	0.712	0.485	0.602	10	0.000
74	176	0.643	1.033	0.494	0.656	0.339	0.745	0.542	0.495	0.565	0.485	0.600	10	0.000
306	177	0.713	0.588	0.568	0.638	0.527	0.612	0.807	0.579	0.679	0.247	0.596	9	0.089
273	178	0.525	0.533	0.591	0.483	0.502	0.659	0.674	0.542	0.612	0.757	0.588	10	0.000
127	179	0.742	0.324	0.609	0.507	0.418	0.502	0.764	0.606	0.620	0.777	0.587	9	0.011
55	180	0.746	0.352	0.772	0.455	0.717	0.767	0.534	0.364	0.690	0.449	0.585	9	0.011
18	181	0.578	0.671	0.541	0.472	0.504	0.578	0.528	0.522	0.656	0.577	0.563	10	0.000
233	182	0.661	0.368	0.861	0.550	0.309	0.675	0.733	0.826	0.376	0.221	0.558	8	0.116
84	183	0.779	1.256	0.714	0.786	0.366	0.575	0.198	0.048	0.213	0.557	0.549	7	0.530
12	184	0.421	0.176	0.690	0.635	0.581	0.874	0.535	0.467	0.606	0.450	0.544	8	0.267
45	185	1.072	0.430	0.540	0.856	0.107	0.351	0.248	0.124	0.323	1.285	0.534	7	0.395
105	186	0.515	0.733	0.525	0.626	0.345	0.428	0.542	0.406	0.464	0.739	0.532	10	0.000
24	187	0.545	0.641	0.231	0.470	0.563	0.419	0.588	0.678	0.526	0.632	0.529	9	0.125
270	188	0.668	0.509	0.456	0.833	0.336	0.498	0.556	0.404	0.560	0.413	0.523	10	0.000
211	189	0.789	0.376	0.415	0.364	0.459	0.699	0.512	0.560	0.516	0.521	0.521	9	0.010
63	190	0.015	1.048	1.018	0.252	0.662	0.414	0.356	0.560	0.533	0.335	0.519	8	0.595
114	191	0.358	0.378	0.444	0.536	0.304	0.495	0.800	0.218	0.581	0.994	0.511	7	0.156
119	192	0.783	0.596	0.232	0.511	0.346	0.488	0.523	0.661	0.674	0.211	0.503	8	0.134
289	193	0.677	0.258	0.431	0.479	0.737	0.493	0.498	0.271	0.660	0.511	0.501	8	0.128
51	194	0.166	0.186	0.754	0.822	0.151	0.300	0.550	0.753	0.495	0.714	0.489	6	0.337
304	195	0.518	0.345	0.111	0.501	0.614	0.511	0.486	0.382	0.612	0.690	0.477	8	0.428
287	196	0.546	0.368	0.421	0.594	0.432	0.693	0.424	0.468	0.466	0.356	0.477	10	0.000
28	197	0.703	0.254	0.373	0.169	0.260	0.643	0.843	0.566	0.364	0.573	0.475	7	0.327
286	198	0.607	0.379	0.401	0.625	0.181	0.697	0.404	0.635	0.392	0.427	0.475	9	0.213
333	199	0.312	0.275	0.423	0.387	0.144	0.776	0.449	0.715	0.545	0.709	0.473	7	0.300
42	200	0.000	0.980	0.800	0.396	0.231	0.462	0.542	0.519	0.492	0.304	0.473	7	0.656
329	201	0.435	0.332	0.427	0.481	0.420	0.435	0.714	0.249	0.482	0.724	0.470	7	0.080
297	202	0.320	0.569	0.200	0.595	0.726	0.755	0.419	0.371	0.234	0.431	0.462	7	0.224
69	203	0.404	0.312	0.671	0.543	0.639	0.558	0.577	0.422	0.245	0.237	0.461	6	0.097
299	204	0.656	0.380	0.431	0.566	0.346	0.401	0.538	0.545	0.348	0.298	0.451	9	0.010
96	205	0.430	0.403	0.813	0.449	0.317	0.167	0.591	0.230	0.430	0.597	0.443	6	0.307
137	206	0.440	0.290	0.476	0.453	0.632	0.260	0.363	0.319	0.345	0.761	0.434	6	0.051
256	207	0.309	0.599	0.702	0.310	0.569	0.288	0.367	0.477	0.415	0.272	0.431	6	0.049
177	208	0.699	0.327	0.580	0.486	0.442	0.424	0.236	0.477	0.404	0.217	0.429	7	0.126
199	209	0.482	0.460	0.373	0.311	0.605	0.661	0.283	0.510	0.151	0.347	0.418	7	0.325
142	210	0.411	0.461	0.268	0.345	0.742	0.602	0.231	0.471	0.234	0.364	0.413	5	0.108
39	211	0.134	0.300	0.502	0.631	0.488	0.276	0.544	0.469	0.478	0.295	0.412	6	0.397
208	212	0.363	0.378	0.404	0.333	-0.021	0.407	0.390	0.707	0.818	0.313	0.409	7	0.714
150	213	0.363	0.229	0.494	0.458	0.318	0.299	0.411	0.422	0.681	0.306	0.398	5	0.136
190	214	0.434	0.424	0.457	0.482	0.248	0.389	0.339	0.340	0.412	0.278	0.380	7	0.059
178	215	0.393	0.378	0.445	0.373	0.244	0.666	0.313	0.407	0.159	0.405	0.378	6	0.306
321	216	0.128	0.330	0.642	0.435	0.537	0.154	0.423	0.395	0.496	0.236	0.377	6	0.405
237	217	0.614	0.233	0.332	0.421	0.313	0.274	0.350	0.453	0.411	0.331	0.373	6	0.136
243	218	0.085	0.632	0.127	0.360	0.735	0.067	0.377	0.465	0.273	0.554	0.367	6	0.483
294	219	0.398	0.313	0.365	0.329	0.412	0.177	0.514	0.318	0.352	0.487	0.367	5	0.243
129	220	0.115	0.318	0.289	0.236	0.258	0.558	0.572	0.552	0.226	0.477	0.360	4	0.438
30	221	0.284	0.594	0.316	0.522	0.537	0.271	0.163	0.335	0.262	0.305	0.359	4	0.251
99	222	0.390	0.124	0.379	0.551	0.394	0.382	0.457	0.275	0.306	0.305	0.356	6	0.432
27	223	0.447	0.307	0.261	0.426	0.200	0.237	0.345	0.511	0.386	0.371	0.349	6	0.141
32	224	0.389	0.257	0.304	0.407	-0.045	0.372	0.398	0.690	0.208	0.506	0.349	5	0.750
300	225	0.380	0.427	0.305	0.759	0.370	0.210	-0.072	0.244	0.561	0.240	0.342	4	0.781
56	226	0.454	0.430	0.169	0.360	0.278	0.267	0.244	0.424	0.736	-0.008	0.335	4	0.656
219	227	0.316	0.004	0.194	0.223	0.336	0.981	0.597	0.227	0.035	0.441	0.335	4	0.622
174	228	0.429	0.297	0.680	0.324	0.523	0.116	0.144	0.435	0.164	0.185	0.330	3	0.416
328	229	0.137	0.483	0.349	0.336	0.203	0.637	0.157	0.087	0.369	0.538	0.330	5	0.459
259	230	0.420	0.297	0.163	0.435	0.400	0.336	0.342	0.333	0.278	0.204	0.321	6	0.292
71	231	0.476	0.644	0.341	0.427	0.290	0.189	0.307	-0.041	0.409	0.050	0.309	4	0.706
90	232	0.648	0.052	0.610	-0.123	0.384	0.191	0.251	0.081	0.503	0.461	0.306	5	0.795
185	233	0.401	0.163	0.278	0.257	0.398	0.110	0.229	0.409	0.204	0.537	0.299	3	0.416
70	234	0.496	0.376	0.242	0.262	0.500	0.275	0.056	0.246	0.184	0.349	0.299	4	0.528
26	235	0.298	0.152	0.243	0.342	0.081	0.456	0.205	0.306	0.318	0.485	0.288	3	0.452
102	236	0.289	0.248	0.202	0.197	0.161	0.296	0.511	0.133	0.551	0.274	0.286	2	0.364
240	237	0.229	0.538	0.304	0.184	0.376	0.174	0.174	0.151	0.411	0.323	0.286	4	0.312
241	238	0.265	0.132	0.345	0.312	0.228	0.449	0.332	0.254	0.303	0.151	0.277	4	0.409
135	239	0.237	0.241	0.259	0.268	0.216	0.246	0.211	0.172	0.443	0.399	0.269	2	0.257
152	240	0.140	0.351	0.279	0.495	0.274	0.317	0.118	0.378	0.086	0.232	0.267	3	0.491
173	241	0.231	0.389	0.190	0.389	0.319	0.155	0.259	0.107	0.252	0.166	0.246	3	0.437
330	242	0.118	0.127	0.228	0.115	0.303	0.135	0.350	0.371	0.319	0.282	0.235	3	0.471
37	243	0.153	0.269	0.181	0.234	0.091	0.248	0.276	0.223	0.205	0.455	0.233	1	0.428
87	244	0.378	0.421	0.197	0.141	0.046	0.123	0.379	0.217	0.037	0.359	0.230	3	0.533
301	245	0.131	0.292	0.242	0.303	0.073	0.332	0.162	0.174	0.106	0.372	0.219	2	0.456
285	246	0.074	0.093	0.206	0.197	0.020	0.463	0.341	0.351	0.167	0.212	0.212	3	0.563
203	247	-0.069	0.168	0.264	0.278	0.418	0.210	0.197	0.112	0.205	0.254	0.204	1	0.748
269	248	0.136	0.230	0.201	0.103	-0.019	0.252	0.539	0.135	-0.018	0.272	0.183	1	0.698
109	249	0.135	0.169	0.081	0.307	0.193	0.241	0.021	0.058	0.471	0.065	0.174	1	0.569
192	250	0.158	0.151	0.109	0.144	0.075	0.174	0.257	0.177	0.246	0.180	0.167	0	0.456
334	251	0.106	0.075	0.096	0.288	0.179	0.053	0.118	0.170	0.461	0.116	0.166	1	0.523
298	252	0.285	0.116	-0.012	0.089	0.194	-0.029	0.137	0.158	0.269	0.295	0.150	1	0.685
29	253	0.138	0.362	0.192	-0.074	0.009	0.018	0.273	0.007	0.428	0.075	0.143	1	0.702
202	254	0.225	0.120	0.077	0.152	0.084	0.214	0.014	0.136	0.157	0.121	0.130	0	0.579
65	255	-0.150	0.207	0.079	0.224	0.191	0.158	0.230	0.055	0.250	-0.039	0.120	0	0.831
284	256	-0.059	-0.175	0.136	0.354	0.023	0.310	0.198	0.116	0.064	0.099	0.106	0	0.885
59	257	0.098	-0.051	0.085	0.010	0.234	0.017	0.025	0.058	0.217	0.264	0.096	0	0.699
171	258	0.033	0.199	0.151	0.116	-0.081	0.060	0.180	-0.048	0.104	0.152	0.086	0	0.795
322	259	0.036	0.043	0.093	0.162	-0.028	0.090	-0.020	0.033	0.337	0.083	0.083	1	0.724

76	260	0.027	-0.024	0.087	0.067	0.149	-0.082	-0.060	0.183	0.085	0.317	0.075	1	0.769
41	261	0.138	-0.108	0.006	0.249	0.159	0.185	-0.091	0.114	0.053	0.022	0.073	0	0.808
35	262	0.055	0.243	0.077	0.088	-0.039	-0.077	-0.004	0.101	-0.008	0.010	0.045	0	0.759
204	263	0.073	0.021	-0.083	-0.001	0.108	0.081	-0.011	-0.082	0.187	0.128	0.042	0	0.775
323	264	0.047	0.277	0.045	-0.020	-0.024	0.070	-0.007	-0.061	0.113	-0.062	0.038	0	0.744
250	265	0.113	0.075	0.138	-0.119	0.045	0.089	0.064	-0.149	0.073	0.002	0.033	0	0.866
136	266	-0.054	0.056	0.013	0.081	-0.004	0.095	0.004	0.024	0.073	-0.019	0.027	0	0.737
277	267	0.029	0.083	0.027	0.013	-0.055	0.059	0.005	0.037	0.100	-0.093	0.021	0	0.790
162	268	0.374	-0.140	-0.064	-0.084	0.073	0.239	0.010	0.028	-0.044	-0.228	0.016	0	0.956
282	269	-0.016	0.089	-0.018	-0.049	0.011	0.110	-0.012	-0.092	0.085	0.041	0.015	0	0.785
145	270	-0.029	0.113	-0.078	0.016	-0.082	-0.083	0.070	0.086	0.030	0.083	0.013	0	0.800
31	271	-0.033	0.020	-0.141	0.009	0.027	-0.036	0.240	-0.012	-0.027	0.044	0.009	0	0.837
34	272	-0.165	0.042	0.103	0.064	0.035	0.143	-0.037	-0.281	0.174	0.006	0.008	0	0.989
283	273	-0.020	-0.065	0.092	-0.030	-0.006	0.087	0.024	0.035	0.043	-0.085	0.007	0	0.759
225	274	-0.025	-0.057	-0.058	-0.092	-0.204	0.417	0.014	0.053	0.105	-0.102	0.005	1	0.952
48	275	0.369	-0.093	-0.078	0.023	-0.071	-0.055	-0.141	-0.087	0.055	0.106	0.003	0	0.880
280	276	0.009	0.130	-0.015	0.092	-0.012	-0.018	-0.056	-0.046	0.042	-0.103	0.002	0	0.806
278	277	-0.025	0.059	-0.067	0.099	-0.045	0.031	0.024	-0.017	-0.011	-0.033	0.002	0	0.745
91	278	0.000	0.000	0.000	0.000	0.000	0.000	0.000	0.000	0.000	0.000	0.000	0	0.665
216	279	0.000	0.000	0.000	0.000	0.000	0.000	0.000	0.000	0.000	0.000	0.000	0	0.665
218	280	0.000	0.000	0.000	0.000	0.000	0.000	0.000	0.000	0.000	0.000	0.000	0	0.665
220	281	0.000	0.000	0.000	0.000	0.000	0.000	0.000	0.000	0.000	0.000	0.000	0	0.665
226	282	0.000	0.000	0.000	0.000	0.000	0.000	0.000	0.000	0.000	0.000	0.000	0	0.665
187	283	0.030	-0.228	0.291	-0.165	-0.013	0.235	-0.017	-0.027	-0.174	0.012	-0.006	0	0.945
281	284	0.089	-0.071	-0.044	0.036	0.032	0.012	-0.069	-0.018	0.019	-0.082	-0.010	0	0.776
276	285	-0.008	0.025	-0.035	-0.117	0.096	0.084	-0.047	-0.026	0.067	-0.143	-0.010	0	0.855
148	286	-0.106	-0.304	0.090	0.044	0.062	-0.082	-0.039	0.051	0.183	-0.032	-0.013	0	0.985
121	287	0.104	-0.186	0.028	-0.064	-0.184	0.097	-0.009	-0.073	0.112	0.021	-0.015	0	0.921
33	288	0.063	-0.023	-0.040	-0.089	0.056	-0.085	-0.120	0.058	0.035	-0.042	-0.019	0	0.847
279	289	-0.004	0.005	-0.032	-0.027	-0.011	0.066	-0.100	-0.087	0.060	-0.152	-0.028	0	0.882
263	290	0.093	0.090	-0.332	-0.169	-0.094	-0.178	-0.172	0.023	0.262	0.116	-0.036	0	0.996
188	291	0.004	-0.055	-0.247	-0.075	0.059	-0.023	-0.004	0.090	-0.048	-0.090	-0.039	0	0.971
307	292	-0.047	0.055	-0.190	0.054	-0.094	-0.154	-0.142	0.007	0.026	-0.014	-0.050	0	0.899
random	293	0.070	-0.261	-0.106	-0.240	-0.073	-0.066	0.014	0.107	0.119	-0.088	-0.052	0	0.959
141	294	0.077	-0.201	-0.019	-0.186	-0.080	-0.138	-0.063	-0.047	-0.160	0.247	-0.057	0	0.928
195	295	-0.091	-0.051	-0.043	-0.106	-0.064	-0.048	-0.160	-0.017	-0.034	-0.031	-0.065	0	0.896
193	296	-0.117	-0.070	0.009	-0.102	-0.020	-0.198	-0.117	-0.071	-0.057	0.013	-0.073	0	0.921
23	297	-0.073	-0.272	-0.046	-0.136	-0.003	-0.031	-0.097	-0.157	-0.115	-0.031	-0.096	0	0.965
194	298	-0.044	-0.172	-0.072	-0.123	-0.158	-0.150	-0.004	-0.200	-0.047	-0.011	-0.098	0	0.926
52	299	-0.145	-0.010	-0.146	-0.311	-0.097	-0.036	-0.066	-0.214	-0.011	0.024	-0.101	0	0.985
274	300	-0.084	-0.043	-0.079	-0.187	-0.167	-0.051	-0.147	-0.196	-0.029	-0.092	-0.107	0	0.922
209	301	-0.131	-0.047	-0.162	-0.138	-0.245	-0.010	-0.023	-0.076	-0.102	-0.147	-0.108	0	0.979
310	302	-0.192	-0.099	-0.128	-0.103	-0.020	-0.140	-0.098	-0.108	-0.089	-0.127	-0.110	0	0.909
325	303	-0.176	-0.241	-0.054	-0.114	-0.318	-0.045	-0.055	-0.060	-0.124	0.060	-0.113	0	1.000
232	304	-0.175	-0.132	-0.071	-0.040	-0.056	-0.226	-0.095	-0.051	-0.218	-0.084	-0.115	0	0.963
311	305	-0.151	-0.120	-0.140	-0.180	-0.131	-0.015	-0.076	-0.035	-0.174	-0.145	-0.117	0	0.903
104	306	-0.031	-0.136	-0.161	-0.179	-0.153	-0.033	-0.089	-0.103	-0.235	-0.087	-0.121	0	0.963
275	307	-0.184	-0.131	-0.161	-0.106	-0.102	-0.015	-0.196	-0.144	-0.023	-0.153	-0.121	0	0.930
62	308	-0.033	-0.201	-0.109	-0.299	0.002	-0.130	-0.004	-0.158	-0.152	-0.174	-0.126	0	0.978
72	309	-0.095	-0.168	-0.170	-0.196	-0.069	-0.060	-0.100	-0.190	-0.125	-0.100	-0.127	0	0.915
43	310	-0.103	-0.032	-0.139	-0.173	-0.032	-0.218	-0.130	-0.110	-0.153	-0.218	-0.131	0	0.952
309	311	-0.121	-0.113	-0.133	-0.177	-0.083	-0.183	-0.111	-0.096	-0.168	-0.144	-0.133	0	0.912
61	312	-0.172	-0.198	-0.035	-0.131	-0.116	-0.125	-0.143	-0.187	-0.048	-0.198	-0.135	0	0.931
197	313	-0.135	-0.188	-0.098	-0.190	-0.194	-0.156	-0.125	-0.018	-0.141	-0.118	-0.136	0	0.941
73	314	-0.162	-0.286	-0.104	-0.153	0.051	-0.208	-0.128	-0.108	-0.147	-0.135	-0.138	0	0.981
149	315	0.160	-0.310	-0.190	-0.204	-0.123	-0.172	-0.179	-0.136	-0.105	-0.177	-0.144	0	0.996
89	316	-0.180	-0.079	-0.145	-0.201	-0.137	-0.091	-0.169	-0.043	-0.097	-0.297	-0.144	0	0.996
312	317	-0.126	-0.099	-0.217	-0.219	-0.157	-0.156	-0.081	-0.085	-0.191	-0.177	-0.151	0	0.940
196	318	-0.108	-0.080	-0.229	-0.104	-0.187	-0.030	-0.269	-0.202	-0.232	-0.075	-0.152	0	0.990
181	319	-0.052	-0.138	-0.240	-0.110	-0.304	-0.135	-0.237	-0.147	-0.151	-0.075	-0.159	0	0.996
214	320	-0.070	-0.162	-0.224	-0.164	-0.041	-0.122	-0.197	-0.244	-0.239	-0.154	-0.162	0	0.972
254	321	-0.127	-0.199	-0.242	-0.136	-0.068	-0.101	-0.170	-0.232	-0.167	-0.199	-0.164	0	0.967
318	322	-0.221	-0.174	-0.238	-0.165	-0.185	-0.216	-0.140	-0.109	-0.129	-0.065	-0.164	0	0.959
315	323	-0.208	-0.148	-0.312	-0.185	-0.108	-0.106	-0.130	-0.111	-0.253	-0.117	-0.168	0	0.991
320	324	-0.232	-0.166	-0.116	-0.188	-0.165	-0.175	-0.082	-0.228	-0.196	-0.143	-0.169	0	0.964
302	325	-0.184	-0.278	-0.119	-0.077	-0.106	-0.173	-0.230	-0.209	-0.167	-0.149	-0.169	0	0.977
235	326	-0.173	-0.132	-0.209	-0.198	-0.175	-0.151	-0.068	-0.246	-0.237	-0.114	-0.170	0	0.970
223	327	-0.354	-0.091	-0.160	-0.181	-0.173	-0.174	-0.171	-0.164	-0.136	-0.105	-0.171	0	0.988
221	328	-0.162	-0.172	-0.220	-0.219	-0.103	-0.165	-0.184	-0.258	-0.183	-0.046	-0.171	0	0.981
316	329	-0.217	-0.221	-0.216	-0.192	-0.154	-0.204	-0.079	-0.195	-0.118	-0.138	-0.173	0	0.939
319	330	-0.202	-0.147	-0.210	-0.232	-0.161	-0.171	-0.188	-0.185	-0.140	-0.141	-0.178	0	0.951
115	331	-0.232	-0.227	-0.162	-0.076	-0.252	-0.169	-0.057	-0.168	-0.252	-0.190	-0.179	0	0.986
313	332	-0.197	-0.099	-0.167	-0.370	-0.183	-0.222	-0.117	-0.127	-0.105	-0.199	-0.179	0	1.000
44	333	-0.182	-0.133	-0.212	-0.180	-0.141	-0.312	-0.202	-0.194	-0.181	-0.089	-0.183	0	0.996
317	334	-0.230	-0.205	-0.268	-0.234	0.001	-0.187	-0.217	-0.173	-0.160	-0.181	-0.185	0	0.974
38	335	-0.214	-0.200	-0.237	-0.215	-0.119	-0.234	-0.138	-0.250	-0.097	-0.155	-0.186	0	0.976
163	336	-0.250	-0.274	-0.193	-0.153	-0.140	-0.177	-0.196	-0.223	-0.069	-0.237	-0.191	0	0.973
132	337	-0.227	-0.158	-0.192	-0.316	-0.225	-0.071	-0.214	-0.229	-0.085	-0.208	-0.193	0	0.989
314	338	-0.201	-0.186	-0.136	-0.253	-0.186	-0.239	-0.315	-0.132	-0.163	-0.135	-0.195	0	1.000
252	339	-0.201	-0.309	-0.207	-0.212	-0.218	-0.288	-0.136	-0.167	-0.195	-0.045	-0.198	0	0.992
231	340	-0.224	-0.028	-0.089	-0.279	-0.098	-0.220	-0.239	-0.326	-0.230	-0.255	-0.199	0	1.000
154	341	-0.266	-0.108	-0.156	-0.193	-0.202	-0.228	-0.225	-0.219	-0.243	-0.157	-0.200	0	0.977
160	342	-0.172	-0.130	-0.270	-0.169	-0.185	-0.313	-0.236	-0.132	-0.264	-0.216	-0.209	0	1.000
153	343	-0.287	-0.272	-0.082	-0.128	-0.235	-0.233	-0.227	-0.148	-0.198	-0.294	-0.210	0	0.992
144	344	-0.219	-0.175	-0.343	-0.167	-0.171	-0.214	-0.236	-0.132	-0.174	-0.307	-0.214	0	1.000
229	345	-0.160	-0.177	-0.215	-0.326	-0.243	-0.142	-0.237	-0.254	-0.148	-0.270	-0.217	0	0.996
40	346	-0.187	-0.273	-0.287	-0.095	-0.252	-0.170	-0.312	-0.231	-0.210	-0.184	-0.220	0	0.997
107	347	-0.412	-0.201	-0.182	-0.226	-0.239	-0.183	-0.280	-0.149	-0.169	-0.271	-0.231	0	0.996
324	348	-0.181	-0.106	-0.219	-0.303	-0.235	-0.205	-0.252	-0.311	-0.244	-0.290	-0.235	0	0.993

251	349	-0.282	-0.261	-0.324	-0.297	-0.266	-0.207	-0.258	-0.316	-0.096	-0.258	-0.257	0	0.996
92	350	-0.445	-0.307	-0.280	-0.285	-0.194	-0.300	-0.184	-0.169	-0.263	-0.266	-0.269	0	1.000
58	351	-0.382	-0.365	-0.175	-0.324	-0.270	-0.269	-0.267	-0.265	-0.203	-0.230	-0.275	0	1.000

[a] Residues identified by red-colored text had previously been experimentally characterized as being significant in determining the substrate specificty of acyl-ACP TE (Jing et al., 2018)

Supplemental Table 4b. Comparison of model performance between every two adjacent random forest classification models. A series of random forest models has been constructed, where in each consecutive model one additional residue is considered according to the rank of importance scores for each residue, as evaluated in Supplemental Table S4a. MCC scores were compared by Student's t-test between every

Comparison between two adjacent models (A vs B) [a]	Average MCC for Model A [b]	Average MCC for Model B	Delta	p-value	Corrected p-value [c]	significance between Models A vs B [d]
2 vs 3	0.214	0.246	-0.031	6.48E-11	4.17E-10	***
3 vs 4	0.246	0.396	-0.150	1.12E-24	6.52E-23	***
4 vs 5	0.396	0.545	-0.149	8.45E-23	2.45E-21	***
5 vs 6	0.545	0.588	-0.043	3.35E-19	3.88E-18	***
6 vs 7	0.588	0.611	-0.023	1.34E-07	6.49E-07	***
7 vs 8	0.611	0.579	0.032	1.41E-08	7.44E-08	***
8 vs 9	0.579	0.674	-0.095	1.70E-21	3.28E-20	***
9 vs 10	0.674	0.688	-0.014	2.24E-07	9.97E-07	***
10 vs 11	0.688	0.770	-0.082	2.69E-21	3.90E-20	***
11 vs 12	0.770	0.724	0.046	7.39E-13	6.13E-12	***
12 vs 13	0.724	0.709	0.015	1.92E-04	4.46E-04	**
13 vs 14	0.709	0.745	-0.036	2.42E-09	1.40E-08	***
14 vs 15	0.745	0.752	-0.007	3.08E-02	4.58E-02	*
15 vs 16	0.752	0.765	-0.013	1.73E-04	4.18E-04	**
16 vs 17	0.765	0.806	-0.041	1.83E-13	1.77E-12	***
17 vs 18	0.806	0.790	0.016	1.91E-05	5.55E-05	***
18 vs 19	0.790	0.772	0.017	2.04E-04	4.56E-04	**
19 vs 20	0.772	0.807	-0.035	9.58E-13	6.95E-12	***
20 vs 21	0.807	0.809	-0.002	4.03E-01	4.59E-01	NS
21 vs 22 [e]	0.809	0.822	-0.013	1.61E-05	4.93E-05	***
22 vs 23	0.822	0.807	0.015	1.17E-06	4.86E-06	***
23 vs 24	0.807	0.817	-0.009	1.23E-04	3.24E-04	**
24 vs 25	0.817	0.814	0.003	2.32E-01	2.70E-01	NS
25 vs 26	0.814	0.818	-0.004	1.66E-01	2.10E-01	NS
26 vs 27	0.818	0.819	-0.001	7.14E-01	7.53E-01	NS
27 vs 28	0.819	0.819	0.000	9.29E-01	9.33E-01	NS
28 vs 29	0.819	0.810	0.009	3.95E-03	7.63E-03	*
29 vs 30	0.810	0.827	-0.016	9.90E-04	2.05E-03	*
30 vs 31	0.827	0.810	0.017	2.32E-04	4.98E-04	**
31 vs 32	0.810	0.825	-0.015	2.12E-06	7.25E-06	***
32 vs 33	0.825	0.817	0.008	7.52E-03	1.30E-02	*
33 vs 34	0.817	0.826	-0.009	1.72E-02	2.70E-02	*
34 vs 35	0.826	0.831	-0.005	1.02E-01	1.34E-01	NS
35 vs 36	0.831	0.811	0.020	1.61E-04	4.06E-04	**
36 vs 37	0.811	0.810	0.002	6.66E-01	7.28E-01	NS
37 vs 38	0.810	0.819	-0.009	7.38E-03	1.30E-02	*
38 vs 39	0.819	0.805	0.014	3.16E-06	1.02E-05	***
39 vs 40	0.805	0.797	0.008	2.12E-02	3.23E-02	*

40 vs 41	0.797	0.806	-0.009	3.89E-02	5.64E-02	NS
41 vs 42	0.806	0.784	0.022	1.46E-06	5.61E-06	***
42 vs 43	0.784	0.811	-0.026	1.55E-06	5.61E-06	***
43 vs 44	0.811	0.818	-0.007	6.04E-02	8.55E-02	NS
44 vs 45	0.818	0.816	0.001	7.09E-01	7.53E-01	NS
45 vs 46	0.816	0.810	0.007	1.24E-01	1.59E-01	NS
46 vs 47	0.810	0.790	0.020	4.87E-05	1.34E-04	**
47 vs 48	0.790	0.794	-0.005	1.90E-01	2.29E-01	NS
48 vs 49	0.794	0.803	-0.008	9.34E-02	1.26E-01	NS
49 vs 50	0.803	0.818	-0.015	2.53E-03	5.05E-03	*
50 vs 51	0.818	0.830	-0.012	1.22E-02	1.97E-02	*
51 vs 52	0.830	0.824	0.006	1.80E-01	2.23E-01	NS
52 vs 53	0.824	0.817	0.008	8.16E-02	1.13E-01	NS
53 vs 54	0.817	0.833	-0.016	4.47E-03	8.36E-03	*
54 vs 55	0.833	0.830	0.002	6.21E-01	6.92E-01	NS
55 vs 56	0.830	0.840	-0.010	7.65E-03	1.30E-02	*
56 vs 57	0.840	0.846	-0.006	9.27E-03	1.54E-02	*
57 vs 58	0.846	0.846	0.000	9.33E-01	9.33E-01	NS
58 vs 59	0.846	0.847	0.000	9.14E-01	9.33E-01	NS
59 vs 60	0.847	0.842	0.005	1.94E-01	2.29E-01	NS

[a] Each consecutive model (A vs B) considered one additional residue according to the ranking of importance scores for each residue (see Table S4a)
[b] Matthew's correlation coefficient for Model A and B are subjected to z transformation for Student's t
[c] The p-values are corrected among all comparisons to control the false discovery rate < 5%.
[d] The scale of p-values is indicated by the number of asterisks: *, p < 0.05; **, p <0.001; ***, p <0.0001; NS, not-significant.
[e] The model including top 22 residues reaches the statistical plateau of MCC score.
